# Reinfection dynamics of owned dogs with *Echinococcus granulosus* sensu lato in endemic rural areas of central Iran: Implications for cystic echinococcosis control

**DOI:** 10.1371/journal.pntd.0014451

**Published:** 2026-06-29

**Authors:** Seyed Reza Mirbadie, Saeedeh Shamsaddini, Mohammad Taghi Rahimi, Mohammad Matini, Mohammad Ali Mohaghegh, Mohammad Reza Baneshi, Majid Fasihi Harandi, Mohammad Fallah

**Affiliations:** 1 Department of Medical Parasitology and Mycology, School of Medicine, Hamadan University of Medical Sciences, Hamadan, Iran; 2 School of Medicine, Shahroud University of Medical Sciences, Shahroud, Iran; 3 Research Center for Hydatid Disease in Iran, Institute of Infectious Diseases and Tropical Medicine, Afzalipour School of Medicine, Kerman University of Medical Sciences, Kerman, Iran; 4 Department of Laboratory Sciences, School of Paramedical Sciences, Torbat Heydariyeh University of Medical Sciences, Torbat Heydariyeh, Iran; 5 The University of Queensland, Australian Women and Girls’ Health Research Centre, School of Public Health, Herston, Queensland, Australia; Rio de Janeiro State University: Universidade do Estado do Rio de Janeiro, BRAZIL

## Abstract

**Background:**

Cystic echinococcosis (CE), caused by the tapeworm *Echinococcus granulosus* sensu lato (*E. granulosus* s. l.), is highly endemic worldwide including in Iran, where dogs serving as definitive hosts play a critical role in transmission dynamics of the disease. Regular deworming of dogs is an essential part of any successful CE control program. However, dogs can become easily reinfected after praziquantel (PZQ) administration, and the rate of reinfection of dogs in an endemic area is considered an indicator of the intensity of CE transmission. This study aimed to evaluate reinfection rate in the owned dogs after PZQ treatment in Semnan province, in North of Iran.

**Methods:**

A cohort of 381 guard and sheepdogs was monitored for 15 months post-treatment with PZQ. Reinfection was assessed at 3, 6, 9, 12, and 15 months by fecal sampling. Samples were microscopically examined for taeniid eggs after formalin-ether sedimentation, and were confirmed by PCR using *E. granulosus* s. l.-specific primers. Kaplan-Meier survival analysis was used to calculate the cumulative survival rate over a 15-month period. A Cox proportional hazards regression model was employed to identify risk factors associated with the time to reinfection with *E. granulosus* s.l.

**Results:**

Fecal samples from 151 sheepdogs and 230 guard dogs from 290 farms, were examined. At baseline, microscopic and copro-PCR analyses revealed that 12.6% positive for taeniid eggs, of which 31 samples (8.1%) were positive for *E. granulosus* s. l. The reinfection rate was estimated as 9.6%, 10.7%, 15.6%, 22.5%, and 33.2% at 3, 6, 9, 12, and 15 months after initial treatment with PZQ, respectively. Sheepdogs showed significantly higher reinfection risks (29.8%) compared to the guard dogs (16.7%). Dogs whose owners practiced home slaughter were 2.02 times more likely to become reinfected. Only 17.3% (66/381) of the dogs were reported to be regularly dewormed by the owners.

**Conclusion:**

This study demonstrates that re-infection of dogs with *E. granulosus* s.l. is common in CE-endemic regions of Iran. The high rate of re-infection has significant implications for CE control, particularly in determining the most effective frequency for administering PZQ to prevent sustained transmission.

## Author summary

Cystic echinococcosis is a long-standing disease that continues to affect people and livestock in many rural regions of the world. Dogs are essential for the survival and transmission of the parasite, and effective control of infection in dogs is central to preventing human disease. However, dogs often become infected again soon after treatment, which makes disease control extremely difficult. In this study, we followed more than three hundred farm dogs in northern Iran for fifteen months after treatment to understand how quickly and how frequently this reinfection occurs.

We found that nearly one third of treated dogs became infected again within this period. Sheepdogs and the dogs whose owners practiced home slaughter were significantly at higher reinfection risks. Only a small fraction of dog owners treated their animals regularly, which further increased the chance of reinfection. By measuring the time until dogs became infected again, we were able to identify the practices that maintain disease transmission in these communities. Our findings show that treating dogs alone is not sufficient. Sustainable control of cystic echinococcosis requires changes in animal husbandry, safer disposal of animal remains, and improved community awareness to reduce long-term risk for both animals and people.

## Introduction

Cystic Echinococcosis (CE), caused by the small tapeworm of canids *Echinococcus granulosus* sensu lato (*E. granulosus* s. l.), is a major parasitic zoonosis of humans worldwide, with livestock and dogs serving as the intermediate and definitive hosts, respectively [1]. Currently, more than one million people worldwide are infected with CE, and this condition is considered a serious public health challenge [1,2]. The impact of this disease is primarily on the marginalized and rural communities with low socioeconomic status and inadequate management and control over dogs.

Global reports of the incidence of CE have estimated 207,368 new cases with 122,457 DALYs [3]. The surgical incidence of CE varies between 1 and 200 cases per 100,000 people worldwide. CE is endemic with high incidence in most Middle East and North African countries including Iran, where the incidence is estimated 0.61-1.27 per 100,000 [4]. Considering the close relationship between dogs and humans, dogs play a key role in CE transmission. The overall estimated prevalence of infection in dogs in Iran is 23.6% [5,6]. CE imposes substantial economic and social burdens on affected communities, encompassing the costs of medical and surgical management and the resulting disability in humans, as well as significant losses to the livestock sector [7,8]. In Iran, the overall annual monetary burden of the disease has been estimated at approximately US$232 million [9,10].

The role of dogs as the main definitive host and reservoir of the parasite is crucial in the spread of parasite eggs in the environment through canine feces. Guard dogs and sheepdogs are essential in the activity of the people living in the rural and semi-rural regions in Iran, where agriculture and animal husbandry is their major occupation [11,12]. One of the key factors in CE transmission in most parts of the world is the close relationship of dogs, livestock, and humans. Measures for CE control include livestock vaccination, regular dog deworming, dog registration, dog population management, improving abattoir infrastructure, controlling home slaughter and dog access to livestock offal [3,12,13]. Echinococcosis in dogs is very difficult to control and to date, only a limited number of control programs in a few countries have been successful [14,15]. Various studies have shown that regular deworming of dogs plays a substantial role in reducing the incidence of canine echinococcosis [12,16,17]. Reinfection of dogs remains one of the major obstacles to controlling CE in endemic communities. Although dog deworming is the central and most effective intervention for interrupting transmission, its impact is often undermined because dogs frequently become reinfected when they continue to access contaminated offal or roam freely.

Dog deworming campaigns have been hampered by frequent reinfection of dogs following access to the infected livestock offal. Dogs can become easily reinfected after praziquantel (PZQ) administration, and the rate of reinfection of dogs in an endemic area is considered an indicator of the intensity of CE transmission [18,19]. This constant reinfection cycle is a key reason why many CE control programs fail to achieve long-lasting results. However, our understanding of how often dogs are reinfected, how quickly this occurs, and which community practices contribute to it is still limited. Without reliable knowledge of reinfection dynamics, it is not possible to design effective interventions [18]. These gaps highlight the urgent need for field studies in the endemic areas on dog reinfection to support the development of successful and sustainable CE control strategies. Studies conducted in two North African countries, Morocco and Tunisia, demonstrated that administering treatment to dogs at two-month intervals was more effective in controlling reinfection compared to less frequent regimens [11,20].

Studying dog re‑infection with *E. granulosus* in endemic regions of Iran has important implications for the prevention and control of CE, as re‑infected dogs serve as continuous sources of environmental contamination and sustain transmission to livestock and humans. Understanding the local re‑infection dynamics is essential for designing effective and region‑specific control measures. In Iran, despite being a country with diverse ecological zones and varying animal husbandry systems, only one study has investigated dog re‑infection, and it was performed in the southeastern region. In a recent study in southeast of the country, reinfection of owned dogs reached 17% one year after initial PZQ treatment with a monthly reinfection incidence of 1.5% [18]. However, re‑infection rates can vary widely depending on cultural practices, livestock management systems, slaughterhouse hygiene, the availability of infected offal, and dog feeding behaviors, meaning that findings from one area cannot safely be generalized to another. Conducting multiple studies within each endemic region allows researchers and public health authorities to determine how quickly dogs become re‑infected after deworming, identify high‑risk practices responsible for ongoing transmission, and evaluate whether current intervention programs such as routine PZQ dosing or public education, are sufficient.

Findings of one single study cannot represent the complexities of transmission across the entire country, leaving substantial gaps in our understanding of how the parasite persists in farm and guard dogs in other provinces. Addressing these gaps through region‑specific re‑infection studies is necessary to build a comprehensive national strategy, optimize surveillance, and tailor interventions to local risk factors, ultimately improving efforts to reduce both animal and human echinococcosis.

This study was designed to quantify how often reinfection occurs in owned dogs and identify the contributing factors in endemic communities. Specifically, we aimed to estimate the cumulative incidence of *E. granulosus* sensu lato reinfection after PZQ treatment and examine demographic, behavioral, and environmental variables that may increase reinfection risk. This approach was intended to generate evidence needed to inform more effective deworming strategies.

## Materials and methods

### Ethics statement

The project was approved by the ethical review committee of Hamadan University of Medical Sciences (IR.UMSHA.REC.1401.877). The study objectives were fully explained to the dog owners, emphasizing the importance of the study. Prior to sampling, verbal consent was obtained from the owners of the dogs. With the owners’ consent, all dogs present at each respective farm or herd were sampled, which were primarily working animals such as sheepdogs and guard dogs.

### Study area

**Fig 1:**
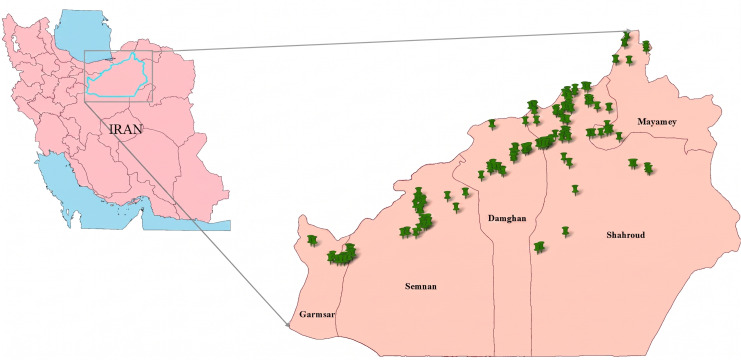
Distribution of the sampled farms in Semnan province, Iran, showing 290 farms to assess *Echinococcus granulosus sensu lato* reinfection in 381 dogs. The map is produced by ESRI’s ArcGIS Desktop ver. 10.8.2 using the shapefile available online from https://data.humdata.org/dataset/09c2c869-44c5-4e92-8252-d1215b4e85c8. The map is intended exclusively for data visualization and presentation of research findings. It will not be used for commercial purposes, redistribution, or resale, and its use is fully consistent with the provisions of ESRI’s Master Agreement.

This cross-sectional study was conducted in Semnan Province, Iran. Semnan is located between the edge of the desert plain (central desert of Iran) and the Alborz Mountain range with a high-altitude variation between 1000 and 3000 m above sea level. The province is located between latitude 34°13′-37°20′N and longitude 51°51′-57°3′E in north-central Iran with a surface area of 97,491 Km^2^ and a population of 702,360 (Fig 1). Semnan has a diverse climate with a temperate climate in the northern areas and arid and semi-arid regions in the south. The average annual rainfall is 139.5 mm. (https://www.worldweatheronline.com/semnan-weather-averages/semnan/ir.aspx) with 13.6 million acres of pasture and 2.5 million livestock (https://amar.org.ir/) (https://semnan-aj.ir/).

### Study design and sample collection

**Fig 2:**
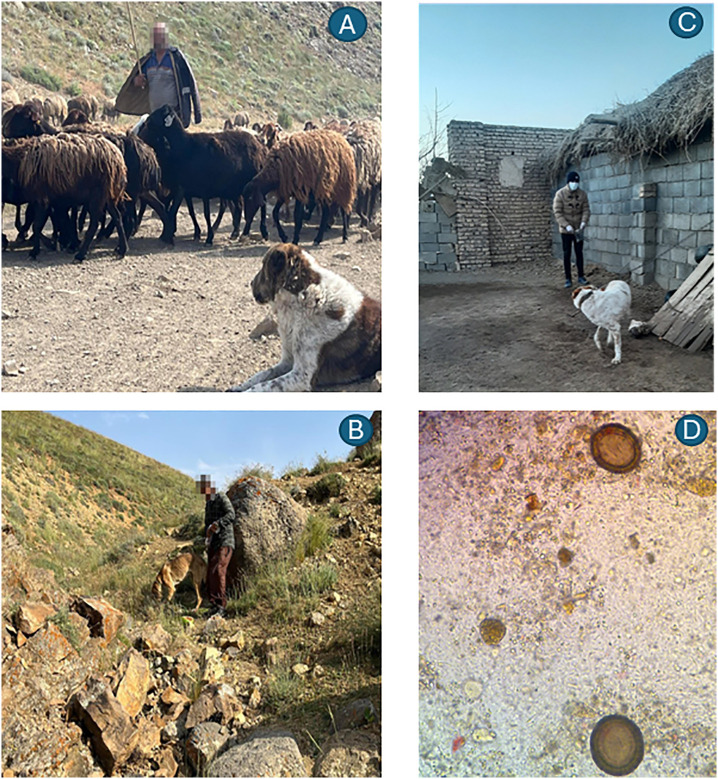
Field procedures and initial microscopic diagnosis (A–B) Representative sheepdogs, illustrating the typical definitive host population for *Echinococcus granulosus* sensu lato in the endemic areas of Garmsar (A) and Semnan (B) districts. **(C)** Field collection of fresh fecal sample from a guard dog in Shahroud district. **(D)** Microscopic visualization of a characteristic taeniid egg detected in a canine fecal sample during primary parasitological examination. These figures were created by the authors and is published under CC BY 4.0.

In total, 290 farms from 101 villages were randomly selected from five counties in Semnan province, including Semnan, Shahroud, Damghan, Mayamey, and Garmsar. A total of 381 dogs were recruited in the study (Fig 1). The living place of the dogs and their roaming status were recorded and sampling was done as follows. For those dogs that were tied up constantly or only during the day, the fresh feces samples were collected from inside and/or around their living place. Most samples from herding dogs were collected from their resting areas. Shepherds and dog owners were asked to collect fecal samples from each dog immediately after defecation. Some dogs were also restrained or tied up until defecation and sample collection (Fig 2).

Approximately 100 grams of stool sample from each dog were placed in plastic containers in cold boxes and transferred promptly to the laboratory for parasitological examination. Half of the collected sample was preserved in 10% formalin for subsequent sedimentation testing and microscopic examination, while the remaining half was designated for DNA extraction and molecular work. For safety reasons, all samples were kept at -80°C for at least ten days.

### Parasitological and molecular analyses

All samples were microscopically examined for taeniid eggs following formalin-ether concentration technique. Subsequently, taeniid eggs were recovered from fecal material using sugar flotation. Sugar flotation method was applied to the positive samples as described elsewhere with some modifications [21]. Briefly, high density sugar solution with a specific gravity of 1.27 was added to each sample and the resulting suspension was centrifuged at 500 g for 10 min. In the next step, taeniid eggs were collected from the supernatant and stored in -20 °C until used for molecular analysis.

All dogs found infected with taeniid eggs were treated with PZQ, 5 mg/kg of body weight. Seven days after anthelmintic treatment, re-sampling was done to check the outcome of the treatment. Re-treatment was performed until a negative result was obtained. In total, only 4 dogs were positive 7 days after the initial treatment, which were re-treated and tested negative for infection after re-sampling. Re-samplings were performed to investigate the re-infection rate of the dogs at 3, 6, 9, 12, and 15 months after the initial administration of PZQ. The dogs did not receive any further treatment during the entire study period.

For molecular analysis, PCR-sequencing was performed using three primer pairs amplifying partial cytochrome c oxidase (COX1) gene (444 bp), a 133-bp fragment of a tandem repeat sequence, and a partial SSU-rDNA region (149 bp). The cox1 primer pair was taeniid-specific and the other two primer sets were used for the specific detection of *E. granulosus* s. l. [18,22,23]. Table 1 shows the primer sequences used in the present study. Previously sequenced DNA samples were used as positive controls [7].

**Table 1:** The primer sequences used in the present study for the diagnosis of cystic canine echinococcosis in Semnan province, Iran.

Primer	Name	Sequence (5´ > 3´)	Length	Genetic marker	Reference
Forward (F)	JB3	TTTTTTGGGCATCCTGAGGTTTAT	24	Cox1	**[39]**
Reverse (R)	JB4.5	TAAAGAAAGAACATAATGAAAATG	24		
Forward (F)	Eg1121a	GAATGCAAGCAGCAGATG	18	133-bp tandem repeat unit	**[22]**
Reverse (R)	Eg1122a	GAGATGAGTGAGAAGGAGTG	20		
Forward (F)	EGfor1	GTGTGTTACATTAATAAGGGTG	22	SSU-rDNA	[23]
Reverse (R)	EGrev1	CTTGTTACGACTTACCTCAA	20		

For genotype identification, the PCR products obtained from *E. granulosus* s. l.-positive samples were randomly selected, purified and sent for Sanger sequencing (Pishgam co. Tehran) by targeting SSU-rDNA using the reverse primer (Table 1).

The sequence data were edited and analyzed by Sequencher 4.7 and MEGA 5.05. The obtained sequences were examined using the NCBI BLAST database for the final identification and confirmation of *E. granulosus* s. l. genotypes.

### Dog data collection

A data collection sheet was filled for each dog before fecal sampling. Data related to each dog were recorded including age, sex, location, anthelmintic treatment, type of dog food, and roaming habits. Specific dog characters were recorded for identification of dogs during follow-up. In addition, dog owners were asked about home slaughter and disposal of sheep carcasses and infected organs.

### Statistical analysis

The Kaplan-Meier method was used to calculate and visualize the cumulative survival rate stratified by age, sex, and type of dog. Cumulative incidence was defined as the complement of survival rates (i.e., one minus survival rates). Time was considered as the variable of infection time for dogs with reinfection, and the time of the last microscopic examination was defined as the censored time for uninfected dogs.

To identify risk factors associated with the time to reinfection with *E. granulosus* s. l., a Cox hazards regression model was employed. In total, data on eight independent variables were collected. It has been shown that regression models provide stable estimates if the sample size per number of coefficients is at least 10. Given the adequate sample size, we included all variables in the model to make a full adjustment. Thus, eight variables including gender, age, dog type, roaming behavior, feeding habits, anthelmintic treatment, location, and home slaughter were evaluated using Cox analysis. Adjusted Hazard Ratios (HRs) and their 95% confidence intervals (CIs) were estimated to quantify the magnitude of association between each covariate and the risk of reinfection. The variance inflation factor (VIF) was calculated, and no serious multicollinearity between independent variables was observed. The proportional hazard assumption was tested using Schoenfeld residual plots and no departure from PH assumption was seen. All statistical analyses were conducted using R software (S1 File).

## Results

### Study population, baseline prevalence and genotyping

Fecal samples from a total of 381 owned dogs, including 151 sheepdogs and 230 guard dogs from 290 farms, were collected between February 2023 and May 2024 in different villages in five counties of Semnan province (Fig 1). Over a 15-month period, the dogs were evaluated for re-infection with *E. granulosus* s. l. Microscopic study and copro-PCR analyses showed a total of 48 samples (12.6%) as positive for taeniid eggs before the start of the deworming program, of which 31 samples (8.1%) were positive for *E. granulosus s. l*. Taeniid eggs were detected in 20 guard dogs and 28 sheepdogs, among which *E. granulosus* s. l. was identified in 14 (6.1%) and 17 (11.2%) of individuals, respectively (Table 2, Fig 2). Nine *E. granulosus* s. l.-positive samples were PCR-sequenced on a partial SSU-rDNA (~149 bp) and compared with the sequences in NCBI GenBank. Eight isolates were identified as *E. granulosus* sensu stricto (s.s.) (G1-G3) and one as *E. canadensis* G6-G7.

**Table 2:** The frequency of Taeniidae/ *Echinococcus granulosus sensu lato* eggs in the owned dogs of Semnan province, Iran, before anthelmintic treatment, using microscopy and copro-PCR techniques.

Study areas		No. of fecal samples examined	Type of dogs		Microscopic observation	Copro-PCR
			Sheepdog	Guard dog	No. of positive samples, taeniidae eggs (%)	No. of positive samples, E. granulosus sensu lato (%)
Central Iran	Mayamey	43	18	25	7 (16.3%)	4 (9.3%)
	Shahrood	119	46	73	14 (11.8%)	9 (7.5%)
	Damghan	97	38	59	11 (11.3%)	7 (7.2%)
	Semnan	64	24	40	6 (9.4%)	4 (6.2%)
	Garmsar	58	25	33	10 (17.2%)	7 (12%)
	**Total**	**381**	**151**	**230**	**48 (12.6%)**	**31 (8.1%)**

### Reinfection dynamics and survival analysis

Following initial treatment with PZQ, the cohort of dogs was monitored for reinfection over a 15-month period. Table 3 presents the survival rates and cumulative incidence of infection at 3, 6, 9, 12, and 15 months post-treatment with PZQ. The cumulative incidence of reinfection with *E. granulosus* s. l. progressively increased throughout the follow-up. At 3 months post-treatment, the cumulative incidence was 9.6% (95% CI: 6.5-12.5%). This rate rose to 10.7% (95% CI: 7.5-13.7%) at 6 months, 15.6% (95% CI: 11.9-19.1%) at 9 months, and 22.5% (95% CI: 18.0-26.8%) at the 12-month mark. By the end of the 15-month study period, the cumulative incidence of reinfection reached 33.2% (95% CI: 27.7-38.3%) (Table 3).

**Table 3:** Survival rate estimates among dogs reinfected with *Echinococcus granulosus sensu lato* in Semnan province, Central Iran.

Time post-treatment (month)	All dogs, % (95% CI)	Guard dogs, % (95% CI)	Sheepdogs, % (95% CI)
**3**	90.4 (87.5, 93.5)	92.0 (88.5, 95.7)	88.3 (83.5, 93.4)
**6**	89.3 (86.2, 92.5)	91.5 (87.8, 95.4)	86.4 (81.3, 91.8)
**9**	84.4 (80.8, 88.2)	88.4 (84.1, 92.9)	79.4 (73.3, 85.9)
**12**	77.5 (73.2, 82.0)	83.3 (78.2, 88.7)	70.2 (63.3, 77.9)
**15**	66.8 (61.6, 72.3)	75.4 (69.2, 82.2)	55.8 (47.9, 65.0)

**Fig 3:**
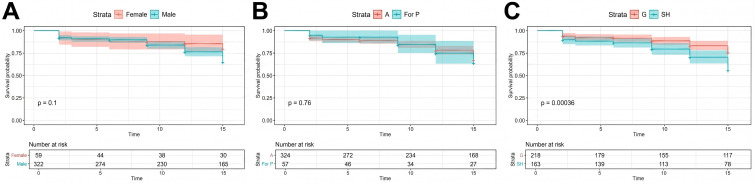
Kaplan-Meier Survival rate of the owned dogs reinfected with *Echinococcus granulosus* sensu lato. The cumulative survival rate stratified by sex **(A)**, age **(B)**, and dog type **(C)**.

Kaplan-Meier survival analysis was used to visualize the time to reinfection (Fig 3). The analysis, when stratified by dog type, showed a significantly lower survival probability (i.e., a higher rate of reinfection) for sheepdogs compared to guard dogs over the 15-month period (Fig 3C). The final reinfection risk was 29.8% for sheepdogs versus 16.7% for guard dogs. No significant differences in survival rates were observed when dogs were stratified by sex or age (Fig 3A, 3B).

### Risk factor analysis for reinfection

A Cox proportional hazards regression model was employed to identify risk factors associated with the time to reinfection. Table 4 shows hazard ratios obtained from analyzing the potential risk of echinococcosis in the owned dogs in rural areas of Semnan province. The analysis revealed several significant predictors. Dog type was a primary risk factor, with sheepdogs having a 1.8 times higher risk of reinfection compared to guard dogs (Adjusted Hazard Ratio [HR]: 1.8, 95% CI: 1.22-2.68; p < 0.05). Practices related to livestock slaughter and feeding were also strongly associated with reinfection. Dogs whose owners practiced home slaughter were 2.02 times more likely to become reinfected (HR: 2.02, 95% CI: 1.35-3.03; p < 0.05). Similarly, feeding dogs with raw livestock offal was identified as a significant risk factor (p = 0.001).

**Table 4:** Cox hazards regression analysis to assess the risk of echinococcosis reinfection in the owned dogs in rural areas of Central Iran.

Variable	Category	Hazard Ratio (HR)	95% CI	P-value
**Gender**	Female	Reference		
	Male	1.47	0.76, 2.85	0.25
**Age**	Adult	Reference		
	Pup/ Young	0.94	0.55, 1.61	0.82
**Dog type**	Guard dog	Reference		
	Sheepdog	1.81	1.22, 2.68	0.003
**Roaming** **behaviour**	Always leashed	Reference		
	Free at Night	1.00	0.57, 1.74	0.99
	Always free	0.85	0.52, 1.38	0.51
**Feeding habit**	Human food	Reference		
	Viscera	2.01	1.30, 3.08	0.001
**Anthelmintic treatment**	No	Reference		
	Yes	0.50	0.27, 0.96	0.03
**Study area**	Shahroud	Reference		
	Mayamey	1.37	0.73, 2.61	0.32
	Damghan	0.99	0.58, 1.69	0.99
	Semnan	0.92	0.50, 1.69	0.79
	Garmsar	1.45	0.82, 2.55	0.19
**Home slaughter**	No	Reference		
	Yes	2.02	1.28, 3.18	0.002

Furthermore, the history of anthelmintic treatment was a significant factor. Dogs that were not regularly dewormed by their owners had a 1.76 times greater risk of reinfection than those receiving regular treatment (HR: 1.76, 95% CI: 1.04-2.97; p < 0.05). Data collected from owners indicated that only 17.3% (66/381) of the dogs in the study were reported to be regularly dewormed prior to the study’s intervention. Other variables, including the dog’s gender, age, roaming behavior, and location, were not found to be significantly associated with the risk of reinfection in the final model (Table 4).

## Discussion

CE is a major parasitic zoonosis and has been classified by the World Health Organization as a Neglected Tropical Disease (NTD), with control efforts prioritized under the 2030 global roadmap [24]. The disease is endemic in Iran, and domestic dogs are the main source of human infection. The prevalence of *E. granulosus* s. l. in dogs has been reported to range from 6.8% to 55.7% in different regions of Iran. According to recent studies, Semnan province in central Iran is one of the important foci of the CE in the country, with a prevalence of 7.4% in dogs, 13.3% in livestock, and 8.6% seroprevalence in humans [4,25].

Dogs become readily re-infected when they consume raw infected offal during home slaughter or scavenging, allowing new adult worms to mature and resume egg shedding. This repeated cycle and continual re-infection creates a persistent environmental source of contamination, highlighting the need for reducing dog re-infection through regular deworming, responsible slaughter practices, and proper offal disposal to break the transmission cycle. Regular dog deworming by the oral administration of PZQ at scheduled intervals is the first option in implementing CE control programs in endemic regions. However, re-infection of dogs after treatment is a major challenge in CE control programs. It has been shown that lack of treatment or inadequate treatment intervals increase the odds of reinfection by 23.3-52.5 [16].

### Evidence generated by the current study

To assess the re-infection rate of echinococcosis in dogs, a 15-month follow-up study was conducted at 3, 6, 9, 12, and 15 months after treatment with PZQ. The rate of reinfection in dogs after treatment increased over time, such that 9.6% (6.5-12.5%) of dogs were reinfected with *E. granulosus* s. l.*,* three months after the initial treatment. The infection rate reached 22.5% (18.0-26.8%) one year after initial deworming (Table 3). The rate of reinfection in owned dogs, particularly guard and sheepdogs, has been investigated in few studies worldwide, including Iran.

Table 5 shows the reinfection studies conducted in different endemic countries. Findings of these studies indicate that in various endemic countries the reinfection rate ranges from 1.56% to 27.9% one year after initial treatment. A study in Libya estimated reinfection in 334 owned dogs. The rate of reinfection 15 months after treatment with PZQ reported to be 22.2% [26]. In Peru it was shown that failure of regular deworming of dogs increases the rate of reinfection [27]. In Uruguay, reinfection in dogs occurred after 2–4 weeks [15]. In the two studies conducted in Morocco and Iran, the monthly reinfection rates in domestic dogs were reported to be 4% and 1.5%, respectively [12,18].

**Table 5:** Summary of the findings of reinfection studies on canine echinococcosis conducted in different endemic countries.

Country	No. samples	Host	Months post treatment, (reinfection rate, %)	Method	Reference
**Iran**	381	owned dogs	3 (9.6%) 6 (10.7%) 9 (15.6%) 12 (22.5%) 15 (33.2%)	Copro-PCR	Present study
**Iran**	150	owned dogs	2 (8) 5 (12) 12 (17)	Copro-PCR	[18]
**Morocco**	595	owned dogs	monthly rate 4%	Copro-PCR	[12]
**China** **(Shiqu county)**	584	owned dogs	3 (13.36) 6 (5.8) 9 (0) 12(1.56)	Copro-ELISA	[40]
**China** **(Seda county)**	584	owned dogs	3 (12.5) 6 (3.13) 9 (0) 12(1.56)	Copro-ELISA	[40]
**China**	281	owned dogs	2 (9.6) 5 (3.1) 12 (11)	Copro-PCR Copro-ELISA	[31]
**Tunisia**	375	semi-stray dogs	2 (6.45) 4 (3.57) 8 (6.25) 12 (16.66)	Flotation	[41]
**Libya**	334	owned dogs	15 (22.2)	Copro-antigen	[26]
**China**	50	owned dogs	3 (25) 7 (29.2) 12 (16.7)	Copro-antigen	[19]
**Uruguay**	367	ND	2 (0) 4 (6.8) 8 (18.6) 12(27.9)	ND	[30]

ND: No data.

One single study was conducted in Iran to investigate reinfection in farm dogs in Kerman, and the reinfection rate one year after the study was 17%. In the present study 22.5% of the dogs were reinfected after one year and 33.2% after 15 months. Interestingly, higher reinfection was observed in the sheep dogs (29.8%) comparing to the guard dogs (16.7%). This is consistent with the findings of other studies conducted in Libya and Kazakhstan. In Libya, herd dogs are 9 times more likely to be copro-Ag positive than domestic dogs [26,28]. Also, findings of another study in Morocco indicate that the reinfection rate in stray dogs was higher than in owned dogs [20]. The higher chance of reinfection in the sheepdogs can be due to a higher chance of feeding with offal, free-roaming behavior, and home slaughter. In addition, while the study focused on owned dogs, the presence of unowned dog populations in the same areas, were not quantified. Free-roaming dog populations frequently evade treatment efforts, while wildlife reservoirs in some regions contribute to environmental contamination [29]. Further studies are required to investigate the nature and extent of reinfection in unowned free-roaming dogs in the endemic regions.

Variations in reinfection rates across different endemic regions may be attributed to differences in animal husbandry and slaughter practices, cyst fecundity, frequency of dog deworming, diversity of intermediate hosts, genotype-related factors, as well as socioeconomic, cultural, and lifestyle differences. Additionally, the use of different diagnostic methods may further contribute to these discrepancies. [30,31].

Due to the lack of longitudinal survival studies, Cox analyses and hazard ratios (HRs) are rarely reported in the studies to understand the factors associated with echinococcosis in dogs. However, risk ratios (RRs) and/or odds ratios (ORs) are frequently used in epidemiological studies to quantify associations between risk factors and canine echinococcosis. In the present study the dog type (guard vs. sheepdog), feeding dogs with livestock viscera, anthelmintic treatment of dogs, and home slaughter practice were significantly associated with the risk of *Echinococcus* infection. Several studies have identified dog-keeping practices, and access to raw livestock offal as the predominant risk factors of canine CE [16,32]. Dogs fed uncooked viscera in Peru exhibit 2.9 times (95% CI, 1.0–8. Also, in Uganda access of dogs to livestock slaughter facilities significantly increased the risk of canine echinococcosis (OR = 11) [33]. Allowing dogs to roam freely (OR=3.17), feeding dogs with viscera (OR=3.04), and slaughter at home (OR=3.53) demonstrate greater odds of *E. granulosus* s. l. infection [34]. This practice significantly sustains parasite lifecycles in the rural environments. Roaming behavior further elevates risk of canine echinococcosis in several studies.

In Libya non-restraint of dogs, home slaughtering of livestock, limited awareness and failure of owners to administer regular deworming treatments, were found as significant risk factors of infection [26]. The results of our study also showed that dogs that were constantly free-roamed accounted for more than half of the positive cases. However, in the present study this was not significantly associated with infection in dogs. It is important to note that dogs kept under constant restraint are very likely to be fed infected offal by their owners.

Our results showed that dogs whose owners practiced home slaughter were 2.02 times more likely to be re-infected with *E. granulosus* s. l. In pastoral and rural communities, home slaughter bypasses veterinary meat inspection, allowing infected organs to be discarded or fed to dogs, with many studies reporting higher odds of canine infection in such settings [19,33,34]. This perpetuates egg contamination of rural environments, increasing risk of ingesting *Echinococcus* eggs by humans. Consequently, home slaughter undermines abattoir regulations and CE control programs.

The results of our questionnaire survey showed that only 39.3% of dog owners had sufficient information about the life cycle and routes of infection in dogs. In a study in rural Peru, more than 23% of dog owners fed offal to their dogs. A study in Libya found that home slaughter increased the risk of copro-antigen positivity of dogs by 8 times. Various studies consider the lack of awareness in dog owners and low knowledge of CE transmission as the main factors of infection in shepherd and domestic dogs [26,27]. Also, socio-cultural traditions and economic factors are important in resource-limited regions. Interventions targeting this practice through community education, subsidized slaughter facilities, and incentivized suitable offal disposal, are critical to disrupt CE transmission. Without addressing home slaughter, sustained reductions in canine and human echinococcosis remain unachievable [35].

Genotype variation of *E. granulosus s.l.* affects deworming effectiveness in dogs. In Semnan province, *E. granulosus s.s.* (G1/G3) and *E. canadensis* (G6/G7) genotypes were found in dogs and livestock. The pre-patent period differs: G1 sheds eggs within six weeks post-infection, whereas G6/G7 requires 45–60 days. This variation has direct implications for re-infection dynamics, as deworming schedules optimized for one genotype may fail to interrupt transmission in areas dominated by the other. Local genotyping is therefore critical for effective control. However it should be noted that due to the limited sample size, these genotyping results are exploratory in nature and are not intended to provide definitive estimates of genotype prevalence or transmission dynamics and the findings require validation in larger, adequately powered studies [7,15,25,36,37].

### Policy implications and recommendations

Repeated re-infection of farm dogs with *E. granulosus* s. l. poses significant challenges for CE control. Consequently, regular and frequent dog deworming is required to overcome the infection pressure in dogs. As definitive hosts, farm dogs in the CE-endemic regions, are particularly vulnerable to re-infection due to their frequent exposure to infected offal from home-slaughtered livestock, scavenging on carcasses, and inadequate access to regular anthelmintic treatment. The frequent re-infections undermine control efforts, as even treated dogs become reinfected when traditional or existing animal husbandry, slaughtering, and dog-keeping behaviors remain unchanged [15,18,19,31]. Moreover, the persistent contamination of pastures, water sources, and farm environments with *Echinococcus* eggs sustains parasite circulation and increase the risk of human infection through accidental ingestion of eggs.

The implementation of regular canine deworming programs as a strategy for CE control faces significant operational and biological challenges that undermine effective CE control measures. The frequency of PZQ dosing is related to dog reinfection pressure. The short prepatent period in canine echinococcosis (5–6 weeks) imply monthly PZQ administration to prevent egg shedding, however, this frequency is logistically demanding and economically unsustainable in endemic communities. Many endemic areas lack adequate veterinary infrastructure, resulting in inconsistent drug distribution and treatment coverage gaps. The European Scientific Council on Companion Animal Parasites (ESCCAP), along with several other studies, has proposed varying deworming guidelines for dogs, recommending treatment intervals ranging from monthly, bimonthly, and every 3–4 months, to as infrequently as once per year. [16,20,30]. In-depth experimental field and modeling studies in different endemicity scenarios are required to establish the most effective deworming regimen in each endemic society.

Control programs often fail in rural settings due to limited veterinary infrastructure, poor compliance with deworming protocols, and traditional livestock husbandry, while limited community awareness of zoonotic risks reduces participation in control initiatives. A successful intervention program must integrate regular PZQ administration, livestock vaccination, regulation of offal disposal, and community education, supported by long-term political commitment and international cooperation. Without implementing these measures, long-term CE control efforts face serious challenges. Future studies should explore practical, cost-effective surveillance tools and dog vaccination strategies to break the cycle of dog re-infection in endemic countries [3,12].

Unregulated disposal of waste from home slaughtering practices significantly increases environmental contamination with *Echinococcus* eggs. Dogs in rural areas exhibit a 3.5- to 6.0-fold higher prevalence of CE compared to those in urban settings, reflecting closer contact with livestock and limited access to veterinary services and control measures [28]. Inadequate deworming heightens these risks, as untreated dogs exhibit higher infection odds than regularly dewormed counterparts [32]. Despite PZQ efficacy, logistical barriers including cost, accessibility, and community compliance, undermine sustained deworming in resource-limited regions. Diagnostic variability, e.g., coproantigen ELISA, copro-PCR and microscopy, may also affect accuracy.

The study underscores the challenges in controlling CE in endemic regions, where persistent *E. granulosus s. l.* reinfection in dogs undermines control efforts. The findings reveal a notably high rate of reinfection in dogs, largely driven by unregulated home slaughter that enables repeated access to infected offal. This highlights how husbandry practices, such as home slaughter and offal feeding, sustain parasite transmission cycles. These results call for urgent policy action, including stricter regulation of slaughter practices, strengthened responsible dog ownership, and targeted community-based education on zoonotic risks. The study also calls for adaptive control strategies tailored to regional transmission dynamics, including genotype-specific risks and geographic hotspots.

This study had several limitations that should be considered when interpreting the findings. A key limitation of our study is the potential misclassification between true re‑infection and incomplete clearance of the initial infection following PZQ treatment, causing residual infections to appear as new cases. Therefore, we acknowledge that some degree of re-infection misclassification bias cannot be entirely excluded in field studies of canine echinococcosis due to the limitations of available diagnostic tools. The definitive diagnosis of canine echinococcosis is complicated by the absence of a perfect gold standard diagnostic tool, necessitating careful consideration of each tool’s limitations. This underscores the importance of employing a combination of available methods, each with its own specific sensitivities and limitations, to achieve the most accurate assessment. However in our study, measures were implemented to minimize misclassification bias. The diagnostic method we used has high sensitivity and specificity, and we interpreted results cautiously to avoid overestimating re‑infection. Also, PZQ is reported to achieve >99% efficacy against *Echinococcus* spp. within 24–48 hours [38] and all PZQ treatments were administered directly by trained personnel under observation to ensure correct dosing and reduce the risk of incomplete treatment, thereby lowering the probability of treatment failure.

## Conclusion

This longitudinal study demonstrates the persistent challenge of *E. granulosus* s. l. reinfection in farm dogs despite PZQ treatment. The findings highlight the remarkable challenges in controlling CE in an endemic region, where more than one-fifth of the dogs were re-infected within one year, thereby undermining ongoing hydatid control efforts. The findings indicate that dog deworming is insufficient when environmental and behavioral risk factors remain unaddressed. In Iran little information is available in various endemic settings on echinococcosis re-infection in dogs and further studies are required to determine the appropriate treatment intervals, and cost estimates.

The study also calls for adaptive control strategies tailored to regional transmission dynamics, including genotype-specific risks and geographic hotspots. Future research should aim to perform comprehensive molecular characterization of all cases across different geographical endemic regions, particularly in areas dominated by various *E. granulosus* s.l. genotypes. Such comparative studies are essential to determine whether variations in local genotype distribution influence the likelihood or rate of re-infection. Large-scale multicenter investigations integrating molecular and epidemiological data would substantially improve our understanding of re-infection dynamics and help refine regional control strategies.

Breaking the reinfection cycle needs the integration of veterinary, public health, and community-based approaches, with policy support to ensure long-term feasibility. These insights are critical for advancing One Health strategies against CE in Iran and other endemic settings worldwide.

## Supporting information

S1 File Data file showing all owned dogs included in the 15-month follow-up study after praziquantel treatment, containing individual characteristics, diagnostic results at each sampling time point, and reinfection outcomes used for the analyses. (XLSX)

## Abbreviations

FRDs: Free-roaming dogs
CE: Cystic echinococcosis
nad1: NADH dehydrogenase subunit
AE: Alveolar echinococcosis
