# Supplementary material for: Reinfection dynamics of owned dogs with Echinococcus granulosus sensu lato in endemic rural areas of central Iran: Implications for cystic echinococcosis control
Source: PLoS Negl Trop Dis. 2026 Jun 29;20(6):e0014451. doi: 10.1371/journal.pntd.0014451 (PMC13327511; doi:10.1371/journal.pntd.0014451)
Supplement: Attachment — Submitted filename: Revision and recommendation final version.pdf [file pntd.0014451.s003.pdf]

Comments on the Relevance of the Study:

The submitted manuscript addresses a topic that remains underexplored and, in many respects, poorly understood. The main merit of the study lies in its contribution to advancing this field by providing new data and insights that enhance the current knowledge on the subject.

Recommendation:

A **minor revision** is recommended, as the manuscript shows potential to enrich the scientific debate and contribute to a better understanding of *Echinococcus granulosus* sensu lato reinfection in dogs from rural areas.

The study investigates reinfection by *Echinococcus granulosus* sensu lato in herding and guard dogs following treatment with praziquantel. Fecal samples from 381 dogs in rural areas of Iran were analyzed, with 31 samples testing positive for *Echinococcus* eggs. After chemotherapy, fecal samples from the same animals were collected at different time points to assess reinfection. The results indicate that the likelihood of reinfection increased with time after treatment. Furthermore, herding dogs exhibited the highest reinfection rates, along with various risk factors contributing to reinfection.

Through molecular biology analysis, the presence of *Echinococcus* sensu stricto and *Echinococcus canadensis* was confirmed in some samples. Therefore, this study is original and provides updated data and evidence that support efforts to control cystic echinococcosis in countries facing this zoonotic disease.

Comments and Questions:

1. One of the most important recommendations I would like to highlight is the standardization of the scientific name currently used for *Echinococcus granulosus*. When referring to all genetic variants of the species, the correct terminology is *Echinococcus granulosus* sensu lato (Vuitton et al., 2020). Throughout the manuscript, including the title, the use of this term is inconsistent. Sometimes the correct terminology is used, while at other times, only *Echinococcus granulosus* appears. I recommend a careful review of the nomenclature to ensure uniformity.

2. Regarding the methodology for collecting fecal samples from herding dogs: how can one be certain that the feces collected at the resting places truly belong to the intended dog, considering these animals are free to roam the property? A more detailed explanation of this procedure would be helpful.

3. Another relevant question relates to the samples analyzed by molecular biology. Out of the 31 samples that tested positive for *Echinococcus granulosus* sensu lato, why were only 9 sequenced? Wouldn't it be more

informative to sequence all samples to better identify the genotypes involved in the study? This is especially important considering that different genotypes may behave differently during the course of infection.

4. On page 4 (Introduction), third line: replace the word **“cardinal”** with **“main.”**
5. On page 4 (Introduction), line 20: please add a reference for the statement: *“Dogs can become...CE transmission.”*
6. On page 4 (Introduction), line 25: please add a reference for the statement: *“The frequency...endemic region.”*
7. On page 6 (Materials and Methods), line 7: the citation of **Wikipedia** as a reference is not appropriate in a scientific manuscript. Is there a more reliable bibliographic source that could be used instead? I suggest replacing this reference.
8. On page 7 (Materials and Methods), line 21: where it reads *“for identification of the dogs,”* I suggest rephrasing to: **“for identification of dogs during...”**
9. In the title of **Table 1**, I recommend rephrasing to: **“Study for the diagnosis of cystic canine echinococcosis...”**
