# Supplementary material for: Reinfection dynamics of owned dogs with Echinococcus granulosus sensu lato in endemic rural areas of central Iran: Implications for cystic echinococcosis control
Source: PLoS Negl Trop Dis. 2026 Jun 29;20(6):e0014451. doi: 10.1371/journal.pntd.0014451 (PMC13327511; doi:10.1371/journal.pntd.0014451)
Supplement: Attachment — Submitted filename: PNTD-D-25-01258_reviewer.pdf [file pntd.0014451.s005.pdf]

PLOS Neglected Tropical Diseases

Reinfection dynamics of owned dogs with Echinococcus granulosus in endemic rural areas of central Iran: Implications for cystic echinococcosis control

--Manuscript Draft--

**Manuscript Number:**	PNTD-D-25-01258
**Full Title:**	Reinfection dynamics of owned dogs with Echinococcus granulosus in endemic rural areas of central Iran: Implications for cystic echinococcosis control
**Short Title:**	Reinfection dynamics of the owned dogs with Echinococcus granulosus in Iran
**Article Type:**	Research Article
**Keywords:**	Canine echinococcosis; Anthelmintic treatment; Reinfection; Transmission dynamics
**Abstract:**	Background<br><br>Cystic echinococcosis (CE), caused by the tapeworm Echinococcus granulosus, is highly endemic in many regions of the world including Iran, with dogs as definitive hosts, playing a critical role in the dynamics of CE transmission. Regular deworming of dogs is an essential part of any successful CE control program. However, dogs can become easily reinfected after praziquantel (PZQ) administration, and the rate of reinfection of dogs in an endemic area is considered an indicator of the intensity of CE transmission. This study aimed to evaluate reinfection rate in the owned dogs after praziquantel treatment in Semnan province, in North of Iran.<br><br>Methods<br><br>A cohort of 381 guard and sheepdogs was monitored for 15 months post-treatment with praziquantel. Reinfection was assessed at 3, 6, 9, 12, and 15 months by fecal sampling. Samples were microscopically examined for taeniid eggs after formalin-ether sedimentation and were confirmed by PCR using E. granulosus sensu lato-specific primers. Kaplan-Meier survival analysis was used to calculate the cumulative survival rate over a 15-month period. A Cox hazard regression model was employed to identify risk factors associated with the time to reinfection with E. granulosus.<br><br>Results<br><br>Fecal samples from 151 sheepdogs and 230 guard dogs from 290 farms, were examined. At baseline, microscopic and copro-PCR analyses revealed 12.6% positive for taeniid eggs, of which 31 samples (8.1%) were positive for E. granulosus s. l. Re-infection rate was estimated as 9.6%, 10.7%, 15.6%, 22.5%, and 33.2% at 3, 6, 9, 12, and 15 months after initial treatment with praziquantel, respectively. Sheepdogs showed significantly higher reinfection risks (29.8%) compared to the guard dogs (16.7%). Dogs whose owners practiced home slaughter were 2.02 times more likely to become reinfected. Only 17.3% (66/381) of the dogs were reported to be regularly dewormed by the owners.<br><br>Conclusion<br><br>This study demonstrates that re-infection of dogs with E. granulosus is common in CE-endemic regions of Iran. The high rate of re-infection has significant implications for CE control, particularly in determining the most effective frequency for administering praziquantel to prevent sustained transmission.
**Additional Information:**	
**Question**	**Response**
**Government Employee**<br><br><br>Are you or any of the contributing authors	No - No authors are employees of the U.S. government.

Powered by Editorial Manager® and ProduXion Manager® from Aries Systems Corporation

an employee of the United States government?<br><br>Manuscripts authored by one or more US Government employees are not copyrighted, but are licensed under a CC0 Public Domain Dedication, which allows unlimited distribution and reuse of the article for any lawful purpose. This is a legal requirement for US Government employees.<br><br>This will be typeset if the manuscript is accepted for publication.	
**Financial Disclosure**<br><br>Enter a financial disclosure statement that describes the sources of funding for the work included in this submission. Review the submission guidelines for detailed requirements. View published research articles from *PLOS Neglected Tropical Diseases* for specific examples.<br><br>This statement is required for submission and **will appear in the published article** if the submission is accepted. Please make sure it is accurate.<br><br>**Funded studies**<br>Enter a statement with the following details:<br>• Initials of the authors who received each award<br>• Grant numbers awarded to each author<br>• The full name of each funder<br>• URL of each funder website<br>• Did the sponsors or funders play any role in the study design, data collection and analysis, decision to publish, or preparation of the manuscript?<br><br>Did you receive funding for this work?	Yes
Please add funding details.<br>as follow-up to "**Financial Disclosure**<br><br>Enter a financial disclosure statement that	The Vice Chancellor of Research and Technology of Hamadan University of Medical Sciences dedicated a grant for this research (Grant Number: 40799).

describes the sources of funding for the work included in this submission. Review the submission guidelines for detailed requirements. View published research articles from *PLOS Neglected Tropical Diseases* for specific examples.<br><br>This statement is required for submission and **will appear in the published article** if the submission is accepted. Please make sure it is accurate.<br><br>**Funded studies**<br>Enter a statement with the following details:<br>• Initials of the authors who received each award<br>• Grant numbers awarded to each author<br>• The full name of each funder<br>• URL of each funder website<br>• Did the sponsors or funders play any role in the study design, data collection and analysis, decision to publish, or preparation of the manuscript?<br><br>Did you receive funding for this work?"	
Please select the country of your main research funder (please select carefully as in some cases this is used in fee calculation).<br>as follow-up to "**Financial Disclosure**<br><br>Enter a financial disclosure statement that describes the sources of funding for the work included in this submission. Review the submission guidelines for detailed requirements. View published research articles from *PLOS Neglected Tropical Diseases* for specific examples.<br><br>This statement is required for submission and **will appear in the published article** if the submission is accepted. Please make sure it is accurate.	IRAN, ISLAMIC REPUBLIC OF - IR

**Funded studies**<br>Enter a statement with the following details:<br>• Initials of the authors who received each award<br>• Grant numbers awarded to each author<br>• The full name of each funder<br>• URL of each funder website<br>• Did the sponsors or funders play any role in the study design, data collection and analysis, decision to publish, or preparation of the manuscript?<br><br>Did you receive funding for this work?"	
**Competing Interests**<br><br>On behalf of all authors, disclose any competing interests that could be perceived to bias this work.<br><br>This statement will be typeset if the manuscript is accepted for publication.<br><br>Review the instructions link below and PLOS NTDs' competing interests policy to determine what information must be disclosed at submission.	None
**Data Availability**<br><br>Provide a **Data Availability Statement** in the box below. This statement should detail where the data used in this submission can be accessed. This statement will be typeset if the manuscript is accepted for publication.<br><br>Before publication, authors are required to make all data underlying their findings fully available, without restriction. Review our PLOS Data Policy page for detailed information on this policy. Instructions for writing your Data Availability statement can be accessed via the Instructions link below.	The data that support the findings of this study are available as Supporting Information file.

Main manuscript text Click here to access/download;Manuscript;SRM_manuscript_14040506.doc

Reinfection dynamics of owned dogs with *Echinococcus granulosus* in endemic rural areas of central Iran: Implications for cystic echinococcosis control

Seyed Reza Mirbadie [1, 2], Saeedeh Shamsaddini[3], Mohammad Taghi Rahimi[2], Mohammad Matini[1], Mohammad Ali Mohaghegh[4], Mohammad Reza Baneshi[5], Majid Fasihi Harandi[3*], Mohammad Fallah[1*]

1. Department of Medical Parasitology and Mycology, School of Medicine, Hamadan University of Medical Sciences, Hamadan, Iran

2. School of Medicine, Shahroud University of Medical Sciences, Shahroud, Iran

3. Research Center for Hydatid Disease in Iran, Institute of Infectious Diseases and Tropical Medicine, Afzalipour School of Medicine, Kerman University of Medical Sciences, Kerman, Iran

4. Department of Laboratory Sciences, School of Paramedical Sciences, Torbat Heydariyeh University of Medical Sciences, Torbat Heydariyeh, Iran.

5. The University of Queensland, Australian Women and Girls' Health Research Centre, School of Public Health, Herston, Queensland, Australia.

* Correspondences: majid.fasihi@gmail.com ; mohfall@yahoo.com

ABSTRACT

Background Cystic echinococcosis (CE), caused by the tapeworm *Echinococcus granulosus*, is highly endemic in many regions of the world including Iran, with dogs as definitive hosts, playing a critical role in the dynamics of CE transmission. Regular deworming of dogs is an essential part of any successful CE control program. However, dogs can become easily reinfected after praziquantel (PZQ) administration, and the rate of reinfection of dogs in an endemic area is considered an indicator of the intensity of CE transmission. This study aimed to evaluate reinfection rate in the owned dogs after praziquantel treatment in Semnan province, in North of Iran.

Methods: A cohort of 381 guard and sheepdogs was monitored for 15 months post-treatment with praziquantel. Reinfection was assessed at 3, 6, 9, 12, and 15 months by fecal sampling. Samples were microscopically examined for taeniid eggs after formalin-ether sedimentation and were confirmed by PCR using *E. granulosus* sensu lato-specific primers. Kaplan-Meier survival analysis was used to calculate the cumulative survival rate over a 15-month period. A Cox hazard regression model was employed to identify risk factors associated with the time to reinfection with *E. granulosus*.

Results: Fecal samples from 151 sheepdogs and 230 guard dogs from 290 farms, were examined. At baseline, microscopic and copro-PCR analyses revealed 12.6% positive for taeniid eggs, of which 31 samples (8.1%) were positive for *E. granulosus s. l*. Re-infection rate was estimated as 9.6%, 10.7%, 15.6%, 22.5%, and 33.2% at 3, 6, 9, 12, and 15 months after initial treatment with praziquantel, respectively. Sheepdogs showed significantly higher reinfection risks (29.8%) compared to the guard dogs (16.7%). Dogs whose owners practiced home slaughter were 2.02 times more likely to become reinfected. Only 17.3% (66/381) of the dogs were reported to be regularly dewormed by the owners.

Conclusion: This study demonstrates that re-infection of dogs with *E. granulosus* is common in CE-endemic regions of Iran. The high rate of re-infection has significant implications for CE control, particularly in determining the most effective frequency for administering praziquantel to prevent sustained transmission.

Keywords: Canine echinococcosis; Anthelmintic treatment; Reinfection; Transmission dynamics

INTRODUCTION

Cystic echinococcosis, caused by the small tapeworm of canids *Echinococcus granulosus* sensu lato, is a major parasitic zoonosis of humans in the world affecting livestock and dogs as the intermediate and definitive hosts, respectively. Currently, more than one million people worldwide are infected with cystic echinococcosis (CE), and this condition is considered a serious public health challenge [1, 2]. The impact of this disease is primarily on the marginalized and rural communities with low socioeconomic status and inadequate management and control over dogs.

Global reports of the incidence of cystic echinococcosis have estimated 207,368 new cases with 122,457 DALYs [3]. The surgical incidence of CE varies between 1 and 200 cases per 100,000 people worldwide. CE is endemic with high incidence in most Middle East and North African countries including Iran, where the incidence is estimated 0.61-1.27 per 100,000 [4]. Considering the close relationship between dogs and humans, dogs play a key role in CE transmission. The overall estimated prevalence of infection in dogs in Iran is 23.6% [5, 6]. Also a significant economic and social costs are imposed on CE-endemic societies, which include the costs of medical and surgical treatment and disability in humans, as well as damages to the

livestock industry [7, 8]. The overall annual monetary burden of CE in Iran has been estimated at US$232 million [9, 10].

The role of dogs as the cardinal definitive host and reservoir of the parasite is crucial in the spread of parasite eggs in the environment through canine feces. Guard dogs and sheepdogs are essential in the activity of the people living in the rural and semi-rural regions in Iran, where agriculture and animal husbandry is their major occupation [11, 12]. One of the key factors in CE transmission in most parts of the world is the close relationship of dogs, livestock, and humans. Studies indicate that the global dog population has surpassed 700 million, of which 75% are free-roaming. The ratio of dogs to humans ranges from 0.2 to >20 dogs per 100 people in different countries [13, 14, 15].

Necessary measures for CE control include livestock vaccination, regular dog deworming, dog registration, dog population management, improving abattoir infrastructure, controlling home slaughter and dog access to livestock offal [3, 12]. Echinococcosis in dogs is very difficult to control and to date, only a limited number of control programs in a few countries have been successful [16, 17]. Various studies have shown that regular deworming of dogs plays a substantial role in reducing the incidence of canine echinococcosis [12, 18, 19]. Dog deworming campaigns have been hampered by frequent reinfection of dogs following access to the infected livestock offal. Dogs can become easily reinfected after praziquantel (PZQ) administration, and the rate of reinfection of dogs in an endemic area is considered an indicator of the intensity of CE transmission.

Dog deworming campaigns have been challenged by major logistic and administrative problems related to the frequent site visits and anthelmintic treatment. The frequency of PZQ administration is correlated with the rate of dog reinfection in each endemic region. Knowledge about the nature and extent of reinfection in dogs is essential for any CE control program.

Reinfection studies are required to provide baseline information for control and prevention of CE in endemic areas. Studies conducted in two North African countries, Morocco and Tunisia, demonstrated that administering treatment to dogs at two-month intervals was more effective in controlling reinfection compared to less frequent regimens [11, 20, 21].

Very few reinfection studies have been conducted in Iran. In a recent study, reinfection of owned dogs reached 17% one year after initial PZQ treatment with a monthly reinfection incidence of 1.5% [22]. Obviously, further studies on dog reinfection are required to improve our understanding of the dynamics of CE transmission in different endemic regions as an essential requisite for accomplishing a successful CE control program.

This study aimed to assess and estimate the cumulative incidence of reinfection with *E. granulosus* in owned dogs and identify factors associated with increased risk of re-infection after treatment with PZQ.

MATERIALS AND METHODS

Ethics statement

The project was approved by the ethical review committee of Hamadan University of Medical Sciences (IR.UMSHA.REC.1401.877). The study objectives were fully explained to the dog owners, emphasizing the importance of the study. Prior to sampling, verbal consent was obtained from the owners of the dogs. With the owners' consent, all dogs present at each respective farm or herd were sampled, which were primarily working animals such as sheepdogs and guard dogs.

Study area

This cross-sectional study was conducted in Semnan Province, Iran. Semnan is located between the edge of the desert plain (central desert of Iran) and the Alborz Mountain range

with a high-altitude variation between 1000 and 3000 m above sea level. The province is located between latitude 34°13′-37°20′N and longitude 51°51′-57°3′E in north-central Iran with a surface area of 97,491 Km^2 and a population of 702,360. Semnan has a diverse climate with a temperate climate in the northern areas and arid and semi-arid regions in the south. The average annual rainfall is 139.5 mm. (https://www.worldweatheronline.com/semnan-weather-averages/semnan/ir.aspx) with 13.6 million acres of pasture and 2.5 million livestock (https://en.wikipedia.org/wiki/Semnan_Province).

Study design and Sample collection

In total, 290 farms from 101 villages were randomly selected from five counties in Semnan province, including Semnan, Shahroud, Damghan, Mayamey, and Garmsar. A total of 381 dogs were recruited in the study (Fig 1).

The living place of the dogs and their roaming status were recorded and sampling was done as follows. For those dogs that were tied up constantly or only during the day, the fresh feces samples were collected from inside and/or around their living place. For the free-roaming sheep dogs, the fecal samples were collected from around each dog's resting place with the help of the dog owner. The samples were placed in plastic containers in cold boxes and transferred promptly to the laboratory for parasitological examination. One part of the sample was preserved in 10% formalin for sedimentation test and microscopic examination and another for DNA extraction and molecular work. For safety reasons all samples were kept at -80°C for at least ten days.

Parasitological and Molecular analyses

All samples were microscopically examined for taeniid eggs following formalin-ether concentration technique. Subsequently, sugar flotation method was performed on the positive samples as described elsewhere. In the next step, taeniid eggs were collected and subjected to

molecular analysis. All dogs found infected with taeniid eggs, were treated with PZQ, 5 mg/kg of body weight. Seven days after anthelmintic treatment, re-sampling was done to check the outcome of the treatment. Re-treatment was performed until a negative result was obtained. Re-samplings were performed to investigate the re-infection rate of the dogs at 3, 6, 9, 12, and 15 months after the initial administration of PZQ.

For molecular analysis, PCR-sequencing was performed using three primer pairs amplifying partial cytochrome c oxidase (COX1) gene (444 bp), a 133-bp fragment of a tandem repeat sequence, and a partial SSU-rDNA region (149 bp). The cox1 primer pair was taeniid-specific and the other two primer sets were used for the specific detection of *E. granulosus sensu lato* [22, 23, 24]. Table 1 shows the primer sequences used in the present study. Previously sequenced DNA samples were used as positive controls [7].

For genotype identification, the PCR products obtained from *E. granulosus*-positive samples were randomly selected, purified and sent for Sanger sequencing (Pishgam co. Tehran) by targeting SSU-rDNA using the reverse primer (Table 1). The sequence data were edited and analyzed by Sequencher 4.7 and MEGA 5.05. The obtained sequences were examined using the NCBI BLAST database for the final identification and confirmation of *E. granulosus* genotypes.

Dog data collection

A data collection sheet was filled for each dog before fecal sampling. Data related to each dog were recorded including age, sex, location, anthelmintic treatment, type of dog food, and roaming habits. Specific dog characters were recorded for identification of the dogs during follow-up. In addition, dog owners were asked about home slaughter and disposal of sheep carcasses and infected organs.

Statistical analysis

The Kaplan-Meier method was used to calculate and visualize the cumulative survival rate stratified by age, sex, and type of dog. Cumulative incidence was defined as the complement of survival rates (i.e., one minus survival rates). Time was considered as the variable of infection time for dogs with reinfection, and the time of the last microscopic examination was defined as the censored time for uninfected dogs.

To identify risk factors associated with the time to reinfection with *E. granulosus*, a Cox hazards regression model was employed. Eight variables including gender, age, dog type, roaming behavior, feeding habits, anthelmintic treatment, location, and home slaughter were evaluated using Cox analysis. Adjusted Hazard Ratios (HRs) and their 95% confidence intervals (CIs) were estimated to quantify the magnitude of association between each covariate and the risk of reinfection. All statistical analyses were conducted using R software.

RESULTS

Study Population, Baseline Prevalence and genotyping

Fecal samples from a total of 381 owned dogs, including 151 sheepdogs and 230 guard dogs from 290 farms, were collected between February 2023 and May 2024 in different villages in five counties of Semnan province (Fig 1). Over an 18-month period, the dogs were evaluated for re-infection with *E. granulosus*. Microscopic study and copro-PCR analyses showed a total of 48 samples (12.6%) as positive for taeniid eggs before the start of the deworming program, of which 31 samples (8.1%) were positive for *E. granulosus s. l*. Taeniid eggs were detected in 20 guard dogs and 28 sheepdogs, among which *E. granulosus* was identified in 14 (6.1%) and 17 (11.2%) individuals, respectively (Table 2). Nine *E. granulosus*-positive samples were PCR-sequenced on a partial SSU-rDNA (~149 bp) and compared with the sequences in NCBI GenBank. Eight isolates were identified as *E. granulosus* sensu stricto (s.s.) (G1-G3) and one as *E. canadensis* G6-G7.

Reinfection Dynamics and Survival Analysis

Following initial treatment with praziquantel, the cohort of dogs was monitored for reinfection over a 15-month period. Table 3 presents the survival rates and cumulative incidence of infection at 3, 6, 9, 12, and 15 months post-treatment with praziquantel. The cumulative incidence of reinfection with *E. granulosus* progressively increased throughout the follow-up. At 3 months post-treatment, the cumulative incidence was 9.6% (95% CI: 6.5-12.5%). This rate rose to 10.7% (95% CI: 7.5-13.7%) at 6 months, 15.6% (95% CI: 11.9-19.1%) at 9 months, and 22.5% (95% CI: 18.0-26.8%) at the 12-month mark. By the end of the 15-month study period, the cumulative incidence of reinfection reached 33.2% (95% CI: 27.7-38.3%) (Table 3).

Kaplan-Meier survival analysis was used to visualize the time to reinfection (Figure 2). The analysis, when stratified by dog type, showed a significantly lower survival probability (i.e., a higher rate of reinfection) for sheepdogs compared to guard dogs over the 15-month period (Figure 2C). The final reinfection risk was 29.8% for sheepdogs versus 16.7% for guard dogs. No significant differences in survival rates were observed when dogs were stratified by sex or age (Figure 2A, 2B)

Risk Factor Analysis for Reinfection

A Cox proportional hazards regression model was employed to identify risk factors associated with the time to reinfection. Table 4 shows hazard ratios obtained from analyzing the potential risk of echinococcosis in the owned dogs in rural areas of Semnan province. The analysis revealed several significant predictors. Dog type was a primary risk factor, with sheepdogs having a 1.8 times higher risk of reinfection compared to guard dogs (Adjusted Hazard Ratio [HR]: 1.8, 95% CI: 1.22-2.68; $p<0.05$). Practices related to livestock slaughter and feeding were also strongly associated with reinfection. Dogs whose owners practiced

home slaughter were 2.02 times more likely to become reinfected (HR: 2.02, 95% CI: 1.35-3.03; $p<0.05$). Similarly, feeding dogs with raw livestock offal was identified as a significant risk factor ($p=0.001$).

Furthermore, the history of anthelmintic treatment was a significant factor. Dogs that were not regularly dewormed by their owners had a 1.76 times greater risk of reinfection than those receiving regular treatment (HR: 1.76, 95% CI: 1.04-2.97; $p<0.05$). Data collected from owners indicated that only 17.3% (66/381) of the dogs in the study were reported to be regularly dewormed prior to the study's intervention. Other variables, including the dog's gender, age, roaming behavior, and location, were not found to be significantly associated with the risk of reinfection in the final model (Table 4).

DISCUSSION

Echinococcosis is a major parasitic zoonosis and has been classified by the World Health Organization as a Neglected Tropical Disease (NTD), with control efforts prioritized under the 2030 global roadmap [25]. The disease is endemic in Iran, and domestic dogs are the main source of human infection. The prevalence of *E. granulosus* in dogs has been reported to range from 6.8% to 55.7% in different regions of Iran. According to recent studies, Semnan province in central Iran is one of the important foci of the CE in the country, with a prevalence of 7.4% in dogs, 13.3% in livestock, and 8.6% seroprevalence in humans [4, 26].

Regular dog deworming by the oral administration of PZQ at scheduled intervals is the first option in implementing CE control programs in endemic regions. However, re-infection of dogs after treatment is a major challenge in CE control programs. It has been shown that lack of treatment or inadequate treatment intervals increase the odds of reinfection by 23.3-52.5 [18].

To assess the re-infection rate of echinococcosis in dogs, a 15-month follow-up study was conducted at 3, 6, 9, 12, and 15 months after treatment with praziquantel. The rate of reinfection in dogs after treatment increased over time, such that 9.6% (6.5-12.5%) of dogs were reinfected with *E. granulosus*, three months after the initial treatment. The infection rate reached 22.5% (18.0-26.8%) one year after initial deworming (Table 3). High reinfection of farm dogs occurs following home slaughter and/or feeding offal to the owned dogs. Consequently, regular and frequent dog deworming is required to overcome the infection pressure in dogs. This presents major logistic and financial challenges for implementing effective CE control programs in endemic areas, as the frequency of PZQ dosing is related to dog reinfection pressure.

The rate of reinfection in owned dogs, particularly guard and sheepdogs, has been investigated in few studies worldwide, including Iran. Table 5 shows the reinfection studies conducted in different endemic countries. Findings of these studies indicate that in various endemic countries the reinfection rate ranges from 1.56% to 27.9% one year after initial treatment. A study in Libya estimated reinfection in 334 owned dogs. The rate of reinfection 15 months after treatment with PZQ reported to be 22.2% [27]. In Peru it was shown that failure of regular deworming of dogs increases the rate of reinfection [28]. In Uruguay, reinfection in dogs occurred after 2 to 4 weeks [17]. In the two studies conducted in Morocco and Iran, the monthly reinfection rates in domestic dogs were reported to be 4% and 1.5%, respectively [12, 22].

One single study was conducted in Iran to investigate reinfection in farm dogs in Kerman, and the reinfection rate one year after the study was 17%. In the present study 22.5% of the dogs were reinfected after one year and 33.2% after 15 months. Interestingly, higher reinfection was observed in the sheep dogs (29.8%) comparing to the guard dogs (16.7%). This is consistent with the findings of other studies conducted in Libya and Kazakhstan. In

Libya, herd dogs are 9 times more likely to be copro-Ag positive than domestic dogs [27, 29]. Also, findings of another study in Morocco indicate that the reinfection rate in stray dogs was higher than in owned dogs [20]. The higher chance of reinfection in the sheepdogs can be due to a higher chance of feeding with offal, free-roaming behavior, and home slaughter.

Variations in reinfection rates across different endemic regions may be attributed to differences in animal husbandry and slaughter practices, cyst fecundity, frequency of dog deworming, diversity of intermediate hosts, genotype-related factors, as well as socioeconomic, cultural, and lifestyle differences. Additionally, the use of different diagnostic methods may further contribute to these discrepancies. [30, 31].

Repeated re-infection of farm dogs with *E. granulosus* poses significant challenges for CE control. As definitive hosts, farm dogs in the CE-endemic regions, are particularly vulnerable to re-infection due to their frequent exposure to infected offal from home-slaughtered livestock, scavenging on carcasses, and inadequate access to regular anthelmintic treatment. The frequent re-infections undermine control efforts, as even treated dogs become reinfected when traditional or existing animal husbandry, slaughtering, and dog-keeping behaviors remain unchanged [17, 22, 31, 32]. Moreover, the persistent contamination of pastures, water sources, and farm environments with *Echinococcus* eggs sustains parasite circulation and increase the risk of human infection through accidental ingestion of eggs.

The implementation of regular canine deworming programs as a strategy for CE control faces significant operational and biological challenges that undermine effective CE control measures. The short prepatent period in canine echinococcosis (5-6 weeks) imply monthly PZQ administration to prevent egg shedding, however, this frequency is logistically demanding and economically unsustainable in endemic communities. Many endemic areas lack adequate veterinary infrastructure, resulting in inconsistent drug distribution and

treatment coverage gaps. The European Scientific Council on Companion Animal Parasites (ESCCAP), along with several other studies, has proposed varying deworming guidelines for dogs, recommending treatment intervals ranging from monthly, bimonthly, and every 3 to 4 months, to as infrequently as once per year. [18, 20, 30] . In-depth experimental field and modeling studies in different endemicity scenarios are required to establish the most effective deworming regimen in each endemic society.

Control programs often fail in rural settings due to limited veterinary infrastructure, poor compliance with deworming protocols, and traditional livestock husbandry, while limited community awareness of zoonotic risks reduces participation in control initiatives. A successful intervention program must integrate regular PZQ administration, livestock vaccination, regulation of offal disposal, and community education, supported by long-term political commitment and international cooperation. Without implementing these measures, long-term CE control efforts face serious challenges. Future studies should explore practical, cost-effective surveillance tools and dog vaccination strategies to break the cycle of dog re-infection in endemic countries [3, 12]. In addition, while the study focused on owned dogs, the presence of unowned dog populations in the same areas, were not quantified. Free-roaming dog populations frequently evade treatment efforts, while wildlife reservoirs in some regions contribute to environmental contamination. Further studies are required to investigate the nature and extent of reinfection in unowned free-roaming dogs in the endemic regions.

Due to the lack of longitudinal survival studies, Cox analyses and hazard ratios (HRs) are rarely reported in the studies to understand the factors associated with echinococcosis in dogs. However, risk ratios (RRs) and/or odds ratios (ORs) are frequently used in epidemiological studies to quantify associations between risk factors and canine echinococcosis. In the present study the dog type (guard vs. sheepdog), feeding dogs with livestock viscera, anthelmintic treatment of dogs, and home slaughter practice were

significantly associated with the risk of *Echinococcus* infection. Several studies have identified dog-keeping practices, and access to raw livestock offal as the predominant risk factors of canine CE [18, 33]. Dogs fed uncooked viscera in Peru exhibit 2.9 times (95% CI, 1.0–8. Also, in Uganda access of dogs to livestock slaughter facilities significantly increased the risk of canine echinococcosis (OR = 11) [34]. Allowing dogs to roam freely (OR=3.17), feeding dogs with viscera (OR=3.04), and slaughter at home (OR=3.53) demonstrate greater odds of *E. granulosus* infection [35]. This practice significantly sustains parasite lifecycles in the rural environments. Roaming behavior further elevates risk of canine echinococcosis in several studies.

In Libya non-restraint of dogs, home slaughtering of livestock, limited awareness and failure of owners to administer regular deworming treatments, were found as significant risk factors of infection [27]. The results of our study also showed that dogs that were constantly free-roamed accounted for more than half of the positive cases. However, in the present study this was not significantly associated with infection in dogs. It is important to note that dogs kept under constant restraint are very likely to be fed infected offal by their owners.

Our results showed that dogs whose owners practiced home slaughter were 2.02 times more likely to be re-infected with *E. granulosus*. In pastoral and rural communities, home slaughter bypasses veterinary meat inspection, allowing infected organs to be discarded or fed to dogs, with many studies reporting higher odds of canine infection in such settings [32, 34, 35]. This perpetuates egg contamination of rural environments, increasing risk of ingesting *Echinococcus* eggs by humans. Consequently, home slaughter undermines abattoir regulations and CE control programs.

The results of our questionnaire survey showed that only 39.3% of dog owners had sufficient information about the life cycle and routes of infection in dogs. In a study in rural Peru, more

than 23% of dog owners fed offal to their dogs. A study in Libya found that home slaughter increased the risk of copro-antigen positivity of dogs by 8 times. Various studies consider the lack of awareness in dog owners and low knowledge of CE transmission as the main factors of infection in shepherd and domestic dogs [27, 28]. Also, socio-cultural traditions and economic factors are important in resource-limited regions. Interventions targeting this practice through community education, subsidized slaughter facilities, and incentivized suitable offal disposal, are critical to disrupt CE transmission. Without addressing home slaughter, sustained reductions in canine and human echinococcosis remain unachievable [36].

Unregulated disposal of waste from home slaughtering practices significantly increases environmental contamination with Echinococcus eggs. Dogs in rural areas exhibit a 3.5- to 6.0-fold higher prevalence of cystic echinococcosis compared to those in urban settings, reflecting closer contact with livestock and limited access to veterinary services and control measures [29]. Inadequate deworming heightens these risks, as untreated dogs exhibit higher infection odds than regularly dewormed counterparts [33]. Despite PZQ efficacy, logistical barriers including cost, accessibility, and community compliance, undermine sustained deworming in resource-limited regions. Diagnostic variability e.g., coproantigen ELISA, copro-PCR and microscopy, may also affect accuracy.

According to previous studies, the genotype of the parasite is another important factor influencing the effectiveness of deworming interventions in dogs [37]. Our findings indicated that *E. granulosus* s.s. (G1/G3) and *E. canadensis* genotypes were present in dogs, in line with previous studies in other endemic regions of the country [38]. In Semnan Province, genotypes G1, G3, G6, and G7 of *E. granulosus* have been identified in both stray and owned dogs, as well as in livestock isolates [26, 39]. The pre-patent period of *E. granulosus* sensu lato in dogs shows variation across different genotypes, particularly between *E. granulosus*

sensu stricto (G1–G3) and *E. canadensis* (G6–G7). In experimental infections, G1 genotype exhibits a relatively short pre-patent period with adult worms becoming mature and shedding eggs within 6 weeks post-infection. In contrast, infections with the G6/G7 genotypes, show a longer pre-patent period, ranging from 45 to 60 days. This presents important implications for epidemiological surveillance, transmission dynamics and hydatid control strategies, with a significant effect on the timing of deworming interventions in regions where G1/G3 or G6/G7 genotypes are dominant [17].

CONCLUSION

This longitudinal study demonstrates the persistent challenge of *E. granulosus* reinfection in farm dogs despite PZQ treatment. The study underscores the challenges in controlling CE in endemic regions, where persistent *E. granulosus* reinfection in dogs undermines control efforts. The high reinfection rates observed highlight how husbandry practices, such as home slaughter and offal feeding, sustain parasite transmission cycles. The findings underscore how unregulated home slaughter perpetuates transmission by enabling access to contaminated offal. Critically, the findings reveal that dog deworming are insufficient when environmental and behavioral risk factors remain unaddressed, emphasizing the need for holistic interventions requiring fundamental shifts in local slaughter practices, dog-keeping behaviors, and zoonotic awareness. In Iran little information is available on echinococcosis re-infection in dogs and further studies are required on reinfection in dogs to determine the appropriate treatment intervals, and cost estimates.

The study also calls for adaptive control strategies tailored to regional transmission dynamics, including genotype-specific risks and geographic hotspots. Breaking the reinfection cycle needs the integration of veterinary, public health, and community-based approaches, with

policy support to ensure long-term feasibility. These insights are critical for advancing One Health strategies against CE in Iran and other endemic settings worldwide.

Abbreviations:

FRDs: Free-roaming dogs

CE: Cystic echinococcosis

nad1: NADH dehydrogenase subunit

AE: Alveolar echinococcosis

Acknowledgements

The authors would like to thank the Vice-Chancellor of Research and Technology of Hamadan University of Medical Sciences for approving and providing financial support for this study. The authors also thank Shahroud University of Medical Sciences for providing its facilities to the project.

Author contributions

Conceptualization and study design: MFH, MF, SRM, SS

Data collection/curation: SRM

Performed the experiments and data analysis: SRM, MFH, SS, MF

Investigation: SRM, MF, MFH, SS

Project supervision: MF, MFH, MM

Writing original draft preparation: SRM, MF, MFH, SS, MAM, MTR

Review and editing the final draft of the manuscript: SRM, SS, MTR, MM, MAM, MFH, MF. The submitted version is approved by all authors.

Funding

The Vice Chancellor of Research and Technology of Hamadan University of Medical Sciences dedicated a grant for this research (Grant Number: 40799).

Availability of data and materials

The data that support the findings of this study are available from the corresponding author upon reasonable request.

Declarations

Ethics approval and consent to participate

This study was approved by the Ethics Review Committee of Hamadan University of Medical Sciences (Ethical Code: IR.UMSHA.REC.1401.877)

Consent for publication

Not applicable.

Competing interest

The authors declare no competing interests.

Author details

1 Department of Medical Parasitology and Mycology, School of Medicine, Hamadan University of Medical Sciences, Hamadan, Iran

2 School of Medicine, Shahroud University of Medical Sciences, Shahroud, Iran

3 Research Center for Hydatid Disease in Iran, Institute of Infectious Diseases and Tropical Medicine, Afzalipour School of Medicine, Kerman University of Medical Sciences, Kerman, Iran

4 Department of Laboratory Sciences, School of Paramedical Sciences, Torbat Heydariyeh University of Medical Sciences, Torbat Heydariyeh, Iran.

5. The University of Queensland, Australian Women and Girls' Health Research Centre, School of Public Health, Herston, Queensland, Australia.

Authors' emails:

SRM: sedreza.mirbadie@gmail.com

SS: s.shamsadini91@gmail.com

MTR: rahimimt@gmail.com

MM: matini.mohammad@yahoo.com

MAM: mohaghegh1982@yahoo.com

MRB: m.baneshi@uq.edu.au

MFH: majid.fasihi@gmail.com

MF: mohfall@yahoo.com

References:

1. Aghajanzadeh M, Safarpoor F, Amani H, Alavi A. One-stage procedure for lung and liver hydatid cysts. Asian Cardiovasc Thorac Ann. 2008;16 5:392–5; doi: 10.1177/021849230801600510.
2. Mohaghegh MA, Yousofi-Darani H, Jafarian AH, Mirbadie SR, Fasihi-Harandi M, Ghavimi R, et al. Isolated Human and Livestock *Echinococcus granulosus* Genotypes Using Real-Time PCR of cox1 Gene in Northeast Iran. Acta Parasitol. 2019;64 3:679–85; doi: 10.2478/s11686-019-00117-w.
3. Yang Z, Liu K, Wen B, Fu T, Qin X, Li R, et al. Changes in the global epidemiological characteristics of cystic echinococcosis over the past 30 years and projections for the next decade: Findings from the Global Burden of Disease Study 2019. J Glob Health. 2024;14:04056; doi: 10.7189/jogh.14.04056.
4. Borhani M, Fathi S, Darabi E, Jalousian F, Simsek S, Ahmed H, et al. Echinococcoses in Iran, Turkey, and Pakistan: Old Diseases in the New Millennium. Clin Microbiol Rev. 2021;34 3:e0029020; doi: 10.1128/cmr.00290-20.
5. Khalkhali HR, Foroutan M, Khademvatan S, Majidiani H, Aryamand S, Khezri P, et al. Prevalence of cystic echinococcosis in Iran: a systematic review and meta-analysis. J Helminthol. 2018;92 3:260–8; doi: 10.1017/s0022149x17000463.
6. Mahmoudi S, Mamishi S, Banar M, Pourakbari B, Keshavarz H. Epidemiology of echinococcosis in Iran: a systematic review and meta-analysis. BMC Infect Dis. 2019;19 1:929; doi: 10.1186/s12879-019-4458-5.
7. Mirbadie SR, Najafi Nasab A, Mohaghegh MA, Norouzi P, Mirzaii M, Spotin A. Molecular phylodiagnosis of *Echinococcus granulosus* sensu lato and *Taenia hydatigena* determined by mitochondrial Cox1 and SSU-rDNA markers in Iranian dogs: Indicating the first record of pig strain (G7) in definitive host in the Middle East. Comp Immunol Microbiol Infect Dis. 2019;65:88–95; doi: 10.1016/j.cimid.2019.05.005.
8. Mirbadie S-R, Zivdari M, Kalani H, Vafaei M-R, Izadi S, Jabalameli Z, et al. Molecular identification of Echinococcus granulosus sensu lato by mitochondrial COX1 and SSU-rDNA markers in dogs in the west of Iran. Gene Reports. 2020;19:100616.
9. Barati L, Shahkar L, Raeiji A, Veghari G. Cystic hydatid disease in children in Gorgan from 2014 to 2021. Iran J Pediatr. 2023;33 5:e136687; doi: 10.5812/ijp-136687.
10. Fasihi Harandi M, Budke CM, Rostami S. The monetary burden of cystic echinococcosis in Iran. PLoS Negl Trop Dis. 2012;6 11:e1915; doi: 10.1371/journal.pntd.0001915.
11. Lahmar S, Sarciron ME, Rouiss M, Mensi M. *Echinococcus granulosus* and other intestinal helminths in semi-stray dogs in Tunisia: infection and re-infection rates. Tunis Med. 2008;86 7:657–64.
12. Amarir F, Rhalem A, Sadak A, Raes M, Oukessou M, Saadi A, et al. Control of cystic echinococcosis in the Middle Atlas, Morocco: Field evaluation of the EG95 vaccine in sheep and cesticide treatment in dogs. PLoS Negl Trop Dis. 2021;15 3:e0009253; doi: 10.1371/journal.pntd.0009253.
13. Shamsaddini S, Ahmadi Gohari M, Kamyabi H, Nasibi S, Derakhshani A, Mohammadi MA, et al. Dynamic modeling of female neutering interventions for free-roaming dog population management in an urban setting of southeastern Iran. Sci Rep. 2022;12 1:4781; doi: 10.1038/s41598-022-08697-w.
14. Belsare A, Vanak AT. Modelling the challenges of managing free-ranging dog populations. Sci Rep. 2020;10 1:18874; doi: 10.1038/s41598-020-75828-6.
15. Smith LM, Hartmann S, Munteanu AM, Dalla Villa P, Quinnell RJ, Collins LM. The Effectiveness of Dog Population Management: A Systematic Review. Animals (Basel). 2019;9 12; doi: 10.3390/ani9121020.

16. Inoue T, Nonaka N, Kanai Y, Iwaki T, Kamiya M, Oku Y. The use of tetracycline in anthelmintic baits to assess baiting rate and drug efficacy against *Echinococcus multilocularis* in foxes. Vet Parasitol. 2007;150 1-2:88–96; doi: 10.1016/j.vetpar.2007.08.027.
17. Cabrera PA, Lloyd S, Haran G, Pineyro L, Parietti S, Gemmell MA, et al. Control of *Echinococcus granulosus* in Uruguay: evaluation of different treatment intervals for dogs. Vet Parasitol. 2002;103 4:333–40; doi: 10.1016/s0304-4017(01)00603-3.
18. Weng X, Mu Z, Wei X, Wang X, Zuo Q, Ma S, et al. The effects of dog management on *Echinococcus* spp. prevalence in villages on the eastern Tibetan Plateau, China. Parasit Vectors. 2020;13 1:207; doi: 10.1186/s13071-020-04082-6.
19. Vienažindienė Ž, Joekel DE, Schaper R, Deplazes P, Šarkūnas M. Longitudinal study for anthelmintic efficacy against intestinal helminths in naturally exposed Lithuanian village dogs: critical analysis of feasibility and limitations. Parasitol Res. 2018;117 5:1581–90; doi: 10.1007/s00436-018-5843-5.
20. Amarir FE, Saadi A, Marcotty T, Rhalem A, Oukessou M, Sahibi H, et al. Cystic echinococcosis in three locations in the middle atlas, Morocco: Estimation of the infection rate in the dog reservoir. Vector Borne Zoonotic Dis. 2020;20 6:436–43; doi: 10.1089/vbz.2019.2538.
21. Yang SJ, Xiao N, Li JZ, Feng Y, Ma JY, Quzhen GS, et al. Smart deworming collar: A novel tool for reducing *Echinococcus* infection in dogs. PLoS Negl Trop Dis. 2021;15 7:e0009443; doi: 10.1371/journal.pntd.0009443.
22. Borhani M, Mohammadi MA, Entezami M, Baneshi MR, Nasibi S, Prada JM, et al. Reinfection of farm dogs following praziquantel treatment in an endemic region of cystic echinococcosis in southeastern Iran. PLoS Negl Trop Dis. 2024;18 3:e0011939; doi: 10.1371/journal.pntd.0011939.
23. Abbasi I, Branzburg A, Campos-Ponce M, Abdel Hafez SK, Raoul F, Craig PS, et al. Copro-diagnosis of *Echinococcus granulosus* infection in dogs by amplification of a newly identified repeated DNA sequence. Am J Trop Med Hyg. 2003;69 3:324–30.
24. Laurimaa L, Davison J, Plumer L, Süld K, Oja R, Moks E, et al. Noninvasive detection of *Echinococcus multilocularis* tapeworm in urban area, Estonia. Emerg Infect Dis. 2015;21 1:163–4; doi: 10.3201/eid2101.140136.
25. Malecela MN, Ducker C. A road map for neglected tropical diseases 2021-2030. Trans R Soc Trop Med Hyg. 2021;115 2:121–3; doi: 10.1093/trstmh/trab002.
26. Galeh TM, Spotin A, Mahami-Oskouei M, Carmena D, Rahimi MT, Barac A, et al. The seroprevalence rate and population genetic structure of human cystic echinococcosis in the Middle East: A systematic review and meta-analysis. Int J Surg. 2018;51:39–48; doi: 10.1016/j.ijsu.2018.01.025.
27. Buishi IE, Njoroge EM, Bouamra O, Craig PS. Canine echinococcosis in northwest Libya: assessment of coproantigen ELISA, and a survey of infection with analysis of risk-factors. Vet Parasitol. 2005;130 3-4:223–32; doi: 10.1016/j.vetpar.2005.03.004.
28. Moro PL, Lopera L, Bonifacio N, Gonzales A, Gilman RH, Moro MH. Risk factors for canine echinococcosis in an endemic area of Peru. Vet Parasitol. 2005;130 1-2:99–104; doi: 10.1016/j.vetpar.2005.03.015.
29. Torgerson PR, Heath DD. Transmission dynamics and control options for *Echinococcus granulosus*. Parasitology. 2003;127 Suppl:S143–58; doi: 10.1017/s0031182003003810.
30. Cabrera PA, Parietti S, Haran G, Benavidez U, Lloyd S, Perera G, et al. Rates of reinfection with E*chinococcus granulosus*, *Taenia hydatigena*, *Taenia ovis* and other cestodes in a rural dog population in Uruguay. Int J Parasitol. 1996;26 1:79–83; doi: 10.1016/0020-7519(95)00082-8.
31. Moss JE, Chen X, Li T, Qiu J, Wang Q, Giraudoux P, et al. Reinfection studies of canine echinococcosis and role of dogs in transmission of *Echinococcus multilocularis* in Tibetan communities, Sichuan, China. Parasitology. 2013;140 13:1685–92; doi: 10.1017/s0031182013001200.

32. Wang YH, Rogan MT, Vuitton DA, Wen H, Bartholomot B, Macpherson CN, et al. Cystic echinococcosis in semi-nomadic pastoral communities in north-west China. Trans R Soc Trop Med Hyg. 2001;95 2:153–8; doi: 10.1016/s0035-9203(01)90142-7.
33. Craig P, Mastin A, van Kesteren F, Boufana B. *Echinococcus granulosus*: Epidemiology and state-of-the-art of diagnostics in animals. Vet Parasitol. 2015;213 3-4:132–48; doi: 10.1016/j.vetpar.2015.07.028.
34. Oba P, Ejobi F, Omadang L, Chamai M, Okwi AL, Othieno E, et al. Prevalence and risk factors of *Echinococcus granulosus* infection in dogs in Moroto and Bukedea districts in Uganda. Trop Anim Health Prod. 2016;48 2:249–54; doi: 10.1007/s11250-015-0943-z.
35. Yuan R, Wu H, Zeng H, Liu P, Xu Q, Gao L, et al. Prevalence of and risk factors for cystic echinococcosis among herding families in five provinces in western China: a cross-sectional study. Oncotarget. 2017;8 53:91568–76; doi: 10.18632/oncotarget.21229.
36. Yang YR, McManus DP, Huang Y, Heath DD. *Echinococcus granulosus* infection and options for control of cystic echinococcosis in Tibetan communities of Western Sichuan Province, China. PLoS Negl Trop Dis. 2009;3 4:e426; doi: 10.1371/journal.pntd.0000426.
37. Lashkarizadeh MR, Hooshmand N, Nasibi S, Mohammadi MA, Shamsaddini S, Kamyabi H, et al. Genetic Profile of Hydatid Cysts in Patients with Multi-Organ Involvement: Mixed Infections by Different Strains. Vector Borne Zoonotic Dis. 2019;19 10:724–30; doi: 10.1089/vbz.2018.2427.
38. Mirbadie SR, Kamyabi H, Mohammadi MA, Shamsaddini S, Harandi MF. Copro-PCR prevalence of *Echinococcus granulosus* infection in dogs in Kerman, south-eastern Iran. J Helminthol. 2018;92 1:17–21; doi: 10.1017/s0022149x17000074.
39. Mirbadie SR, Nasab AN, Mohaghegh MA, Norouzi P, Mirzaii M, Spotin A. Molecular phylodiagnosis of Echinococcus granulosus sensu lato and Taenia hydatigena determined by mitochondrial Cox1 and SSU-rDNA markers in Iranian dogs: indicating the first record of pig strain (G7) in definitive host in the Middle East. Comparative Immunology, Microbiology and Infectious Diseases. 2019;65:88–95.
40. Bowles J, Blair D, McManus DP. Genetic variants within the genus *Echinococcus* identified by mitochondrial DNA sequencing. Mol Biochem Parasitol. 1992;54 2:165–73; doi: 10.1016/0166-6851(92)90109-w.
41. Wang Q, Yu WJ, Zhong B, Shang JY, Huang L, Mastin A, et al. Seasonal pattern of *Echinococcus* re-infection in owned dogs in Tibetan communities of Sichuan, China and its implications for control. Infect Dis Poverty. 2016;5 1:60; doi: 10.1186/s40249-016-0155-4.
42. Lahmar S, Lahmar S, Boufana B, Bradshaw H, Craig PS. Screening for *Echinococcus granulosus* in dogs: Comparison between arecoline purgation, coproELISA and coproPCR with necropsy in pre-patent infections. Vet Parasitol. 2007;144 3-4:287–92; doi: 10.1016/j.vetpar.2006.10.016.

Table 1. The primer sequences used in the present study for diagnosing canine echinococcosis in Semnan province, Iran.

Primer	Name	Sequence (5´> 3´)	Length	Genetic marker	Reference
Forward (F)<br>Reverse (R)	JB3<br>JB4.5	TTTTTTGGGCATCCTGAGGTTTAT<br>TAAAGAAAGAACATAATGAAAATG	24<br>24	Cox1	[40]
Forward (F)<br>Reverse (R)	Eg1121a<br>Eg1122a	GAATGCAAGCAGCAGATG<br>GAGATGAGTGAGAAGGAGTG	18<br>20	133-bp tandem repeat unit	[23]
Forward (F)<br>Reverse (R)	EGfor1<br>EGrev1	GTGTGTTACATTAATAAGGGTG<br>CTTGTTACGACTTACCTCAA	22<br>20	SSU-rDNA	[24]

Table 2. The frequency of Taeniidae / *Echinococcus granulosus* eggs in the owned dogs of Semnan province, Iran, before anthelmintic treatment, using microscopy and copro-PCR techniques.

Study areas		No. of fecal samples examined	Type of dogs		Microscopic observation	Copro-PCR
			Sheepdog	Guard dog	No. of positive samples, taeniidae eggs (%)	No. of positive samples, E. granulosus sensu lato (%)
Central Iran	Mayamey	43	18	25	7 (16.3%)	4 (9.3%)
	Shahrood	119	46	73	14 (11.8%)	9 (7.5%)
	Damghan	97	38	59	11 (11.3%)	7 (7.2%)
	Semnan	64	24	40	6 (9.4%)	4 (6.2%)
	Garmsar	58	25	33	10 (17.2%)	7 (12%)
	**Total**	**381**	**151**	**230**	**48 (12.6%)**	**31 (8.1%)**

Table 3: Survival rate estimates among dogs reinfected with *Echinococcus granulosus* in Semnan province, Central Iran.

Time post-treatment (month)	All dogs, % (95% CI)	Guard dogs, % (95% CI)	Sheepdogs, % (95% CI)
**3**	90.4 (87.5, 93.5)	92.0 (88.5, 95.7)	88.3 (83.5, 93.4)
**6**	89.3 (86.2, 92.5)	91.5 (87.8, 95.4)	86.4 (81.3, 91.8)
**9**	84.4 (80.8, 88.2)	88.4 (84.1, 92.9)	79.4 (73.3, 85.9)
**12**	77.5 (73.2, 82.0)	83.3 (78.2, 88.7)	70.2 (63.3, 77.9)
**15**	66.8 (61.6, 72.3)	75.4 (69.2, 82.2)	55.8 (47.9, 65.0)

Table 4: Cox hazards regression analysis to assess the risk of echinococcosis reinfection in the owned dogs in rural areas of Central Iran.

Variable	Category	Hazard Ratio (HR)	95% CI	P-value
**Gender**	Female	Reference		
	Male	1.47	0.76, 2.85	0.25
**Age**	Adult	Reference		
	Pup / Young	0.94	0.55, 1.61	0.82
**Dog type**	Guard dog	Reference		
	Sheepdog	1.81	1.22, 2.68	0.003
**Roaming behaviour**	Always leashed	Reference		
	Free at Night	1.00	0.57, 1.74	0.99
	Always free	0.85	0.52, 1.38	0.51
**Feeding habit**	Human food	Reference		
	Viscera	2.01	1.30, 3.08	0.001
**Anthelmintic treatment**	No	Reference		
	Yes	0.50	0.27, 0.96	0.03
**Study area**	Shahroud	Reference		
	Mayamey	1.37	0.73, 2.61	0.32

	Damghan	0.99	0.58, 1.69	0.99
	Semnan	0.92	0.50, 1.69	0.79
	Garmsar	1.45	0.82, 2.55	0.19
**Home slaughter**	No	Reference		
	Yes	2.02	1.28, 3.18	0.002

Table 5. Summary of the findings of reinfection studies on canine echinococcosis conducted in different endemic countries.

Country	No. samples	Host	Months post treatment, (reinfection rate, %)	Method	Reference
**Iran**	381	owned dogs	3 (9.6%)<br>6 (10.7%)<br>9 (15.6%)<br>12 (22.5%)<br>15 (33.2%)	Copro-PCR	Present study
**Iran**	150	owned dogs	2 (8)<br>5 (12)<br>12 (17)	Copro-PCR	[22]
**Morocco**	595	owned dogs	monthly rate 4%	Copro-PCR	[12]
**China (Shiqu county)**	584	owned dogs	3 (13.36)<br>6 (5.8)<br>9 (0)<br>12(1.56)	Copro-ELISA	[41]
**China (Seda county)**	584	owned dogs	3 (12.5)<br>6 (3.13)<br>9 (0)<br>12(1.56)	Copro-ELISA	[41]
**China**	281	owned dogs	2 (9.6)<br>5 (3.1)<br>12 (11)	Copro-PCR<br>Copro-ELISA	[31]
**Tunisia**	375	semi-stray dogs	2 (6.45)<br>4 (3.57)<br>8 (6.25)<br>12 (16.66)	Flotation	[42]
**Libya**	334	owned dogs	15 (22.2)	Copro-antigen	[27]

**China**	50	owned dogs	3 (25)<br>7 (29.2)<br>12 (16.7)	Copro-antigen	[32]
**Uruguay**	367	ND	2 (0)<br>4 (6.8)<br>8 (18.6)<br>12(27.9)	ND	[30]

ND: No data

Figure 1. Distribution of the sampled farms in Semnan Province, Iran, showing 290 farms to assess *Echinococcus granulosus* reinfection in 381 dogs.

Figure 2. Kaplan-Meier Survival rate of the owned dogs reinfected with *Echinococcus granulosus*. The cumulative survival rate stratified by sex (A), age (B), and dog type (C).

Figure 1

Click here to access/download;Figure;Figure_1.tif

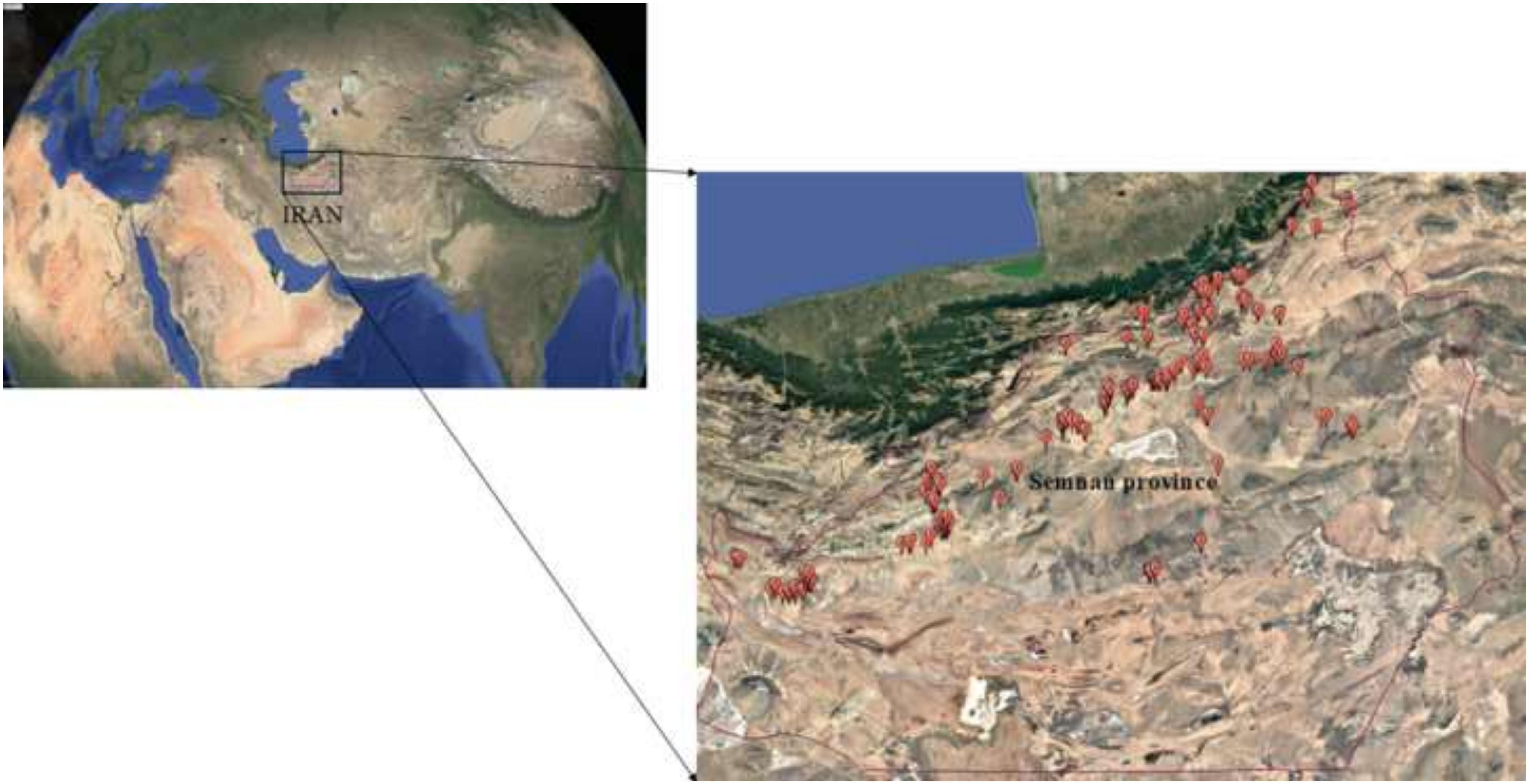

Figure 2

Click here to access/download;Figure;Figure_2.jpg

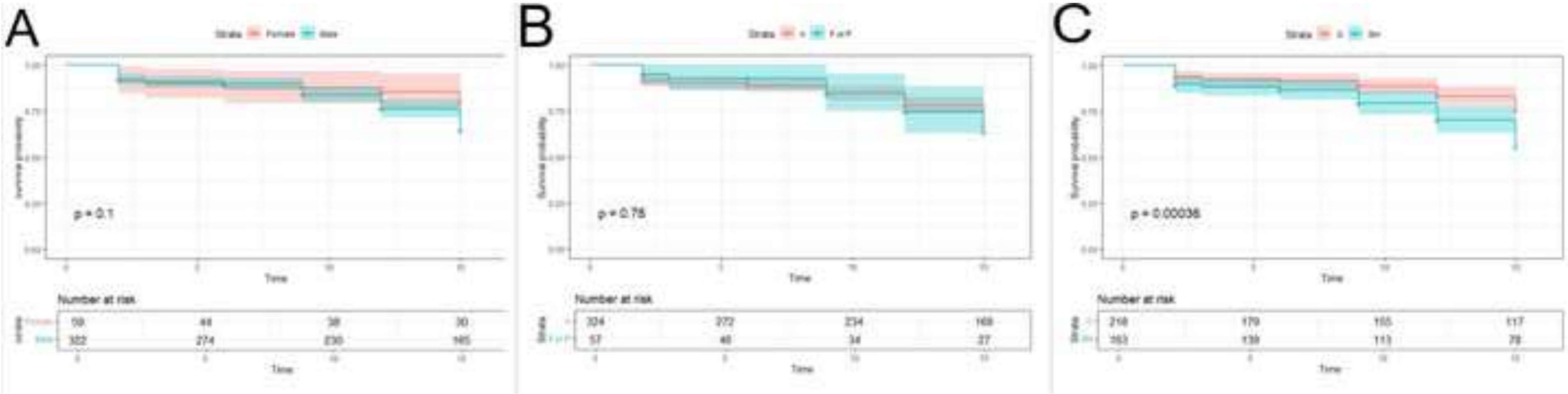

Click here to access/download
Supporting Information
Re_infection_Data_Echino_14040509.xlsx

Dear Editor-in-Chief, PLoS NTD

We appreciate your feedback regarding the manuscript PNTD-D-25-01258 entitled "Reinfection dynamics of owned dogs with Echinococcus granulosus in endemic rural areas of central Iran: Implications for cystic echinococcosis control”. The manuscript is revised based on the editorial comments, with the revised sections highlighted in the main text.

Thank you and best regards

M. Fasihi Harandi & M. Fallah, the Corresponding authors

Additional Editor Comments (if provided):

Comment. While the subject area is within the remit of the PLoS NTD journal, the manuscript is not currently in a suitable format for the journal. In particular the results section is very short and should be expanded. I recommend the authors make these changes, expanding the results section in line with a research article rather than a short communication, and resubmit the manuscript for review.

Response. Thanks for the comment. The findings of the study are expanded both in the Abstract and the Results sections. More data are provided in these sections. The Results section is expanded and the findings are detailed in three different subheadings, including Study Population, Baseline Prevalence and genotyping, Reinfection Dynamics and Survival Analysis, and Risk Factor Analysis for Reinfection.

Thanks for your kind attention.
