# Supplementary material for: Reinfection dynamics of owned dogs with Echinococcus granulosus sensu lato in endemic rural areas of central Iran: Implications for cystic echinococcosis control
Source: PLoS Negl Trop Dis. 2026 Jun 29;20(6):e0014451. doi: 10.1371/journal.pntd.0014451 (PMC13327511; doi:10.1371/journal.pntd.0014451)
Supplement: Attachment — Submitted filename: PNTD-D-25-01258_R1.pdf [file pntd.0014451.s008.pdf]

PLOS Neglected Tropical Diseases

Reinfection dynamics of owned dogs with Echinococcus granulosus sensu lato in endemic rural areas of central Iran: Implications for cystic echinococcosis control

--Manuscript Draft--

**Manuscript Number:**	PNTD-D-25-01258R1
**Full Title:**	Reinfection dynamics of owned dogs with Echinococcus granulosus sensu lato in endemic rural areas of central Iran: Implications for cystic echinococcosis control
**Short Title:**	Reinfection dynamics of the owned dogs with Echinococcus granulosus in Iran
**Article Type:**	Research Article
**Keywords:**	Canine echinococcosis; Anthelmintic treatment; Reinfection; transmission dynamics
**Abstract:**	Background<br><br>Cystic echinococcosis (CE), caused by the tapeworm Echinococcus granulosus,sensu lato (E. granulosus s. l.), is highly endemic in many regions of the world including Iran, with dogs as definitive hosts, playing a critical role in the dynamics of CE transmission. Regular deworming of dogs is an essential part of any successful CE control program. However, dogs can become easily reinfected after praziquantel (PZQ) administration, and the rate of reinfection of dogs in an endemic area is considered an indicator of the intensity of CE transmission. This study aimed to evaluate reinfection rate in the owned dogs after praziquantel treatment in Semnan province, in North of Iran.<br><br>Methods<br><br>A cohort of 381 guard and sheepdogs was monitored for 15 months post-treatment with praziquantel. Reinfection was assessed at 3, 6, 9, 12, and 15 months by fecal sampling. Samples were microscopically examined for taeniid eggs after formalin-ether sedimentation, and were confirmed by PCR using E. granulosus s. l.-specific primers. Kaplan-Meier survival analysis was used to calculate the cumulative survival rate over a 15-month period. A Cox proportional hazards regression model was employed to identify risk factors associated with the time to reinfection with E. granulosus s.l.<br><br>Results<br><br>Fecal samples from 151 sheepdogs and 230 guard dogs from 290 farms, were examined. At baseline, microscopic and copro-PCR analyses revealed that 12.6% positive for taeniid eggs, of which 31 samples (8.1%) were positive for E. granulosus s. l. The reinfection rate was estimated as 9.6%, 10.7%, 15.6%, 22.5%, and 33.2% at 3, 6, 9, 12, and 15 months after initial treatment with praziquantel, respectively. Sheepdogs showed significantly higher reinfection risks (29.8%) compared to the guard dogs (16.7%). Dogs whose owners practiced home slaughter were 2.02 times more likely to become reinfected. Only 17.3% (66/381) of the dogs were reported to be regularly dewormed by the owners.<br><br>Conclusion<br><br>This study demonstrates that re-infection of dogs with E. granulosus s.l. is common in CE-endemic regions of Iran. The high rate of re-infection has significant implications for CE control, particularly in determining the most effective frequency for administering praziquantel to prevent sustained transmission.
**Additional Information:**	
**Question**	**Response**
**Government Employee**<br><br>Are you or any of the contributing authors	No - No authors are employees of the U.S. government.

an employee of the United States government?<br><br>Manuscripts authored by one or more US Government employees are not copyrighted, but are licensed under a CC0 Public Domain Dedication, which allows unlimited distribution and reuse of the article for any lawful purpose. This is a legal requirement for US Government employees.<br><br>This will be typeset if the manuscript is accepted for publication.	
**Financial Disclosure**<br><br>Enter a financial disclosure statement that describes the sources of funding for the work included in this submission. Review the submission guidelines for detailed requirements. View published research articles from *PLOS Neglected Tropical Diseases* for specific examples.<br><br>This statement is required for submission and **will appear in the published article** if the submission is accepted. Please make sure it is accurate.<br><br>**Funded studies**<br>Enter a statement with the following details:<br>• Initials of the authors who received each award<br>• Grant numbers awarded to each author<br>• The full name of each funder<br>• URL of each funder website<br>• Did the sponsors or funders play any role in the study design, data collection and analysis, decision to publish, or preparation of the manuscript?<br><br>Did you receive funding for this work?	Yes
Please add funding details.<br>as follow-up to "**Financial Disclosure**<br><br>Enter a financial disclosure statement that	The Vice Chancellor of Research and Technology of Hamadan University of Medical Sciences dedicated a grant for this research (Grant Number: 40799).

describes the sources of funding for the work included in this submission. Review the submission guidelines for detailed requirements. View published research articles from *PLOS Neglected Tropical Diseases* for specific examples.<br><br>This statement is required for submission and **will appear in the published article** if the submission is accepted. Please make sure it is accurate.<br><br>**Funded studies**<br>Enter a statement with the following details:<br>• Initials of the authors who received each award<br>• Grant numbers awarded to each author<br>• The full name of each funder<br>• URL of each funder website<br>• Did the sponsors or funders play any role in the study design, data collection and analysis, decision to publish, or preparation of the manuscript?<br><br>Did you receive funding for this work?"	
Please select the country of your main research funder (please select carefully as in some cases this is used in fee calculation).<br>as follow-up to "**Financial Disclosure**<br><br>Enter a financial disclosure statement that describes the sources of funding for the work included in this submission. Review the submission guidelines for detailed requirements. View published research articles from *PLOS Neglected Tropical Diseases* for specific examples.<br><br>This statement is required for submission and **will appear in the published article** if the submission is accepted. Please make sure it is accurate.	IRAN, ISLAMIC REPUBLIC OF - IR

Powered by Editorial Manager® and ProduXion Manager® from Aries Systems Corporation

**Funded studies**<br>Enter a statement with the following details:<br>• Initials of the authors who received each award<br>• Grant numbers awarded to each author<br>• The full name of each funder<br>• URL of each funder website<br>• Did the sponsors or funders play any role in the study design, data collection and analysis, decision to publish, or preparation of the manuscript?<br><br>Did you receive funding for this work?"	
**Competing Interests**<br><br>On behalf of all authors, disclose any competing interests that could be perceived to bias this work.<br><br>This statement will be typeset if the manuscript is accepted for publication.<br><br>Review the instructions link below and PLOS NTDs' competing interests policy to determine what information must be disclosed at submission.	The authors have declared that no competing interests exist
**Data Availability**<br><br>Provide a **Data Availability Statement** in the box below. This statement should detail where the data used in this submission can be accessed. This statement will be typeset if the manuscript is accepted for publication.<br><br>Before publication, authors are required to make all data underlying their findings fully available, without restriction. Review our PLOS Data Policy page for detailed information on this policy. Instructions for writing your Data Availability statement can be accessed via the Instructions link below.	The data that support the findings of this study are available as Supporting Information file.

Manuscript Click here to access/download;Manuscript;SRM_manuscript_14041007_cle

Reinfection dynamics of owned dogs with *Echinococcus granulosus* sensu lato in endemic rural areas of central Iran: Implications for cystic echinococcosis control

Seyed Reza Mirbadie [1, 2], Saeedeh Shamsaddini[3], Mohammad Taghi Rahimi[2], Mohammad Matini[1], Mohammad Ali Mohaghegh[4], Mohammad Reza Baneshi[5], Majid Fasihi Harandi[3*], Mohammad Fallah[1*]

1. Department of Medical Parasitology and Mycology, School of Medicine, Hamadan University of Medical Sciences, Hamadan, Iran

2. School of Medicine, Shahroud University of Medical Sciences, Shahroud, Iran

3. Research Center for Hydatid Disease in Iran, Institute of Infectious Diseases and Tropical Medicine, Afzalipour School of Medicine, Kerman University of Medical Sciences, Kerman, Iran

4. Department of Laboratory Sciences, School of Paramedical Sciences, Torbat Heydariyeh University of Medical Sciences, Torbat Heydariyeh, Iran.

5. The University of Queensland, Australian Women and Girls' Health Research Centre, School of Public Health, Herston, Queensland, Australia.

* Correspondences: majid.fasihi@gmail.com ; mohfall@yahoo.com

ABSTRACT

Background Cystic echinococcosis (CE), caused by the tapeworm *Echinococcus granulosus*,sensu lato (*E. granulosus* s. l.), is highly endemic in many regions of the world including Iran, with dogs as definitive hosts, playing a critical role in the dynamics of CE transmission. Regular deworming of dogs is an essential part of any successful CE control program. However, dogs can become easily reinfected after praziquantel (PZQ) administration, and the rate of reinfection of dogs in an endemic area is considered an indicator of the intensity of CE transmission. This study aimed to evaluate reinfection rate in the owned dogs after praziquantel treatment in Semnan province, in North of Iran.

Methods: A cohort of 381 guard and sheepdogs was monitored for 15 months post-treatment with praziquantel. Reinfection was assessed at 3, 6, 9, 12, and 15 months by fecal sampling. Samples were microscopically examined for taeniid eggs after formalin-ether sedimentation, and were confirmed by PCR using *E. granulosus* s. l.-specific primers. Kaplan-Meier survival analysis was used to calculate the cumulative survival rate over a 15-month period. A Cox proportional hazards regression model was employed to identify risk factors associated with the time to reinfection with *E. granulosus* s.l.

Results: Fecal samples from 151 sheepdogs and 230 guard dogs from 290 farms, were examined. At baseline, microscopic and copro-PCR analyses revealed that 12.6% positive for taeniid eggs, of which 31 samples (8.1%) were positive for *E. granulosus* s. l. The reinfection rate was estimated as 9.6%, 10.7%, 15.6%, 22.5%, and 33.2% at 3, 6, 9, 12, and 15 months after initial treatment with praziquantel, respectively. Sheepdogs showed significantly higher reinfection risks (29.8%) compared to the guard dogs (16.7%). Dogs whose owners practiced home slaughter were 2.02 times more likely to become reinfected. Only 17.3% (66/381) of the dogs were reported to be regularly dewormed by the owners.

Conclusion: This study demonstrates that re-infection of dogs with *E. granulosus* s.l. is common in CE-endemic regions of Iran. The high rate of re-infection has significant implications for CE control, particularly in determining the most effective frequency for administering praziquantel to prevent sustained transmission.

Keywords: Canine echinococcosis; Anthelmintic treatment; Reinfection; Transmission dynamics

AUTHOR SUMMARY

Cystic echinococcosis is a long-standing disease that continues to affect people and livestock in many rural regions of the world. ~~Because~~ dogs are essential for the parasite to survive and spread, controlling infection in dogs is ~~central~~ to preventing disease in humans. However, dogs often become infected again soon after treatment, which makes disease control extremely difficult. In this study, we followed more than three hundred farm dogs in northern Iran for fifteen months after treatment to understand how quickly and how frequently this reinfection occurs.

We found that nearly one third of treated dogs became infected again within this period. Sheepdogs and the dogs whose owners practiced home slaughter were significantly at higher reinfection risks. Only a small fraction of dog owners treated their animals regularly, which further increased the chance of reinfection. By measuring the time until dogs became infected again, we were able to identify the practices that maintain disease transmission in these communities. Our findings show that treating dogs alone is not sufficient. Sustainable control of cystic echinococcosis requires changes in animal husbandry, safer disposal of animal remains, and improved community awareness to reduce long-term risk for both animals and people.

INTRODUCTION

Cystic echinococcosis, caused by the small tapeworm of canids *Echinococcus granulosus* sensu lato (*E. granulosus* s. l.), is a major parasitic zoonosis of humans in the world affecting livestock and dogs as the intermediate and definitive hosts, respectively. Currently, more than one million people worldwide are infected with cystic echinococcosis (CE), and this condition is considered a serious public health challenge (1, 2). The impact of this disease is primarily on the marginalized and rural communities with low socioeconomic status and inadequate management and control over dogs.

Global reports of the incidence of cystic echinococcosis have estimated 207,368 new cases with 122,457 DALYs (3). The surgical incidence of CE varies between 1 and 200 cases per 100,000 people worldwide. CE is endemic with high incidence in most Middle East and North African countries including Iran, where the incidence is estimated 0.61-1.27 per 100,000 (4). Considering the close relationship between dogs and humans, dogs play a key role in CE transmission. The overall estimated prevalence of infection in dogs in Iran is 23.6% (5, 6). CE imposes substantial economic and social burdens on affected communities, encompassing the costs of medical and surgical management and the resulting disability in humans, as well as significant losses to the livestock sector (7, 8). In Iran alone, the overall annual monetary burden of the disease has been estimated at approximately US$232 million (9, 10).

The role of dogs as the main definitive host and reservoir of the parasite is crucial in the spread of parasite eggs in the environment through canine feces. Guard dogs and sheepdogs are essential in the activity of the people living in the rural and semi-rural regions in Iran, where agriculture and animal husbandry is their major occupation (11, 12). One of the key factors in CE transmission in most parts of the world is the close relationship of dogs, livestock, and humans. Studies indicate that the global dog population has surpassed 700 million, of which

75% are owned and unowned free-roaming dogs. The ratio of dogs to humans ranges from 0.2 to >20 dogs per 100 people in different countries (13-15).

~~Necessary~~ measures for CE control include livestock vaccination, regular dog deworming, dog registration, dog population management, improving abattoir infrastructure, controlling home slaughter and dog access to livestock offal (3, 12). Echinococcosis in dogs is very difficult to control and to date, only a limited number of control programs in a few countries have been successful (16, 17). Various studies have shown that regular deworming of dogs plays a substantial role in reducing the incidence of canine echinococcosis (12, 18, 19). Reinfection of dogs remains one of the major obstacles to controlling cystic echinococcosis in endemic communities. Although dog deworming is the central and most effective intervention for interrupting transmission, its impact is often undermined because dogs frequently become reinfected when they continue to access contaminated offal or roam freely.

Dog deworming campaigns have been hampered by frequent reinfection of dogs following access to the infected livestock offal. Dogs can become easily reinfected after praziquantel (PZQ) administration, and the rate of reinfection of dogs in an endemic area is considered an indicator of the intensity of CE transmission (20, 21). This constant reinfection cycle is a key reason why many CE control programs fail to achieve long-lasting results. However, our understanding of how often dogs are reinfected, how quickly this occurs, and which community practices contribute to it is still limited. Without reliable knowledge of reinfection dynamics, it is not possible to design effective interventions (20). These gaps highlight the urgent need for field studies in the endemic areas on dog reinfection to support the development of successful and sustainable CE control strategies.

Studies conducted in two North African countries, Morocco and Tunisia, demonstrated that administering treatment to dogs at two-month intervals was more effective in controlling

reinfection compared to less frequent regimens (11, 22). The optimal frequency of praziquantel administration is largely determined by how rapidly dogs become reinfected in a given endemic region, alongside additional ecological and management factors (21). Dog deworming campaigns have been challenged by major logistic and administrative problems related to the frequent site visits and anthelmintic treatment. Knowledge about the nature and extent of reinfection in dogs is essential for any CE control program. Reinfection studies are required to provide baseline information for control and prevention of CE in endemic areas.

Very few reinfection studies have been conducted in Iran. In a recent study in southeast of the country, reinfection of owned dogs reached 17% one year after initial PZQ treatment with a monthly reinfection incidence of 1.5% (20). Obviously, further studies on dog reinfection are required to improve our understanding of the dynamics of CE transmission in different endemic regions as an essential requisite for accomplishing a successful CE control program.

This study was designed to quantify how often reinfection occurs in owned dogs and identify the contributing factors in endemic communities. Specifically, we aimed to estimate the cumulative incidence of *E. granulosus* sensu lato reinfection after praziquantel treatment and examine demographic, behavioral, and environmental variables that may increase reinfection risk. This approach was intended to generate evidence needed to inform more effective deworming strategies.

MATERIALS AND METHODS

Ethics statement

The project was approved by the ethical review committee of Hamadan University of Medical Sciences (IR.UMSHA.REC.1401.877). The study objectives were fully explained to the dog owners, emphasizing the importance of the study. Prior to sampling, verbal consent was obtained from the owners of the dogs. With the owners' consent, all dogs present at each

respective farm or herd were sampled, which were primarily working animals such as sheepdogs and guard dogs.

Study area

This cross-sectional study was conducted in Semnan Province, Iran. Semnan is located between the edge of the desert plain (central desert of Iran) and the Alborz Mountain range with a high-altitude variation between 1000 and 3000 m above sea level. The province is located between latitude 34°13′-37°20′N and longitude 51°51′-57°3′E in north-central Iran with a surface area of 97,491 Km^2 and a population of 702,360 (Fig. 1). Semnan has a diverse climate with a temperate climate in the northern areas and arid and semi-arid regions in the south. The average annual rainfall is 139.5 mm. (https://www.worldweatheronline.com/semnan-weather-averages/semnan/ir.aspx) with 13.6 million acres of pasture and 2.5 million livestock. (https://amar.org.ir/) (https://semnan-aj.ir/)

Study design and Sample collection

In total, 290 farms from 101 villages were randomly selected from five counties in Semnan province, including Semnan, Shahroud, Damghan, Mayamey, and Garmsar. A total of 381 dogs were recruited in the study (Fig. 1). The living place of the dogs and their roaming status were recorded and sampling was done as follows. For those dogs that were tied up constantly or only during the day, the fresh feces samples were collected from inside and/or around their living place. Most samples from herding dogs were collected from their resting areas. Shepherds and dog owners were asked to collect fecal samples from each dog immediately after defecation. Some dogs were also restrained or tied up until defecation and sample collection (Fig. 2).

Approximately 100 grams of stool sample from each dog were placed in plastic containers in cold boxes and transferred promptly to the laboratory for parasitological examination. %50 of

the sample was preserved in 10% formalin for sedimentation test and microscopic examination, and another for DNA extraction and molecular work. For safety reasons, all samples were kept at -80°C for at least ten days.

Parasitological and Molecular analyses

All samples were microscopically examined for taeniid eggs following formalin-ether concentration technique. Subsequently, taeniid eggs were recovered from fecal material using sugar flotation. Sugar flotation method was applied to the positive samples as described elsewhere with some modifications (23). Briefly, high density sugar solution with a specific gravity of 1.27 was added to each sample and the resulting suspension was centrifuged at 500 g for 10 min. In the next step, taeniid eggs were collected from the supernatant and stored in -20 °C until used for molecular analysis.

All dogs found infected with taeniid eggs were treated with PZQ, 5 mg/kg of body weight. Seven days after anthelmintic treatment, re-sampling was done to check the outcome of the treatment. Re-treatment was performed until a negative result was obtained. In total, only 4 dogs were positive 7 days after the initial treatment, which were re-treated and tested negative for infection after re-sampling. Re-samplings were performed to investigate the re-infection rate of the dogs at 3, 6, 9, 12, and 15 months after the initial administration of PZQ. The dogs did not receive any further treatment during the entire study period.

For molecular analysis, PCR-sequencing was performed using three primer pairs amplifying partial cytochrome c oxidase (COX1) gene (444 bp), a 133-bp fragment of a tandem repeat sequence, and a partial SSU-rDNA region (149 bp). The cox1 primer pair was taeniid-specific and the other two primer sets were used for the specific detection of *E. granulosus* s. l. (20, 24, 25). Table 1 shows the primer sequences used in the present study. Previously sequenced DNA samples were used as positive controls (7).

For genotype identification, the PCR products obtained from *E. granulosus* s. l.-positive samples were randomly selected, purified and sent for Sanger sequencing (Pishgam co. Tehran) by targeting SSU-rDNA using the reverse primer (Table 1).

The sequence data were edited and analyzed by Sequencher 4.7 and MEGA 5.05. The obtained sequences were examined using the NCBI BLAST database for the final identification and confirmation of *E. granulosus* s. l. genotypes.

Dog data collection

A data collection sheet was filled for each dog before fecal sampling. Data related to each dog were recorded including age, sex, location, anthelmintic treatment, type of dog food, and roaming habits. Specific dog characters were recorded for identification of dogs during follow-up. In addition, dog owners were asked about home slaughter and disposal of sheep carcasses and infected organs.

Statistical analysis

The Kaplan-Meier method was used to calculate and visualize the cumulative survival rate stratified by age, sex, and type of dog. Cumulative incidence was defined as the complement of survival rates (i.e., one minus survival rates). Time was considered as the variable of infection time for dogs with reinfection, and the time of the last microscopic examination was defined as the censored time for uninfected dogs.

To identify risk factors associated with the time to reinfection with *E. granulosus* s. l., a Cox hazards regression model was employed. Eight variables including gender, age, dog type, roaming behavior, feeding habits, anthelmintic treatment, location, and home slaughter were evaluated using Cox analysis. Adjusted Hazard Ratios (HRs) and their 95% confidence intervals (CIs) were estimated to quantify the magnitude of association between each

covariate and the risk of reinfection. All statistical analyses were conducted using R software Supporting Information file 1.

RESULTS

Study Population, Baseline Prevalence and genotyping

Fecal samples from a total of 381 owned dogs, including 151 sheepdogs and 230 guard dogs from 290 farms, were collected between February 2023 and May 2024 in different villages in five counties of Semnan province (Fig. 1). Over an 18-month period, the dogs were evaluated for re-infection with *E. granulosus* s. l. Microscopic study and copro-PCR analyses showed a total of 48 samples (12.6%) as positive for taeniid eggs before the start of the deworming program, of which 31 samples (8.1%) were positive for *E. granulosus s. l.* Taeniid eggs were detected in 20 guard dogs and 28 sheepdogs, among which *E. granulosus* s. l. was identified in 14 (6.1%) and 17 (11.2%) individuals, respectively (Table 2, Fig. 2). Nine *E. granulosus* s. l.-positive samples were PCR-sequenced on a partial SSU-rDNA (~149 bp) and compared with the sequences in NCBI GenBank. Eight isolates were identified as *E. granulosus* sensu stricto (s.s.) (G1-G3) and one as *E. canadensis* G6-G7.

Reinfection Dynamics and Survival Analysis

Following initial treatment with praziquantel, the cohort of dogs was monitored for reinfection over a 15-month period. Table 3 presents the survival rates and cumulative incidence of infection at 3, 6, 9, 12, and 15 months post-treatment with praziquantel. The cumulative incidence of reinfection with *E. granulosus* s. l. progressively increased throughout the follow-up. At 3 months post-treatment, the cumulative incidence was 9.6% (95% CI: 6.5-12.5%). This rate rose to 10.7% (95% CI: 7.5-13.7%) at 6 months, 15.6% (95% CI: 11.9-19.1%) at 9 months, and 22.5% (95% CI: 18.0-26.8%) at the 12-month mark. By the

end of the 15-month study period, the cumulative incidence of reinfection reached 33.2% (95% CI: 27.7-38.3%) (Table 3).

Kaplan-Meier survival analysis was used to visualize the time to reinfection (Figure 3). The analysis, when stratified by dog type, showed a significantly lower survival probability (i.e., a higher rate of reinfection) for sheepdogs compared to guard dogs over the 15-month period (Figure 3C). The final reinfection risk was 29.8% for sheepdogs versus 16.7% for guard dogs. No significant differences in survival rates were observed when dogs were stratified by sex or age (Figure 3A, 3B).

Risk Factor Analysis for Reinfection

A Cox proportional hazards regression model was employed to identify risk factors associated with the time to reinfection. Table 4 shows hazard ratios obtained from analyzing the potential risk of echinococcosis in the owned dogs in rural areas of Semnan province. The analysis revealed several significant predictors. Dog type was a primary risk factor, with sheepdogs having a 1.8 times higher risk of reinfection compared to guard dogs (Adjusted Hazard Ratio [HR]: 1.8, 95% CI: 1.22-2.68; $p<0.05$). Practices related to livestock slaughter and feeding were also strongly associated with reinfection. Dogs whose owners practiced home slaughter were 2.02 times more likely to become reinfected (HR: 2.02, 95% CI: 1.35-3.03; $p<0.05$). Similarly, feeding dogs with raw livestock offal was identified as a significant risk factor ($p=0.001$).

Furthermore, the history of anthelmintic treatment was a significant factor. Dogs that were not regularly dewormed by their owners had a 1.76 times greater risk of reinfection than those receiving regular treatment (HR: 1.76, 95% CI: 1.04-2.97; $p<0.05$). Data collected from owners indicated that only 17.3% (66/381) of the dogs in the study were reported to be regularly dewormed prior to the study's intervention. Other variables, including the dog's

gender, age, roaming behavior, and location, were not found to be significantly associated with the risk of reinfection in the final model (Table 4).

DISCUSSION

Echinococcosis is a major parasitic zoonosis and has been classified by the World Health Organization as a Neglected Tropical Disease (NTD), with control efforts prioritized under the 2030 global roadmap (26). The disease is endemic in Iran, and domestic dogs are the main source of human infection. The prevalence of *E. granulosus* s. l. in dogs has been reported to range from 6.8% to 55.7% in different regions of Iran. According to recent studies, Semnan province in central Iran is one of the important foci of the CE in the country, with a prevalence of 7.4% in dogs, 13.3% in livestock, and 8.6% seroprevalence in humans (4, 27).

Dogs play a critical and sustaining role in the life cycle and transmission of CE. As the definitive hosts of *E. granulosus*, dogs harbor the adult tapeworm in their intestines and shed large numbers of infective eggs into the environment. When livestock ingest these eggs, the larval stage develops in their visceral organs, completing the intermediate-host part of the cycle. Dogs become readily re-infected when they consume raw infected offal during home slaughter or scavenging, allowing new adult worms to mature and resume egg shedding. This repeated cycle and continual re-infection creates a persistent environmental source of contamination, highlighting the need for reducing dog re-infection through regular deworming, responsible slaughter practices, and proper offal disposal to break the transmission cycle.

Regular dog deworming by the oral administration of PZQ at scheduled intervals is the first option in implementing CE control programs in endemic regions. However, re-infection of dogs after treatment is a major challenge in CE control programs. It has been shown that lack

of treatment or inadequate treatment intervals increase the odds of reinfection by 23.3-52.5 (18).

To assess the re-infection rate of echinococcosis in dogs, a 15-month follow-up study was conducted at 3, 6, 9, 12, and 15 months after treatment with praziquantel. The rate of reinfection in dogs after treatment increased over time, such that 9.6% (6.5-12.5%) of dogs were reinfected with *E. granulosus* s. l., three months after the initial treatment. The infection rate reached 22.5% (18.0-26.8%) one year after initial deworming (Table 3). High reinfection of farm dogs occurs following home slaughter and/or feeding offal to the owned dogs. Consequently, regular and frequent dog deworming is required to overcome the infection pressure in dogs. This presents major logistic and financial challenges for implementing effective CE control programs in endemic areas, as the frequency of PZQ dosing is related to dog reinfection pressure.

The rate of reinfection in owned dogs, particularly guard and sheepdogs, has been investigated in few studies worldwide, including Iran. Table 5 shows the reinfection studies conducted in different endemic countries. Findings of these studies indicate that in various endemic countries the reinfection rate ranges from 1.56% to 27.9% one year after initial treatment. A study in Libya estimated reinfection in 334 owned dogs. The rate of reinfection 15 months after treatment with PZQ reported to be 22.2% (28). In Peru it was shown that failure of regular deworming of dogs increases the rate of reinfection (29). In Uruguay, reinfection in dogs occurred after 2 to 4 weeks (17). In the two studies conducted in Morocco and Iran, the monthly reinfection rates in domestic dogs were reported to be 4% and 1.5%, respectively (12, 20).

One single study was conducted in Iran to investigate reinfection in farm dogs in Kerman, and the reinfection rate one year after the study was 17%. In the present study 22.5% of the

dogs were reinfected after one year and 33.2% after 15 months. Interestingly, higher reinfection was observed in the sheep dogs (29.8%) comparing to the guard dogs (16.7%). This is consistent with the findings of other studies conducted in Libya and Kazakhstan. In Libya, herd dogs are 9 times more likely to be copro-Ag positive than domestic dogs (28, 30). Also, findings of another study in Morocco indicate that the reinfection rate in stray dogs was higher than in owned dogs (22). The higher chance of reinfection in the sheepdogs can be due to a higher chance of feeding with offal, free-roaming behavior, and home slaughter.

Variations in reinfection rates across different endemic regions may be attributed to differences in animal husbandry and slaughter practices, cyst fecundity, frequency of dog deworming, diversity of intermediate hosts, genotype-related factors, as well as socioeconomic, cultural, and lifestyle differences. Additionally, the use of different diagnostic methods may further contribute to these discrepancies. (31, 32).

Repeated re-infection of farm dogs with *E. granulosus* s. l. poses significant challenges for CE control. As definitive hosts, farm dogs in the CE-endemic regions, are particularly vulnerable to re-infection due to their frequent exposure to infected offal from home-slaughtered livestock, scavenging on carcasses, and inadequate access to regular anthelmintic treatment. The frequent re-infections undermine control efforts, as even treated dogs become reinfected when traditional or existing animal husbandry, slaughtering, and dog-keeping behaviors remain unchanged (17, 20, 21, 32). Moreover, the persistent contamination of pastures, water sources, and farm environments with *Echinococcus* eggs sustains parasite circulation and increase the risk of human infection through accidental ingestion of eggs.

The implementation of regular canine deworming programs as a strategy for CE control faces significant operational and biological challenges that undermine effective CE control measures. The short prepatent period in canine echinococcosis (5-6 weeks) imply monthly

PZQ administration to prevent egg shedding, however, this frequency is logistically demanding and economically unsustainable in endemic communities. Many endemic areas lack adequate veterinary infrastructure, resulting in inconsistent drug distribution and treatment coverage gaps. The European Scientific Council on Companion Animal Parasites (ESCCAP), along with several other studies, has proposed varying deworming guidelines for dogs, recommending treatment intervals ranging from monthly, bimonthly, and every 3 to 4 months, to as infrequently as once per year. (18, 22, 31) . In-depth experimental field and modeling studies in different endemicity scenarios are required to establish the most effective deworming regimen in each endemic society.

Control programs often fail in rural settings due to limited veterinary infrastructure, poor compliance with deworming protocols, and traditional livestock husbandry, while limited community awareness of zoonotic risks reduces participation in control initiatives. A successful intervention program must integrate regular PZQ administration, livestock vaccination, regulation of offal disposal, and community education, supported by long-term political commitment and international cooperation. Without implementing these measures, long-term CE control efforts face serious challenges. Future studies should explore practical, cost-effective surveillance tools and dog vaccination strategies to break the cycle of dog re-infection in endemic countries (3, 12). In addition, while the study focused on owned dogs, the presence of unowned dog populations in the same areas, were not quantified. Free-roaming dog populations frequently evade treatment efforts, while wildlife reservoirs in some regions contribute to environmental contamination. Further studies are required to investigate the nature and extent of reinfection in unowned free-roaming dogs in the endemic regions.

Due to the lack of longitudinal survival studies, Cox analyses and hazard ratios (HRs) are rarely reported in the studies to understand the factors associated with echinococcosis in dogs. However, risk ratios (RRs) and/or odds ratios (ORs) are frequently used in

epidemiological studies to quantify associations between risk factors and canine echinococcosis. In the present study the dog type (guard vs. sheepdog), feeding dogs with livestock viscera, anthelmintic treatment of dogs, and home slaughter practice were significantly associated with the risk of *Echinococcus* infection. Several studies have identified dog-keeping practices, and access to raw livestock offal as the predominant risk factors of canine CE (18, 33). Dogs fed uncooked viscera in Peru exhibit 2.9 times (95% CI, 1.0–8. Also, in Uganda access of dogs to livestock slaughter facilities significantly increased the risk of canine echinococcosis (OR = 11) (34). Allowing dogs to roam freely (OR=3.17), feeding dogs with viscera (OR=3.04), and slaughter at home (OR=3.53) demonstrate greater odds of *E. granulosus* s. l. infection (35). This practice significantly sustains parasite lifecycles in the rural environments. Roaming behavior further elevates risk of canine echinococcosis in several studies.

In Libya non-restraint of dogs, home slaughtering of livestock, limited awareness and failure of owners to administer regular deworming treatments, were found as significant risk factors of infection (28). The results of our study also showed that dogs that were constantly free-roamed accounted for more than half of the positive cases. However, in the present study this was not significantly associated with infection in dogs. It is important to note that dogs kept under constant restraint are very likely to be fed infected offal by their owners.

Our results showed that dogs whose owners practiced home slaughter were 2.02 times more likely to be re-infected with *E. granulosus* s. l. In pastoral and rural communities, home slaughter bypasses veterinary meat inspection, allowing infected organs to be discarded or fed to dogs, with many studies reporting higher odds of canine infection in such settings (21, 34, 35). This perpetuates egg contamination of rural environments, increasing risk of ingesting *Echinococcus* eggs by humans. Consequently, home slaughter undermines abattoir regulations and CE control programs.

The results of our questionnaire survey showed that only 39.3% of dog owners had sufficient information about the life cycle and routes of infection in dogs. In a study in rural Peru, more than 23% of dog owners fed offal to their dogs. A study in Libya found that home slaughter increased the risk of copro-antigen positivity of dogs by 8 times. Various studies consider the lack of awareness in dog owners and low knowledge of CE transmission as the main factors of infection in shepherd and domestic dogs (28, 29). Also, socio-cultural traditions and economic factors are important in resource-limited regions. Interventions targeting this practice through community education, subsidized slaughter facilities, and incentivized suitable offal disposal, are critical to disrupt CE transmission. Without addressing home slaughter, sustained reductions in canine and human echinococcosis remain unachievable (36).

Unregulated disposal of waste from home slaughtering practices significantly increases environmental contamination with Echinococcus eggs. Dogs in rural areas exhibit a 3.5- to 6.0-fold higher prevalence of cystic echinococcosis compared to those in urban settings, reflecting closer contact with livestock and limited access to veterinary services and control measures (30). Inadequate deworming heightens these risks, as untreated dogs exhibit higher infection odds than regularly dewormed counterparts (33). Despite PZQ efficacy, logistical barriers including cost, accessibility, and community compliance, undermine sustained deworming in resource-limited regions. Diagnostic variability e.g., coproantigen ELISA, copro-PCR and microscopy, may also affect accuracy.

According to previous studies, the genotype of the parasite is another important factor influencing the effectiveness of deworming interventions in dogs (37). Our findings indicated that *E. granulosus* s.s. (G1/G3) and *E. canadensis* genotypes were present in dogs, in line with previous studies in other endemic regions of the country (38). In Semnan Province, genotypes G1, G3, G6, and G7 of *E. granulosus* s. l. have been identified in both stray and

owned dogs, as well as in livestock isolates (7, 27). The pre-patent period of *E. granulosus* s. l. in dogs shows variation across different genotypes, particularly between *E. granulosus* sensu stricto (G1–G3) and *E. canadensis* (G6–G7). In experimental infections, G1 genotype exhibits a relatively short pre-patent period with adult worms becoming mature and shedding eggs within 6 weeks post-infection. In contrast, infections with the G6/G7 genotypes, show a longer pre-patent period, ranging from 45 to 60 days. This presents important implications for epidemiological surveillance, transmission dynamics and hydatid control strategies, with a significant effect on the timing of deworming interventions in regions where G1/G3 or G6/G7 genotypes are dominant (17).

CONCLUSION

This longitudinal study demonstrates the persistent challenge of *E. granulosus* s. l. reinfection in farm dogs despite PZQ treatment. The study underscores the challenges in controlling CE in endemic regions, where persistent *E. granulosus* s. l. reinfection in dogs undermines control efforts. The high reinfection rates observed highlight how husbandry practices, such as home slaughter and offal feeding, sustain parasite transmission cycles. The findings underscore how unregulated home slaughter perpetuates transmission by enabling access to contaminated offal. Critically, the findings reveal that dog deworming are insufficient when environmental and behavioral risk factors remain unaddressed, emphasizing the need for holistic interventions requiring fundamental shifts in local slaughter practices, dog-keeping behaviors, and zoonotic awareness. In Iran little information is available on echinococcosis re-infection in dogs and further studies are required on reinfection in dogs to determine the appropriate treatment intervals, and cost estimates.

The study also calls for adaptive control strategies tailored to regional transmission dynamics, including genotype-specific risks and geographic hotspots. Future research should aim to

perform comprehensive molecular characterization of all cases across different geographical endemic regions, particularly in areas dominated by various *E. granulosus* s.l. genotypes. Such comparative studies are essential to determine whether variations in local genotype distribution influence the likelihood or rate of re-infection. Large-scale multicenter investigations integrating molecular and epidemiological data would substantially improve our understanding of re-infection dynamics and help refine regional control strategies.

Breaking the reinfection cycle needs the integration of veterinary, public health, and community-based approaches, with policy support to ensure long-term feasibility. These insights are critical for advancing One Health strategies against CE in Iran and other endemic settings worldwide.

Abbreviations:

FRDs: Free-roaming dogs

CE: Cystic echinococcosis

nad1: NADH dehydrogenase subunit

AE: Alveolar echinococcosis

Acknowledgements

The authors would like to thank the Vice-Chancellor of Research and Technology of Hamadan University of Medical Sciences for approving and providing financial support for this study. The authors also thank Shahroud University of Medical Sciences for providing its facilities to the project.

Author contributions

Conceptualization and study design: MFH, MF, SRM, SS

Data collection/curation: SRM

Performed the experiments and data analysis: SRM, MFH, SS, MF

Investigation: SRM, MF, MFH, SS

Project supervision: MF, MFH, MM

Writing original draft preparation: SRM, MF, MFH, SS, MAM, MTR

Review and editing the final draft of the manuscript: SRM, SS, MTR, MM, MAM, MFH, MF. The submitted version is approved by all authors.

Funding

The Vice Chancellor of Research and Technology of Hamadan University of Medical Sciences dedicated a grant for this research (Grant Number: 40799). The funders had no role in study design, data collection and analysis, decision to publish, or preparation of the manuscript.

Availability of data and materials

The data that support the findings of this study are available in Supporting Information file 1.

Declarations

Ethics approval and consent to participate

This study was approved by the Ethics Review Committee of Hamadan University of Medical Sciences (Ethical Code: IR.UMSHA.REC.1401.877)

Consent for publication

Not applicable.

Competing interest

The authors declare no competing interests.

Author details

1 Department of Medical Parasitology and Mycology, School of Medicine, Hamadan University of Medical Sciences, Hamadan, Iran

2 School of Medicine, Shahroud University of Medical Sciences, Shahroud, Iran

3 Research Center for Hydatid Disease in Iran, Institute of Infectious Diseases and Tropical Medicine, Afzalipour School of Medicine, Kerman University of Medical Sciences, Kerman, Iran

4 Department of Laboratory Sciences, School of Paramedical Sciences, Torbat Heydariyeh University of Medical Sciences, Torbat Heydariyeh, Iran.

5. The University of Queensland, Australian Women and Girls' Health Research Centre, School of Public Health, Herston, Queensland, Australia.

Authors' emails:

SRM: sedreza.mirbadie@gmail.com

SS: s.shamsadini91@gmail.com

MTR: rahimimt@gmail.com

MM: matini.mohammad@yahoo.com

MAM: mohaghegh1982@yahoo.com

MRB: m.baneshi@uq.edu.au

MFH: majid.fasihi@gmail.com

MF: mohfall@yahoo.com

References:

1. Aghajanzadeh M, Safarpoor F, Amani H, Alavi A. One-stage procedure for lung and liver hydatid cysts. Asian Cardiovasc Thorac Ann. 2008;16(5):392-5.
2. Mohaghegh MA, Yousofi-Darani H, Jafarian AH, Mirbadie SR, Fasihi-Harandi M, Ghavimi R, et al. Isolated Human and Livestock *Echinococcus granulosus* Genotypes Using Real-Time PCR of cox1 Gene in Northeast Iran. Acta Parasitol. 2019;64(3):679-85.
3. Yang Z, Liu K, Wen B, Fu T, Qin X, Li R, et al. Changes in the global epidemiological characteristics of cystic echinococcosis over the past 30 years and projections for the next decade: Findings from the Global Burden of Disease Study 2019. J Glob Health. 2024;14:04056.
4. Borhani M, Fathi S, Darabi E, Jalousian F, Simsek S, Ahmed H, et al. Echinococcoses in Iran, Turkey, and Pakistan: Old Diseases in the New Millennium. Clin Microbiol Rev. 2021;34(3):e0029020.
5. Khalkhali HR, Foroutan M, Khademvatan S, Majidiani H, Aryamand S, Khezri P, Aminpour A. Prevalence of cystic echinococcosis in Iran: a systematic review and meta-analysis. J Helminthol. 2018;92(3):260-8.
6. Mahmoudi S, Mamishi S, Banar M, Pourakbari B, Keshavarz H. Epidemiology of echinococcosis in Iran: a systematic review and meta-analysis. BMC Infect Dis. 2019;19(1):929.
7. Mirbadie SR, Najafi Nasab A, Mohaghegh MA, Norouzi P, Mirzaii M, Spotin A. Molecular phylodiagnosis of *Echinococcus granulosus* sensu lato and *Taenia hydatigena* determined by mitochondrial Cox1 and SSU-rDNA markers in Iranian dogs: Indicating the first record of pig strain (G7) in definitive host in the Middle East. Comp Immunol Microbiol Infect Dis. 2019;65:88-95.
8. Mirbadie S, Zivdari M, Kalani H, Vafaei M, Izadi S, Jabalameli Z, et al. Molecular identification of *Echinococcus granulosus* sensu lato by mitochondrial COX1 and SSU-rDNA markers in dogs in the west of Iran. Gene Reports. 2020;19:100616.
9. Barati L, Shahkar L, Raeiji A, Veghari G. Cystic hydatid disease in children in Gorgan from 2014 to 2021. Iran J Pediatr. 2023;33(5):e136687.
10. Fasihi Harandi M, Budke CM, Rostami S. The monetary burden of cystic echinococcosis in Iran. PLoS Negl Trop Dis. 2012;6(11):e1915.
11. Lahmar S, Sarciron ME, Rouiss M, Mensi M. *Echinococcus granulosus* and other intestinal helminths in semi-stray dogs in Tunisia: infection and re-infection rates. Tunis Med. 2008;86(7):657-64.
12. Amarir F, Rhalem A, Sadak A, Raes M, Oukessou M, Saadi A, et al. Control of cystic echinococcosis in the Middle Atlas, Morocco: Field evaluation of the EG95 vaccine in sheep and cesticide treatment in dogs. PLoS Negl Trop Dis. 2021;15(3):e0009253.
13. Shamsaddini S, Ahmadi Gohari M, Kamyabi H, Nasibi S, Derakhshani A, Mohammadi MA, et al. Dynamic modeling of female neutering interventions for free-roaming dog population management in an urban setting of southeastern Iran. Sci Rep. 2022;12(1):4781.
14. Belsare A, Vanak AT. Modelling the challenges of managing free-ranging dog populations. Sci Rep. 2020;10(1):18874.
15. Smith LM, Hartmann S, Munteanu AM, Dalla Villa P, Quinnell RJ, Collins LM. The Effectiveness of Dog Population Management: A Systematic Review. Animals (Basel). 2019;9(12).
16. Inoue T, Nonaka N, Kanai Y, Iwaki T, Kamiya M, Oku Y. The use of tetracycline in anthelmintic baits to assess baiting rate and drug efficacy against *Echinococcus multilocularis* in foxes. Vet Parasitol. 2007;150(1-2):88-96.

17. Cabrera PA, Lloyd S, Haran G, Pineyro L, Parietti S, Gemmell MA, et al. Control of *Echinococcus granulosus* in Uruguay: evaluation of different treatment intervals for dogs. Vet Parasitol. 2002;103(4):333-40.
18. Weng X, Mu Z, Wei X, Wang X, Zuo Q, Ma S, et al. The effects of dog management on *Echinococcus* spp. prevalence in villages on the eastern Tibetan Plateau, China. Parasit Vectors. 2020;13(1):207.
19. Vienažindienė Ž, Joekel DE, Schaper R, Deplazes P, Šarkūnas M. Longitudinal study for anthelmintic efficacy against intestinal helminths in naturally exposed Lithuanian village dogs: critical analysis of feasibility and limitations. Parasitol Res. 2018;117(5):1581-90.
20. Borhani M, Mohammadi MA, Entezami M, Baneshi MR, Nasibi S, Prada JM, Fasihi Harandi M. Reinfection of farm dogs following praziquantel treatment in an endemic region of cystic echinococcosis in southeastern Iran. PLoS Negl Trop Dis. 2024;18(3):e0011939.
21. Wang YH, Rogan MT, Vuitton DA, Wen H, Bartholomot B, Macpherson CN, et al. Cystic echinococcosis in semi-nomadic pastoral communities in north-west China. Trans R Soc Trop Med Hyg. 2001;95(2):153-8.
22. Amarir FE, Saadi A, Marcotty T, Rhalem A, Oukessou M, Sahibi H, et al. Cystic echinococcosis in three locations in the middle atlas, Morocco: Estimation of the infection rate in the dog reservoir. Vector Borne Zoonotic Dis. 2020;20(6):436-43.
23. Hidalgo A, Melo A, Romero F, Hidalgo V, Villanueva J, Fonseca-Salamanca F. DNA extraction in *Echinococcus granulosus* and *Taenia* spp. eggs in dogs stool samples applying thermal shock. Exp Parasitol. 2018;186:10-6.
24. Abbasi I, Branzburg A, Campos-Ponce M, Abdel Hafez SK, Raoul F, Craig PS, Hamburger J. Copro-diagnosis of *Echinococcus granulosus* infection in dogs by amplification of a newly identified repeated DNA sequence. Am J Trop Med Hyg. 2003;69(3):324-30.
25. Laurimaa L, Davison J, Plumer L, Süld K, Oja R, Moks E, et al. Noninvasive detection of *Echinococcus multilocularis* tapeworm in urban area, Estonia. Emerg Infect Dis. 2015;21(1):163-4.
26. Malecela MN, Ducker C. A road map for neglected tropical diseases 2021-2030. Trans R Soc Trop Med Hyg. 2021;115(2):121-3.
27. Galeh TM, Spotin A, Mahami-Oskouei M, Carmena D, Rahimi MT, Barac A, et al. The seroprevalence rate and population genetic structure of human cystic echinococcosis in the Middle East: A systematic review and meta-analysis. Int J Surg. 2018;51:39-48.
28. Buishi IE, Njoroge EM, Bouamra O, Craig PS. Canine echinococcosis in northwest Libya: assessment of coproantigen ELISA, and a survey of infection with analysis of risk-factors. Vet Parasitol. 2005;130(3-4):223-32.
29. Moro PL, Lopera L, Bonifacio N, Gonzales A, Gilman RH, Moro MH. Risk factors for canine echinococcosis in an endemic area of Peru. Vet Parasitol. 2005;130(1-2):99-104.
30. Torgerson PR, Heath DD. Transmission dynamics and control options for *Echinococcus granulosus*. Parasitology. 2003;127 Suppl:S143-58.
31. Cabrera PA, Parietti S, Haran G, Benavidez U, Lloyd S, Perera G, et al. Rates of reinfection with E*chinococcus granulosus*, *Taenia hydatigena*, *Taenia ovis* and other cestodes in a rural dog population in Uruguay. Int J Parasitol. 1996;26(1):79-83.
32. Moss JE, Chen X, Li T, Qiu J, Wang Q, Giraudoux P, et al. Reinfection studies of canine echinococcosis and role of dogs in transmission of *Echinococcus multilocularis* in Tibetan communities, Sichuan, China. Parasitology. 2013;140(13):1685-92.
33. Craig P, Mastin A, van Kesteren F, Boufana B. *Echinococcus granulosus*: Epidemiology and state-of-the-art of diagnostics in animals. Vet Parasitol. 2015;213(3-4):132-48.

34. Oba P, Ejobi F, Omadang L, Chamai M, Okwi AL, Othieno E, et al. Prevalence and risk factors of *Echinococcus granulosus* infection in dogs in Moroto and Bukedea districts in Uganda. Trop Anim Health Prod. 2016;48(2):249-54.
35. Yuan R, Wu H, Zeng H, Liu P, Xu Q, Gao L, et al. Prevalence of and risk factors for cystic echinococcosis among herding families in five provinces in western China: a cross-sectional study. Oncotarget. 2017;8(53):91568-76.
36. Yang YR, McManus DP, Huang Y, Heath DD. *Echinococcus granulosus* infection and options for control of cystic echinococcosis in Tibetan communities of Western Sichuan Province, China. PLoS Negl Trop Dis. 2009;3(4):e426.
37. Lashkarizadeh MR, Hooshmand N, Nasibi S, Mohammadi MA, Shamsaddini S, Kamyabi H, et al. Genetic Profile of Hydatid Cysts in Patients with Multi-Organ Involvement: Mixed Infections by Different Strains. Vector Borne Zoonotic Dis. 2019;19(10):724-30.
38. Mirbadie SR, Kamyabi H, Mohammadi MA, Shamsaddini S, Harandi MF. Copro-PCR prevalence of *Echinococcus granulosus* infection in dogs in Kerman, south-eastern Iran. J Helminthol. 2018;92(1):17-21.
39. Bowles J, Blair D, McManus DP. Genetic variants within the genus *Echinococcus* identified by mitochondrial DNA sequencing. Mol Biochem Parasitol. 1992;54(2):165-73.
40. Wang Q, Yu WJ, Zhong B, Shang JY, Huang L, Mastin A, et al. Seasonal pattern of *Echinococcus* re-infection in owned dogs in Tibetan communities of Sichuan, China and its implications for control. Infect Dis Poverty. 2016;5(1):60.
41. Lahmar S, Lahmar S, Boufana B, Bradshaw H, Craig PS. Screening for *Echinococcus granulosus* in dogs: Comparison between arecoline purgation, coproELISA and coproPCR with necropsy in pre-patent infections. Vet Parasitol. 2007;144(3-4):287-92.

Table 1. The primer sequences used in the present Study for the diagnosis of cystic canine echinococcosis in Semnan province, Iran.

Primer	Name	Sequence (5´> 3´)	Length	Genetic marker	Reference
Forward (F)	JB3	TTTTTTGGGCATCCTGAGGTTTAT	24	Cox1	**(39)**
Reverse (R)	JB4.5	TAAAGAAAGAACATAATGAAAATG	24		
Forward (F)	Eg1121a	GAATGCAAGCAGCAGATG	18	133-bp tandem repeat unit	**(24)**
Reverse (R)	Eg1122a	GAGATGAGTGAGAAGGAGTG	20		
Forward (F)	EGfor1	GTGTGTTACATTAATAAGGGTG	22	SSU-rDNA	**(25)**
Reverse (R)	EGrev1	CTTGTTACGACTTACCTCAA	20		

Table 2. The frequency of Taeniidae / *Echinococcus granulosus sensu lato* eggs in the owned dogs of Semnan province, Iran, before anthelmintic treatment, using microscopy and copro-PCR techniques.

	Study areas	No. of fecal samples examined	Type of dogs		Microscopic observation	Copro-PCR
			Sheepdog	Guard dog	No. of positive samples, taeniidae eggs (%)	No. of positive samples, E. granulosus sensu lato (%)
Central Iran	Mayamey	43	18	25	7 (16.3%)	4 (9.3%)
	Shahrood	119	46	73	14 (11.8%)	9 (7.5%)
	Damghan	97	38	59	11 (11.3%)	7 (7.2%)
	Semnan	64	24	40	6 (9.4%)	4 (6.2%)
	Garmsar	58	25	33	10 (17.2%)	7 (12%)
	**Total**	**381**	**151**	**230**	**48 (12.6%)**	**31 (8.1%)**

Table 3: Survival rate estimates among dogs reinfected with *Echinococcus granulosus sensu lato* in Semnan province, Central Iran.

Time post-treatment (month)	All dogs, % (95% CI)	Guard dogs, % (95% CI)	Sheepdogs, % (95% CI)
**3**	90.4 (87.5, 93.5)	92.0 (88.5, 95.7)	88.3 (83.5, 93.4)
**6**	89.3 (86.2, 92.5)	91.5 (87.8, 95.4)	86.4 (81.3, 91.8)
**9**	84.4 (80.8, 88.2)	88.4 (84.1, 92.9)	79.4 (73.3, 85.9)
**12**	77.5 (73.2, 82.0)	83.3 (78.2, 88.7)	70.2 (63.3, 77.9)
**15**	66.8 (61.6, 72.3)	75.4 (69.2, 82.2)	55.8 (47.9, 65.0)

634 **Table 4:** Cox hazards regression analysis to assess the risk of echinococcosis reinfection in the owned dogs in rural areas of Central Iran.

Variable	Category	Hazard Ratio (HR)	95% CI	P-value
**Gender**	Female	Reference		
	Male	1.47	0.76, 2.85	0.25
**Age**	Adult	Reference		
	Pup / Young	0.94	0.55, 1.61	0.82
**Dog type**	Guard dog	Reference		
	Sheepdog	1.81	1.22, 2.68	0.003
**Roaming behaviour**	Always leashed	Reference		
	Free at Night	1.00	0.57, 1.74	0.99
	Always free	0.85	0.52, 1.38	0.51
**Feeding habit**	Human food	Reference		
	Viscera	2.01	1.30, 3.08	0.001
**Anthelmintic treatment**	No	Reference		
	Yes	0.50	0.27, 0.96	0.03
**Study area**	Shahroud	Reference		
	Mayamey	1.37	0.73, 2.61	0.32
	Damghan	0.99	0.58, 1.69	0.99
	Semnan	0.92	0.50, 1.69	0.79

	Garmsar	1.45	0.82, 2.55	0.19
**Home slaughter**	No	Reference		
	Yes	2.02	1.28, 3.18	0.002

Table 5. Summary of the findings of reinfection studies on canine echinococcosis conducted in different endemic countries.

Country	No. samples	Host	Months post treatment, (reinfection rate, %)	Method	Reference
**Iran**	381	owned dogs	3 (9.6%)<br>6 (10.7%)<br>9 (15.6%)<br>12 (22.5%)<br>15 (33.2%)	Copro-PCR	Present study
**Iran**	150	owned dogs	2 (8)<br>5 (12)<br>12 (17)	Copro-PCR	(20)
**Morocco**	595	owned dogs	monthly rate 4%	Copro-PCR	(12)
**China (Shiqu county)**	584	owned dogs	3 (13.36)<br>6 (5.8)<br>9 (0)<br>12(1.56)	Copro-ELISA	(40)
**China (Seda county)**	584	owned dogs	3 (12.5)<br>6 (3.13)<br>9 (0)<br>12(1.56)	Copro-ELISA	(40)
**China**	281	owned dogs	2 (9.6)<br>5 (3.1)<br>12 (11)	Copro-PCR<br>Copro-ELISA	(32)
**Tunisia**	375	semi-stray dogs	2 (6.45)<br>4 (3.57)<br>8 (6.25)<br>12 (16.66)	Flotation	(41)
**Libya**	334	owned dogs	15 (22.2)	Copro-antigen	(28)

**China**	50	owned dogs	3 (25)<br>7 (29.2)<br>12 (16.7)	Copro-antigen	(21)
**Uruguay**	367	ND	2 (0)<br>4 (6.8)<br>8 (18.6)<br>12(27.9)	ND	(31)

ND: No data

Figure 1. Distribution of the sampled farms in Semnan province, Iran, showing 290 farms to assess *Echinococcus granulosus sensu lato* reinfection in 381 dogs. The map is produced by ESRI's ArcGIS Desktop ver. 10.8.2 using the shapefile available online from https://data.humdata.org/dataset/09c2c869-44c5-4e92-8252-d1215b4e85c8.

Figure 2. Field procedures and initial microscopic diagnosis (A–B) Representative sheepdogs, illustrating the typical definitive host population for *Echinococcus granulosus* sensu lato in the endemic areas of Garmsar (A) and Semnan (B) districts. (C) Field collection of fresh fecal sample from a guard dog in Shahroud district. (D) Microscopic visualization of a characteristic taeniid egg detected in a canine fecal sample during primary parasitological examination. These figures were created by the authors and is published under CC BY 4.0.

Figure 3. Kaplan-Meier Survival rate of the owned dogs reinfected with *Echinococcus granulosus sensu lato* . The cumulative survival rate stratified by sex (A), age (B), and dog type (C).

Supporting Information file 1. Data file showing all owned dogs included in the 15-month follow-up study after praziquantel treatment, containing individual characteristics, diagnostic results at each sampling time point, and reinfection outcomes used for the analyses.

Figure 1

Click here to access/download;Figure;Figure 1.tif

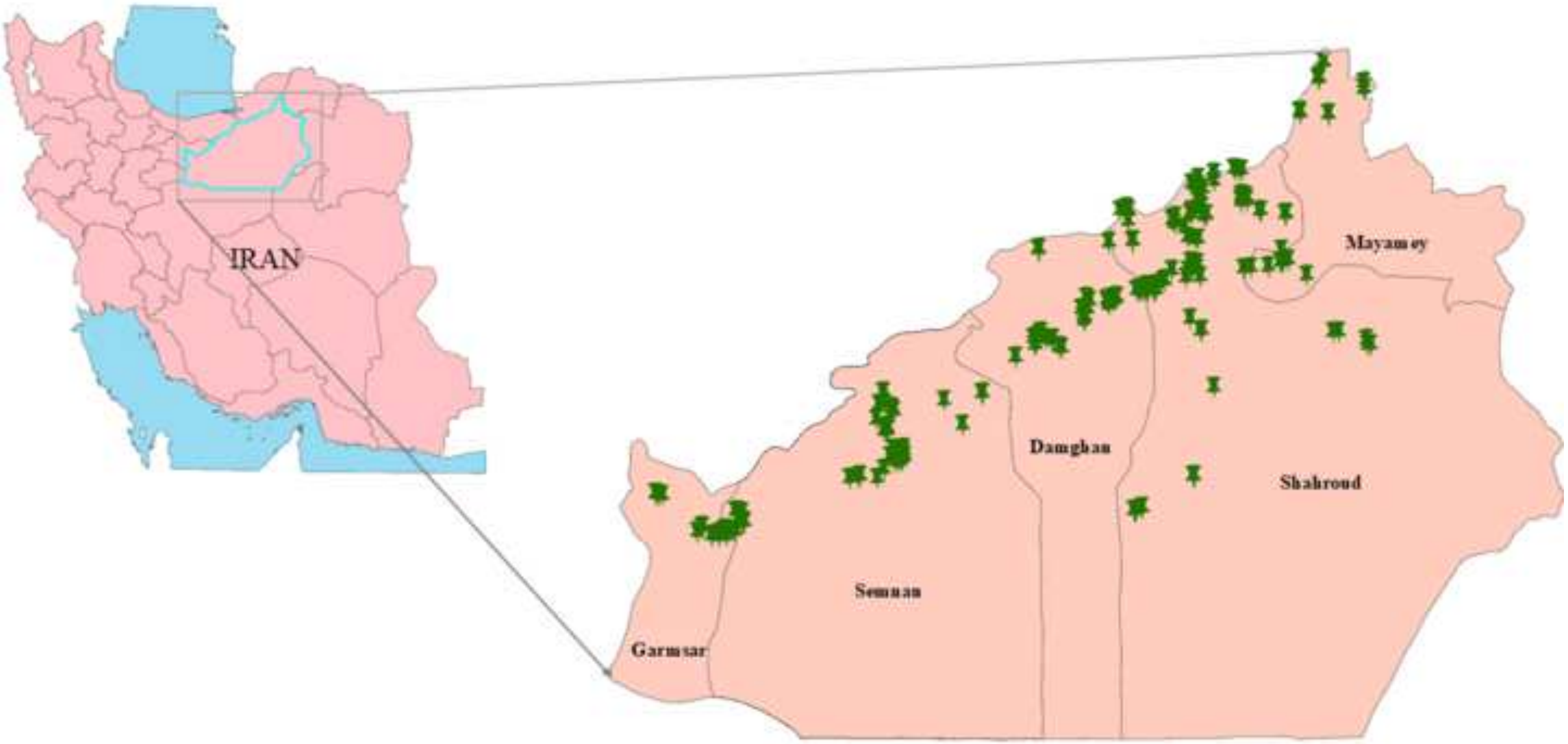

Figure 2 Click here to access/download;Figure;Figure 2.tif

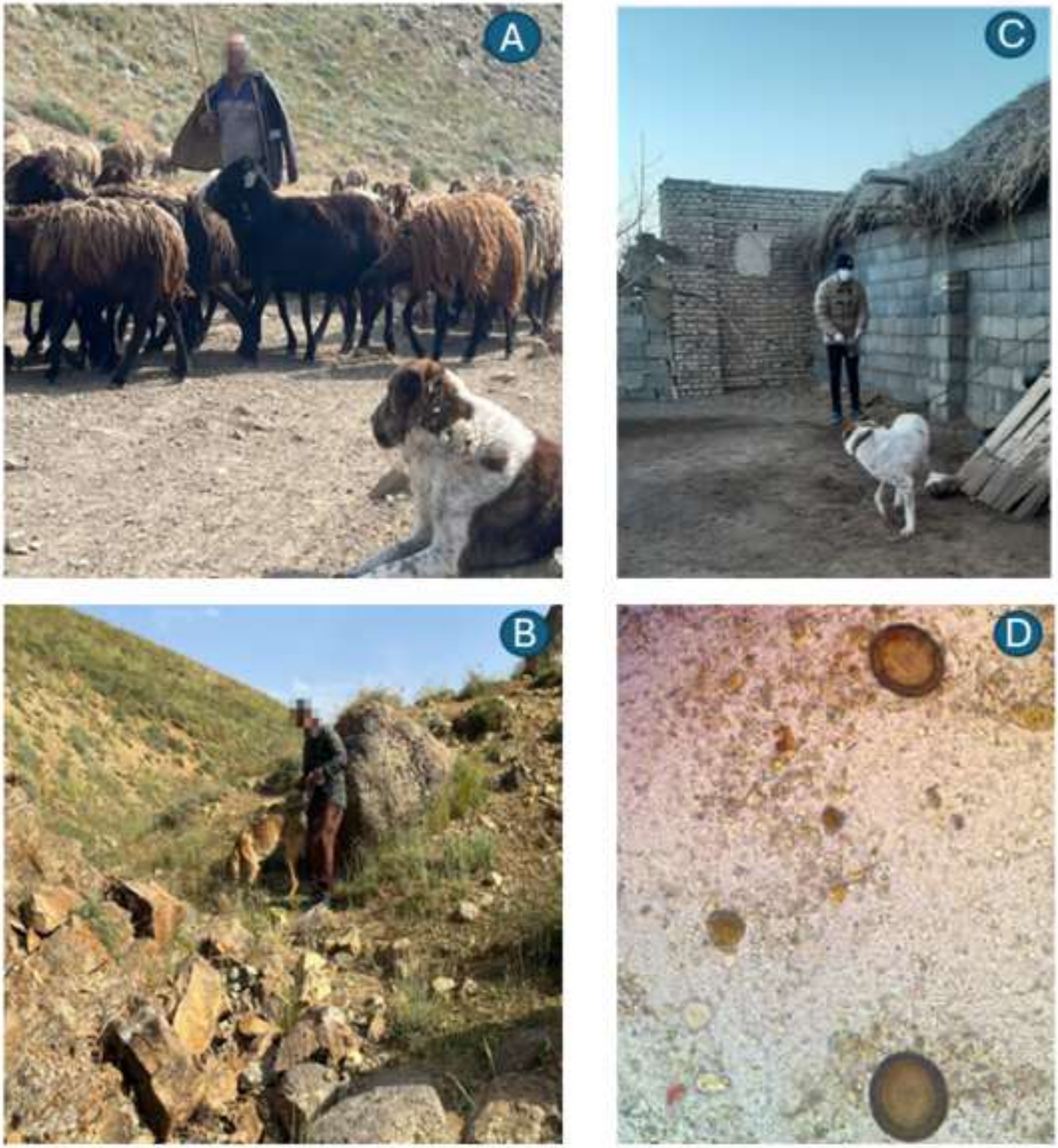

Figure_3 Click here to access/download;Figure;Figure_3.jpg

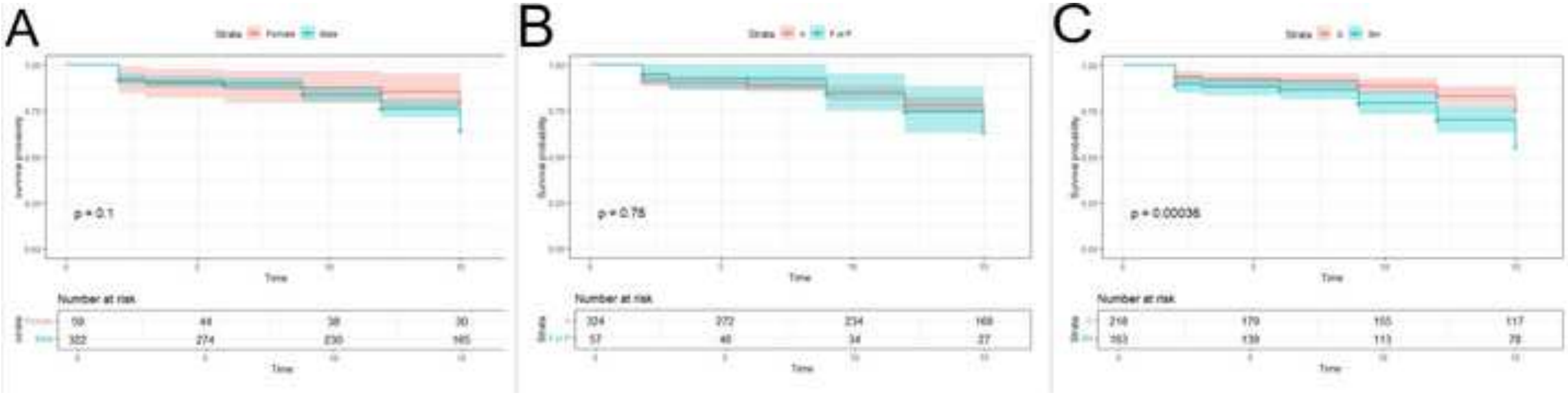

Supporting Information file 1

Click here to access/download

Supporting Information

Re_infection_Data_Echino_14041007.xlsx

Dear Dr. Eduardo José Lopes-Torres

Academic Editor, PLoS Neglected Tropical Diseases

Many thanks for reviewing our manuscript (PNTD-D-25-01258) entitled "Reinfection dynamics of owned dogs with *Echinococcus granulosus* in endemic rural areas of central Iran: Implications for cystic echinococcosis control". All the comments of the reviewers are considered, and the manuscript has been modified accordingly. Please find the point-by-point modifications below (highlighted). Thank you.

Best regards

Majid Fasihi Harandi and Mohammad Fallah, the corresponding authors

Correspondences: majid.fasihi@gmail.com ; mohfall@yahoo.com

Reviewer 1

Overall, the study demonstrates methodological rigor and satisfactorily meets the main criteria for acceptance:

Objectives and Hypothesis

Comments. The study objectives are clearly articulated, with a well-defined and testable hypothesis. The rationale is presented in a way that highlights the relevance and significance of the research.

Study Design

The chosen design is appropriate to address the stated objectives. The methodological framework is well-structured, ensuring consistent and relevant data collection.

Study Population

The study population is clearly described and appropriate for testing the proposed hypothesis.

Sample Size

The sample size is sufficient to provide adequate statistical power, strengthening the robustness and reliability of the findings.

Statistical Analyses

The statistical methods applied are appropriate and correctly used, consistently supporting the study's conclusions. Data handling is transparent and well-grounded.

Ethical and Regulatory Considerations

No concerns arise regarding ethical or regulatory compliance. The study adheres to good research practices and ethical standards.

In summary, the study presents solid methodological quality, appropriate analyses, and full ethical compliance, placing it in a favorable position for acceptance.

Response. We thank you for your careful assessment. Thanks very much for your positive feedback and constructive evaluation of our manuscript.

Comment. The analysis presented is consistent with the original analysis plan, demonstrating methodological coherence throughout the study. The results are reported in a clear and comprehensive manner, ensuring that readers can easily understand the findings and their significance. Furthermore, the figures, including tables and images, are of good quality, effectively enhancing the clarity of the data presentation and supporting the interpretation of the results.

Response. We sincerely thank the reviewer for confirming the methodological coherence, the reporting of the results.

Comment. The conclusions are well supported by the data presented, reflecting the strength and consistency of the findings. The limitations of the analysis are clearly acknowledged, demonstrating transparency and strengthening the overall credibility of the study. The authors thoughtfully discuss how the data contribute to advancing knowledge on the topic, highlighting the study's relevance within the broader scientific context. Moreover, the public health implications are explicitly addressed, underscoring the significance of the research in guiding practice and informing future initiatives.

Response. Thank you for your constructive review.

Reviewer #1: A minor revision is recommended, as the manuscript shows potential to enrich the scientific debate and contribute to a better understanding of Echinococcus granulosus sensu lato reinfection in dogs from rural areas.

Comment 1. One of the most important recommendations I would like to highlight is the standardization of the scientific name currently used for Echinococcus granulosus. When referring to all genetic variants of the species, the correct terminology is Echinococcus granulosus sensu lato (Vuitton et al., 2020). Throughout the manuscript, including the title, the use of this term is inconsistent. Sometimes the correct terminology is used, while at other times, only Echinococcus granulosus appears. I recommend a careful review of the nomenclature to ensure uniformity.

Response. Thank you, Amended. All the scientific names were modified according to the reviewer comment.

Comment 2. Regarding the methodology for collecting fecal samples from herding dogs: how can one be certain that the feces collected at the resting places truly belong to the

intended dog, considering these animals are free to roam the property? A more detailed explanation of this procedure would be helpful.

Response. Most samples from herding dogs were collected from their resting areas. Shepherds and dog owners were asked to collect fecal samples from each dog immediately after defecation. Some dogs were also restrained or tied up until defecation and sample collection. The statements regarding this issue were modified according to the reviewer's comments (lines 122-125).

Comment 3. Another relevant question relates to the samples analyzed by molecular biology. Out of the 31 samples that tested positive for Echinococcus granulosus sensu lato, why were only 9 sequenced? Wouldn't it be more informative to sequence all samples to better identify the genotypes involved in the study? This is especially important considering that different genotypes may behave differently during the course of infection.

Response. Thank you for this important comment. We fully agree that sequencing all positive samples would provide a more comprehensive genotype overview. However, two considerations guided our decision to sequence only nine samples. First, the primary scope of the study was to investigate potential re-infection events, not to characterize the full genetic diversity of all cases. Second, we faced significant budget limitations, as the project was conducted without dedicated funding for full-scale molecular characterization. Whole-sample sequencing of all isolates was unfortunately beyond the financial capacity of the study. However, to ensure that the sequenced data represent the overall burden of infection in the region, sample selection was performed in the whole geographical diversity of the study area.

We fully acknowledge that this represents a limitation of our study, and we have now clearly stated this in the revised manuscript. Despite this constraint, the targeted sequencing approach was adequate for addressing our main research question regarding re-infection as

the central aim of the research. The statements regarding this issue are included in the manuscript Discussion section (lines 391-397).

Comment 4. On page 4 (Introduction), third line: replace the word “cardinal” with “main.”

Response. Amended.

Comment 5. On page 4 (Introduction), line 20: please add a reference for the statement: “Dogs can become…CE transmission.”

Response. Amended.

Comment 6. On page 4 (Introduction), line 25: please add a reference for the statement: “The frequency…endemic region.”

Response. Amended.

Comment 7. On page 6 (Materials and Methods), line 7: the citation of Wikipedia as a reference is not appropriate in a scientific manuscript. Is there a more reliable bibliographic source that could be used instead? I suggest replacing this reference.

Response. Wikipedia citation was replaced in the reference list by the official government data source in the Iranian Statistical Center of Iran (SCI) ("Results of the Agricultural and Livestock Census, Semnan Province") (line 115).

Comment 8. On page 7 (Materials and Methods), line 21: where it reads “for identification of the dogs,” I suggest rephrasing to: “for identification of dogs during…”

Response. The phrase has been modified.

Comment 9. In the title of Table 1, I recommend rephrasing to: “Study for the diagnosis of cystic canine echinococcosis...”

Response. Thank you, amended.

Reviewer #2

Summary and General Comments

The study investigates reinfection by Echinococcus granulosus sensu lato in herding and guard dogs following treatment with praziquantel. Fecal samples from 381 dogs in rural areas of Iran were analyzed, with 31 samples testing positive for Echinococcus eggs. After chemotherapy, fecal samples from the same animals were collected at different time points to assess reinfection. The results indicate that the likelihood of reinfection increased with time after treatment. Furthermore, herding dogs exhibited the highest reinfection rates, along with various risk factors contributing to reinfection.

Through molecular biology analysis, the presence of Echinococcus sensu stricto and Echinococcus canadensis was confirmed in some samples. Therefore, this study is original and provides updated data and evidence that support efforts to control cystic echinococcosis in countries facing this zoonotic disease.

Objectives and Hypothesis

Comment 1. The manuscript is interesting specially to surveys the infection of E. granulosus in dogs, a zoonotic helminth. The idea of the manuscript is audacious to considers microscopy and molecular analysis. Although morphological analyses of eggs found in feces require a specialist to identify the eggs or even have the limitation do not identify in the species level, and genetic analysis can be used as a tool to complement integrative taxonomy, integrating morphological and molecular data to provide complete information. I suggest that the authors include images from parasitological examination of feces, with data from eggs identified by morphology, as well as morphometry. In addition, it is possible to compare how efficient the PCR methodology is for evaluating helminth infection.

Response. Thanks for this comment. We appreciate your valuable and constructive comments. In accordance with your suggestion, we have now included representative images from the parasitological examination of dog feces, including image of the taeniid eggs identified by morphology. To provide visual context for the fieldwork, additional field images are added to show dog fecal sampling and the study area with shepherd and guard dogs (lines 628-633). Because the majority of the examined eggs displayed classic *Taeniidae* morphology, morphometry was performed only in cases where egg morphology was not clearly characteristic of *Taeniidae*. For samples with typical and unambiguous morphological features, additional morphometric analysis was not considered necessary.

Regarding the comparative efficiency of PCR methodologies for evaluating canine echinococcosis, this comparison is beyond the scope of the present study, which was specifically designed to investigate re-infection events rather than to evaluate molecular diagnostic techniques. Our PCR analyses were used solely as supportive tools not as part of a methodological performance assessment.

Comment 2. Introduction: I suggest reviewing the introduction and try to be more objective, showing the importance of your work.

Response. The Introduction section is reviewed once again and some statements and modifications were made to show the importance of the study (lines 63-67, 71-77 and 80-82).

Study Design

Comment 3. The hypothesis of the study was not clear in the text especially in introduction.

Response. We thank the reviewer for this comment. We have now clarified the study hypothesis in the Introduction and underlying research rationale (Line 92-97).

Study Population

Comment 4. The dising are appropriate.

Response. Thanks.

Sample Size and Statistical Analyses

The size of sample was adequate to test the objetctive of the study, as weel as the statisc analysis.

Response. Thanks.

Methodology:

Comment 5. I suggest changing the map and highlight the study localities, as well as distinguish in the map the dogs. Other suggestions are in the text.

Response. Thank you for this helpful suggestion. The map has now been revised to clearly highlight the study localities, and the sampled farms are indicated on the updated figure.

Results

Comment 6. The analysis match the plan. The results are ok however needs major resivion the fugures are not with sufficient quality

Response. We thank the reviewer for the comment regarding figure quality. We have done our best to provide high-resolution images, but it is possible that the quality was reduced during PDF conversion by the submission system. To ensure proper evaluation, we kindly ask the reviewer to click on the link in the top right corner of the figure page to download the original high-quality version of the images. Please let us know if further adjustments are needed.

Conclusions

The journal's proposals are subject to reviewer approval.

Discussion:

Well made and highlight the importance of the work.

Comment 1. I suggest to include the complete cycle of the Echinococcus showing the dog as key for transmission.

Response. We greatly appreciate your insightful and constructive comments, which have significantly strengthened the manuscript. A paragraph providing a complete explanation of the life cycle of E. granulosus was included in the text. It fully explains the complete stages of the cycle including: definitive host, intermediate hosts, and transmission of infection. (line 232-241)

Comment 2. Please indicate the map figure here, it's easier for the reader.

Response. Amended.

Comment 3. I suggest that the counties be more highlighted on the map, making it easier for the reader.

Response. Amended.

Comment 4. How many grams did you collect and analyze?

Response. Thanks, the weight of the stool sample is provided in the text (line 126-127).

Comment 5. How the taeniid eggs were collected? Please describe this method:

Response. The collection of taeniid eggs was performed using the sugar flotation method. Details of the method is amended in the Methods section of the manuscript. This density ensures that the parasite eggs float to the surface during centrifugation and are separated from the heavier debris (line 133-138).

Comment 6. After anthelmintic treatment, the authors analyzed and re-treated the dogs. Were the positive dogs treated twice or more times with PZQ before the negative result?

Response. Thank you for your attention. In this study, dogs that were positive in the initial test were first treated and then re-evaluated after a 7-day period. If the test result was still positive (which was the case for only 4 dogs), a re-treatment course with PZQ was applied and then a final sampling was performed to confirm the effectiveness of the treatment. (line 139-145).

Comment 7. The dogs whose fecal samples were collected between the months 3 and 15. Were these dogs treated in those months as well? Or did the dogs only receive the first treatment? This methodology was not very clear.

Response. As described above, the dogs that were positive in the initial test were first treated and then re-evaluated after a 7-day period. If the test result was still positive (which was the case for only 4 dogs), a re-treatment course with PZQ was applied and then a final sampling was performed to confirm the effectiveness of the treatment. (line 139-145).

Comment 8. Why did you not do phylogenetic analysis?

Response. Thanks for the comment. The amplified fragment was too short to yield meaningful or reliable phylogenetic resolution. Short partial sequences do not provide adequate informative sites for constructing well-supported phylogenetic trees, and any analysis based on such limited data would be scientifically weak and potentially misleading. For this reason, we did not include phylogenetic analysis. Also, please note that the primary aim of our study was to assess re-infection rather than to investigate phylogenetic relationships. Our methodological design, including the choice of target gene fragment, was aligned with evaluating post-treatment re-infection status and not with generating sufficient sequence data for robust phylogenetic inference.

Comment 9. I suggest highlighting the counties studied on the map and distinguishing the dogs (shepherd dogs and guard dogs) with different colors.

Response. Thank you for your valuable suggestion. In the revised version, the studied counties are clearly displayed on the map. Please note that we marked a total of 290 farms, and in many of these farms both guard dogs and sheepdogs were present.

Comment 10. I suggest showing the respective isolates found for each location and dog (sheepdogs or guard dogs). Do you believe there is a relationship?

Response. We agree that presenting the respective *E. granulosus* isolates by location and by dog category (sheepdogs vs. guard dogs) could provide additional epidemiological insights. However, this type of analysis is beyond the primary aim and scope of the present study, which was specifically designed to investigate the re-infection dynamics of canine echinococcosis following treatment. Nevertheless, we did assess the risk of reinfection by dog category using Cox proportional hazards regression. This analysis showed that sheepdogs were 1.81 times more at risk of reinfection than guard dogs, and this association was statistically significant. These findings already highlight a meaningful epidemiological difference between the two dog populations.

Comment 11. Figure 2 in the manuscript was not visible due to its size and quality of the image.

Response. We thank the reviewer for the comment regarding figure quality. We have done our best to provide high-resolution images, but it is possible that the quality was reduced during PDF conversion by the submission system. To ensure proper evaluation, we kindly ask the reviewer to click on the link in the top right corner of the figure page to download the original high-quality version of the images. Please let us know if further adjustments are needed.

Thank you.

Click here to access/download
Revised Article with Changes Highlighted
SRM_manuscript_14040920.docx
