# Supplementary material for: Reinfection dynamics of owned dogs with Echinococcus granulosus sensu lato in endemic rural areas of central Iran: Implications for cystic echinococcosis control
Source: PLoS Negl Trop Dis. 2026 Jun 29;20(6):e0014451. doi: 10.1371/journal.pntd.0014451 (PMC13327511; doi:10.1371/journal.pntd.0014451)
Supplement: Attachment — Submitted filename: Secon revision.pdf [file pntd.0014451.s010.pdf]

Reviewer Comments

The revised manuscript entitled *"Reinfection dynamics of owned dogs with Echinococcus granulosus sensu lato in endemic rural areas of central Iran: Implications for cystic echinococcosis control"* presents a relevant and scientifically valuable contribution to the understanding of canine reinfection dynamics in endemic settings. The study addresses an important gap in the epidemiology and control of cystic echinococcosis (CE), particularly regarding reinfection following praziquantel treatment in owned farm dogs.

Overall, the revised version demonstrates substantial improvement compared to the original submission. Most of the comments and concerns raised during the previous review round have been appropriately addressed.

The manuscript is methodologically sound, epidemiologically relevant, and provides important longitudinal data that may support future CE control strategies in endemic regions.

Major Revisions Addressed

1. **Nomenclature standardization**

The authors have substantially improved the consistency of the nomenclature throughout the manuscript by appropriately using *Echinococcus granulosus sensu lato (E. granulosus s. l.)* in most sections, including the title, abstract, methods, results, and discussion. This correction significantly improves taxonomic accuracy and scientific clarity.

2. **Clarification of fecal sample collection procedures**

The revised methodology now provides a clearer explanation regarding fecal sample collection from sheepdogs and guard dogs. The additional details describing owner-assisted collection immediately after defecation, sampling from resting areas, and temporary restraint of dogs considerably improve the reproducibility and reliability of the study design.

3. **Discussion regarding sequencing of only a subset of samples**

The authors adequately acknowledged the exploratory nature of the genotyping analysis and clarified that only a subset of positive samples was sequenced. Importantly, the manuscript now recognizes this as a limitation and appropriately avoids overinterpretation of genotype distribution data.

4. **Additional references and textual corrections**

The inclusion of additional references supporting statements in the Introduction improved the scientific grounding of the manuscript. Minor wording corrections and textual revisions also enhanced readability.

Scientific Merit

The study has several important strengths:

- longitudinal follow-up over a 15-month period;
- relatively large cohort of owned dogs from endemic rural communities;
- combined use of microscopy and copro-PCR diagnostics;
- application of Kaplan–Meier survival analysis and Cox proportional hazards modeling;
- identification of behavioral and management-related risk factors associated with reinfection;
- relevant discussion of One Health implications and control strategies.

The findings provide valuable epidemiological evidence supporting the need for integrated CE control measures beyond dog deworming alone.

Remaining Minor Issues

Although the manuscript has improved considerably, a few minor issues remain:

1. Some sections of the Discussion remain somewhat repetitive and could be slightly condensed to improve readability.
2. Minor grammatical inconsistencies and language polishing are still required throughout the text.
3. The use of direct website links in the “Study Area” section would ideally be replaced by properly formatted institutional or governmental references.

These issues, however, are editorial in nature and do not compromise the scientific quality of the study.

Final Recommendation

Recommendation: Minor Revision

The manuscript is scientifically sound, relevant, and suitable for publication after minor editorial revision. The study contributes important data on canine reinfection dynamics in endemic rural settings and offers valuable insights for the design of sustainable cystic echinococcosis control programs.
