# Supplementary material for: Reinfection dynamics of owned dogs with Echinococcus granulosus sensu lato in endemic rural areas of central Iran: Implications for cystic echinococcosis control
Source: PLoS Negl Trop Dis. 2026 Jun 29;20(6):e0014451. doi: 10.1371/journal.pntd.0014451 (PMC13327511; doi:10.1371/journal.pntd.0014451)
Supplement: Attachment — Submitted filename: PNTD_SRM_Response_14040507.docx [file pntd.0014451.s002.docx]

**Dear Editor-in-Chief, PLoS NTD**

We appreciate your feedback regarding the manuscript PNTD-D-25-01258 entitled "Reinfection dynamics of owned dogs with Echinococcus granulosus in endemic rural areas of central Iran: Implications for cystic echinococcosis control”. The manuscript is revised based on the editorial comments, with the revised sections highlighted in the main text.

**Thank you and best regards**

**M. Fasihi Harandi & M. Fallah, the Corresponding authors**

**Additional Editor Comments (if provided):**

**Comment.** While the subject area is within the remit of the PLoS NTD journal, the manuscript is not currently in a suitable format for the journal. In particular the results section is very short and should be expanded. I recommend the authors make these changes, expanding the results section in line with a research article rather than a short communication, and resubmit the manuscript for review.

**Response.** Thanks for the comment. The findings of the study are expanded both in the Abstract and the Results sections. More data are provided in these sections. The Results section is expanded and the findings are detailed in three different subheadings, including Study Population, Baseline Prevalence and genotyping, Reinfection Dynamics and Survival Analysis, and Risk Factor Analysis for Reinfection.

Thanks for your kind attention.
