# Supplementary material for: Reinfection dynamics of owned dogs with Echinococcus granulosus sensu lato in endemic rural areas of central Iran: Implications for cystic echinococcosis control
Source: PLoS Negl Trop Dis. 2026 Jun 29;20(6):e0014451. doi: 10.1371/journal.pntd.0014451 (PMC13327511; doi:10.1371/journal.pntd.0014451)
Supplement: Attachment — Submitted filename: Plos revision manuscript.docx [file pntd.0014451.s004.docx]

**Plos Review Manuscript**

Title: Reinfection dynamics of owned dogs with *Echinococcus granulosus* in endemic rural areas of central Iran: Implications for cystic echinococcosis control

The manuscript is interesting specially to surveys the infection of *E. granulosus* in dogs, a zoonotic helminth. The idea of the manuscript is audacious to considers microscopy and molecular analysis. Although morphological analyses of eggs found in feces require a specialist to identify the eggs or even have the limitation do not identify in the species level, and genetic analysis can be used as a tool to complement integrative taxonomy, integrating morphological and molecular data to provide complete information. I suggest that the authors include images from parasitological examination of feces, with data from eggs identified by morphology, as well as morphometry. In addition, it is possible to compare how efficient the PCR methodology is for evaluating helminth infection. Therefore, the results of reinfection are interesting especially to understand the dynamics of the transmission cycle and highlight the importance of domestic dogs, even when treated.

Introduction: I suggest reviewing the introduction and try to be more objective, showing the importance of your work.

I suggest describing the cycle for the reader understand how dogs are important to CE transmission.

The questions of the paper would be clearer and objective, as well the aim of the study.

Methodology:

I suggest changing the map and highlight the study localities, as well as distinguish in the map the dogs. Other suggestions are in the text.

Results: I made some suggestion in the text.

Discussion: Well made and highlight the importance of the work.
