# Supplementary material for: Reinfection dynamics of owned dogs with Echinococcus granulosus sensu lato in endemic rural areas of central Iran: Implications for cystic echinococcosis control
Source: PLoS Negl Trop Dis. 2026 Jun 29;20(6):e0014451. doi: 10.1371/journal.pntd.0014451 (PMC13327511; doi:10.1371/journal.pntd.0014451)
Supplement: Attachment — Submitted filename: Reviewers_Response_PNTD_14040920.docx [file pntd.0014451.s006.docx]

**Dear Dr. Eduardo José Lopes-Torres**

**Academic Editor, PLoS Neglected Tropical Diseases**

Many thanks for reviewing our manuscript (PNTD-D-25-01258) entitled “Reinfection dynamics of owned dogs with *Echinococcus granulosus* in endemic rural areas of central Iran: Implications for cystic echinococcosis control”. All the comments of the reviewers are considered, and the manuscript has been modified accordingly. Please find the point-by-point modifications below (highlighted). Thank you.

**Best regards**

**Majid Fasihi Harandi and Mohammad Fallah, the corresponding authors**

Correspondences: majid.fasihi@gmail.com ; mohfall@yahoo.com

**Reviewer 1**

Overall, the study demonstrates methodological rigor and satisfactorily meets the main criteria for acceptance:

Objectives and Hypothesis

**Comments.** The study objectives are clearly articulated, with a well-defined and testable hypothesis. The rationale is presented in a way that highlights the relevance and significance of the research.

**Study Design**

The chosen design is appropriate to address the stated objectives. The methodological framework is well-structured, ensuring consistent and relevant data collection.

**Study Population**

The study population is clearly described and appropriate for testing the proposed hypothesis.

**Sample Size**

The sample size is sufficient to provide adequate statistical power, strengthening the robustness and reliability of the findings.

**Statistical Analyses**

The statistical methods applied are appropriate and correctly used, consistently supporting the study’s conclusions. Data handling is transparent and well-grounded.

**Ethical and Regulatory Considerations**

No concerns arise regarding ethical or regulatory compliance. The study adheres to good research practices and ethical standards.

In summary, the study presents solid methodological quality, appropriate analyses, and full ethical compliance, placing it in a favorable position for acceptance.

**Response.** We thank you for your careful assessment. Thanks very much for your positive feedback and constructive evaluation of our manuscript.

**Comment.** The analysis presented is consistent with the original analysis plan, demonstrating methodological coherence throughout the study. The results are reported in a clear and comprehensive manner, ensuring that readers can easily understand the findings and their significance. Furthermore, the figures, including tables and images, are of good quality, effectively enhancing the clarity of the data presentation and supporting the interpretation of the results.

**Response**. We sincerely thank the reviewer for confirming the methodological coherence, the reporting of the results.

**Comment.** The conclusions are well supported by the data presented, reflecting the strength and consistency of the findings. The limitations of the analysis are clearly acknowledged, demonstrating transparency and strengthening the overall credibility of the study. The authors thoughtfully discuss how the data contribute to advancing knowledge on the topic, highlighting the study’s relevance within the broader scientific context. Moreover, the public health implications are explicitly addressed, underscoring the significance of the research in guiding practice and informing future initiatives.

**Response**. Thank you for your constructive review.

**Reviewer #1:** A minor revision is recommended, as the manuscript shows potential to enrich the scientific debate and contribute to a better understanding of Echinococcus granulosus sensu lato reinfection in dogs from rural areas.

**Comment 1.** One of the most important recommendations I would like to highlight is the standardization of the scientific name currently used for Echinococcus granulosus. When referring to all genetic variants of the species, the correct terminology is Echinococcus granulosus sensu lato (Vuitton et al., 2020). Throughout the manuscript, including the title, the use of this term is inconsistent. Sometimes the correct terminology is used, while at other times, only Echinococcus granulosus appears. I recommend a careful review of the nomenclature to ensure uniformity.

**Response.** Thank you, Amended. All the scientific names were modified according to the reviewer comment.

**Comment** **2.** Regarding the methodology for collecting fecal samples from herding dogs: how can one be certain that the feces collected at the resting places truly belong to the intended dog, considering these animals are free to roam the property? A more detailed explanation of this procedure would be helpful.

**Response.** Most samples from herding dogs were collected from their resting areas. Shepherds and dog owners were asked to collect fecal samples from each dog immediately after defecation. Some dogs were also restrained or tied up until defecation and sample collection. The statements regarding this issue were modified according to the reviewer's comments (lines 122-125).

**Comment 3.** Another relevant question relates to the samples analyzed by molecular biology. Out of the 31 samples that tested positive for Echinococcus granulosus sensu lato, why were only 9 sequenced? Wouldn’t it be more informative to sequence all samples to better identify the genotypes involved in the study? This is especially important considering that different genotypes may behave differently during the course of infection.

**Response.** Thank you for this important comment. We fully agree that sequencing all positive samples would provide a more comprehensive genotype overview. However, two considerations guided our decision to sequence only nine samples. First, the primary scope of the study was to investigate potential re-infection events, not to characterize the full genetic diversity of all cases. Second, we faced significant budget limitations, as the project was conducted without dedicated funding for full-scale molecular characterization. Whole-sample sequencing of all isolates was unfortunately beyond the financial capacity of the study. However, to ensure that the sequenced data represent the overall burden of infection in the region, sample selection was performed in the whole geographical diversity of the study area.

We fully acknowledge that this represents a limitation of our study, and we have now clearly stated this in the revised manuscript. Despite this constraint, the targeted sequencing approach was adequate for addressing our main research question regarding re-infection as the central aim of the research. The statements regarding this issue are included in the manuscript Discussion section (lines 391-397).

**Comment 4.** On page 4 (Introduction), third line: replace the word “cardinal” with “main.”

**Response.** Amended.

**Comment 5.** On page 4 (Introduction), line 20: please add a reference for the statement: “Dogs can become…CE transmission.”

**Response.** Amended.

**Comment 6.** On page 4 (Introduction), line 25: please add a reference for the statement: “The frequency…endemic region.”

**Response.** Amended.

**Comment 7.** On page 6 (Materials and Methods), line 7: the citation of Wikipedia as a reference is not appropriate in a scientific manuscript. Is there a more reliable bibliographic source that could be used instead? I suggest replacing this reference.

**Response.** Wikipedia citation was replaced in the reference list by the official government data source in the Iranian Statistical Center of Iran (SCI) ("Results of the Agricultural and Livestock Census, Semnan Province") ( line 115).

**Comment 8.** On page 7 (Materials and Methods), line 21: where it reads “for identification of the dogs,” I suggest rephrasing to: “for identification of dogs during…”

**Response.** The phrase has been modified.

**Comment 9**. In the title of Table 1, I recommend rephrasing to: “Study for the diagnosis of cystic canine echinococcosis...”

**Response.** Thank you, amended.

**Reviewer #2**

**Summary and General Comments**

The study investigates reinfection by Echinococcus granulosus sensu lato in herding and guard dogs following treatment with praziquantel. Fecal samples from 381 dogs in rural areas of Iran were analyzed, with 31 samples testing positive for Echinococcus eggs. After chemotherapy, fecal samples from the same animals were collected at different time points to assess reinfection. The results indicate that the likelihood of reinfection increased with time after treatment. Furthermore, herding dogs exhibited the highest reinfection rates, along with various risk factors contributing to reinfection.
Through molecular biology analysis, the presence of Echinococcus sensu stricto and Echinococcus canadensis was confirmed in some samples. Therefore, this study is original and provides updated data and evidence that support efforts to control cystic echinococcosis in countries facing this zoonotic disease.

**Objectives and Hypothesis**

**Comment 1.** The manuscript is interesting specially to surveys the infection of E. granulosus in dogs, a zoonotic helminth. The idea of the manuscript is audacious to considers microscopy and molecular analysis. Although morphological analyses of eggs found in feces require a specialist to identify the eggs or even have the limitation do not identify in the species level, and genetic analysis can be used as a tool to complement integrative taxonomy, integrating morphological and molecular data to provide complete information. I suggest that the authors include images from parasitological examination of feces, with data from eggs identified by morphology, as well as morphometry. In addition, it is possible to compare how efficient the PCR methodology is for evaluating helminth infection.

**Response.** Thanks for this comment. We appreciate your valuable and constructive comments. In accordance with your suggestion, we have now included representative images from the parasitological examination of dog feces, including image of the taeniid eggs identified by morphology. To provide visual context for the fieldwork, additional field images are added to show dog fecal sampling and the study area with shepherd and guard dogs (lines 628-633). Because the majority of the examined eggs displayed classic *Taeniidae* morphology, morphometry was performed only in cases where egg morphology was not clearly characteristic of *Taeniidae*. For samples with typical and unambiguous morphological features, additional morphometric analysis was not considered necessary.

Regarding the comparative efficiency of PCR methodologies for evaluating canine echinococcosis, this comparison is beyond the scope of the present study, which was specifically designed to investigate re-infection events rather than to evaluate molecular diagnostic techniques. Our PCR analyses were used solely as supportive tools not as part of a methodological performance assessment.

**Comment 2.** Introduction: I suggest reviewing the introduction and try to be more objective, showing the importance of your work.

**Response.** The Introduction section is reviewed once again and some statements and modifications were made to show the importance of the study (lines 63-67, 71-77 and 80-82).

**Study Design**

**Comment 3.** The hypothesis of the study was not clear in the text especially in introduction.

**Response**. We thank the reviewer for this comment. We have now clarified the study hypothesis in the Introduction and underlying research rationale (Line 92-97).

**Study Population**

**Comment 4.** The dising are appropriate.

**Response.** Thanks.

**Sample Size and Statistical Analyses**

The size of sample was adequate to test the objetctive of the study, as weel as the statisc analysis.

**Response.** Thanks.

**Methodology:**

**Comment 5.** I suggest changing the map and highlight the study localities, as well as distinguish in the map the dogs. Other suggestions are in the text.

**Response.** Thank you for this helpful suggestion. The map has now been revised to clearly highlight the study localities, and the sampled farms are indicated on the updated figure.

**Results**

**Comment 6.** The analysis match the plan. The results are ok however needs major resivion
the fugures are not with sufficient quality

**Response.** We thank the reviewer for the comment regarding figure quality. We have done our best to provide high-resolution images, but it is possible that the quality was reduced during PDF conversion by the submission system. To ensure proper evaluation, we kindly ask the reviewer to click on the link in the top right corner of the figure page to download the original high-quality version of the images. Please let us know if further adjustments are needed.

**Conclusions**

**The journal's proposals are subject to reviewer approval.**

**Discussion:**

Well made and highlight the importance of the work.

**Comment 1.** I suggest to include the complete cycle of the Echinococcus showing the dog as key for transmission.

**Response.** We greatly appreciate your insightful and constructive comments, which have significantly strengthened the manuscript. A paragraph providing a complete explanation of the life cycle of E. granulosus was included in the text. It fully explains the complete stages of the cycle including: definitive host, intermediate hosts, and transmission of infection. (line 232-241)

**Comment 2.** Please indicate the map figure here, it's easier for the reader.

**Response.** Amended.

**Comment 3.** I suggest that the counties be more highlighted on the map, making it easier for the reader.

**Response.** Amended.

**Comment 4.** How many grams did you collect and analyze?

**Response.** Thanks, the weight of the stool sample is provided in the text (line 126-127).

**Comment 5.** How the taeniid eggs were collected? Please describe this method:

**Response.** The collection of taeniid eggs was performed using the sugar flotation method. Details of the method is amended in the Methods section of the manuscript. This density ensures that the parasite eggs float to the surface during centrifugation and are separated from the heavier debris (line 133-138).

Comment 6. After anthelmintic treatment, the authors analyzed and re-treated the dogs. Were the positive dogs treated twice or more times with PZQ before the negative result?

**Response.** Thank you for your attention. In this study, dogs that were positive in the initial test were first treated and then re-evaluated after a 7-day period. If the test result was still positive (which was the case for only 4 dogs), a re-treatment course with PZQ was applied and then a final sampling was performed to confirm the effectiveness of the treatment. (line 139-145).

**Comment 7.** The dogs whose fecal samples were collected between the months 3 and 15. Were these dogs treated in those months as well? Or did the dogs only receive the first treatment? This methodology was not very clear.

**Response.** As described above, the dogs that were positive in the initial test were first treated and then re-evaluated after a 7-day period. If the test result was still positive (which was the case for only 4 dogs), a re-treatment course with PZQ was applied and then a final sampling was performed to confirm the effectiveness of the treatment. (line 139-145).

Comment 8. Why did you not do phylogenetic analysis?

**Response.** Thanks for the comment. The amplified fragment was too short to yield meaningful or reliable phylogenetic resolution. Short partial sequences do not provide adequate informative sites for constructing well-supported phylogenetic trees, and any analysis based on such limited data would be scientifically weak and potentially misleading. For this reason, we did not include phylogenetic analysis. Also, please note that the primary aim of our study was to assess re-infection rather than to investigate phylogenetic relationships. Our methodological design, including the choice of target gene fragment, was aligned with evaluating post-treatment re-infection status and not with generating sufficient sequence data for robust phylogenetic inference.

**Comment 9.** I suggest highlighting the counties studied on the map and distinguishing the dogs (shepherd dogs and guard dogs) with different colors.

**Response.** Thank you for your valuable suggestion. In the revised version, the studied counties are clearly displayed on the map. Please note that we marked a total of 290 farms, and in many of these farms both guard dogs and sheepdogs were present.

**Comment 10.** I suggest showing the respective isolates found for each location and dog (sheepdogs or guard dogs). Do you believe there is a relationship?

**Response.** We agree that presenting the respective *E. granulosus* isolates by location and by dog category (sheepdogs vs. guard dogs) could provide additional epidemiological insights. However, this type of analysis is beyond the primary aim and scope of the present study, which was specifically designed to investigate the re-infection dynamics of canine echinococcosis following treatment. Nevertheless, we did assess the risk of reinfection by dog category using Cox proportional hazards regression. This analysis showed that sheepdogs were 1.81 times more at risk of reinfection than guard dogs, and this association was statistically significant. These findings already highlight a meaningful epidemiological difference between the two dog populations.

**Comment 11.** Figure 2 in the manuscript was not visible due to its size and quality of the image.

**Response.** We thank the reviewer for the comment regarding figure quality. We have done our best to provide high-resolution images, but it is possible that the quality was reduced during PDF conversion by the submission system. To ensure proper evaluation, we kindly ask the reviewer to click on the link in the top right corner of the figure page to download the original high-quality version of the images. Please let us know if further adjustments are needed.

**Thank you.**
