# Supplementary material for: Reinfection dynamics of owned dogs with Echinococcus granulosus sensu lato in endemic rural areas of central Iran: Implications for cystic echinococcosis control
Source: PLoS Negl Trop Dis. 2026 Jun 29;20(6):e0014451. doi: 10.1371/journal.pntd.0014451 (PMC13327511; doi:10.1371/journal.pntd.0014451)
Supplement: Attachment — Submitted filename: Revision and recommendation final version.docx [file pntd.0014451.s007.docx]

**REVIEWER COMMENTS**

**Recommendation**

**Major Revision**

**Comments to the Editor (Confidential)**

This manuscript addresses an important topic for the control of cystic echinococcosis by investigating reinfection dynamics in owned dogs in an endemic rural setting. The longitudinal follow-up and field-based data collection are strengths. However, the manuscript presents limited incremental novelty relative to recent studies from the same country and overinterprets some findings beyond the observational evidence provided.

Several methodological and analytical issues require clarification, particularly regarding the definition of reinfection, potential misclassification bias, and the implementation and reporting of Cox regression assumptions. In addition, the discussion would benefit from a more balanced and proportional interpretation of the results and a clearer articulation of the study’s unique contribution.

I recommend **major revision** before the manuscript can be considered for publication.

**Comments to the Authors**

**General Assessment**

The study provides valuable longitudinal data on dog reinfection with *Echinococcus granulosus sensu lato* in an endemic rural area of Iran. The topic is relevant to both public health and veterinary epidemiology, and the follow-up period strengthens the analysis. Nevertheless, several major concerns regarding originality, methodological rigor, statistical analysis, and interpretation must be addressed to meet the standards of a high-impact journal.

**Major Comments**

**1. Originality and contribution to existing literature**

While the study is well conducted, its added value relative to recent publications—particularly studies conducted in Iran and published in the same journal—is not sufficiently articulated.

Several key findings (e.g., reinfection rates within 12–15 months, association with home slaughter and feeding of raw offal) largely confirm previously reported results. The manuscript would benefit from a clearer statement of what novel insight this study provides, whether in terms of study design, analytical approach, dog subpopulation stratification, or local transmission dynamics.

**Authors should:**

- Clearly define the specific knowledge gap addressed by this study.
- Avoid overstating novelty where findings are largely confirmatory.
- Explicitly differentiate this work from closely related recent studies.

**2. Definition and potential misclassification of reinfection**

Reinfection is defined based on coproparasitological and PCR detection following praziquantel treatment. However, the manuscript does not sufficiently address the possibility of incomplete parasite elimination or delayed egg shedding, which could lead to misclassification.

The choice of a 7-day post-treatment interval to confirm clearance requires stronger justification, as residual infection or delayed clearance cannot be entirely excluded.

**Authors should:**

- Explicitly acknowledge this limitation in the Discussion.
- Discuss the potential for misclassification bias between treatment failure and true reinfection.
- Provide stronger literature-based justification for the clearance assessment timeline.

**3. Statistical analysis and reporting**

The use of Cox proportional hazards models is appropriate; however, essential methodological details are missing.

**Specifically:**

- There is no indication that the proportional hazards assumption was tested.
- The process for variable selection in multivariable models is unclear.
- Potential collinearity between behavioral variables (e.g., home slaughter and offal feeding) is not addressed.

**Authors should:**

- Clearly state whether and how model assumptions were evaluated.
- Describe criteria for variable inclusion.
- Consider sensitivity analyses or alternative modeling approaches if assumptions are violated.

**4. Interpretation and programmatic implications**

The discussion extends to broad control strategies (e.g., integrated One Health interventions, livestock vaccination), which were not evaluated in this study.

While such strategies are relevant, the manuscript should distinguish more clearly between evidence generated by the current study and broader recommendations derived from the literature.

**Authors should:**

- Temper conclusions to align more closely with the observational data.
- Clearly separate direct findings from inferred policy implications.
- Prioritize recommendations based on feasibility and relevance to the study setting.

**5. Limited integration of genotyping results**

Although parasite genotyping is reported, the sample size is small, and the results are not analytically integrated into the main findings.

**Authors should:**

- Clarify the exploratory nature of the genotyping data.
- Avoid overinterpretation.
- Either better contextualize these results epidemiologically or reduce their emphasis.

**Minor Comments**

- The Discussion section is overly long and contains some repetition of background information.
- Greater concision and thematic organization are recommended.
- Language should be revised to remove subjective or emphatic terms (e.g., “clearly,” “obviously”).
- Ensure consistent use of *E. granulosus s.l.* throughout the manuscript.
- Some references are used redundantly and could be streamlined.

**Final Recommendation**

**Major Revision**
