# Supplementary material for: Reinfection dynamics of owned dogs with Echinococcus granulosus sensu lato in endemic rural areas of central Iran: Implications for cystic echinococcosis control
Source: PLoS Negl Trop Dis. 2026 Jun 29;20(6):e0014451. doi: 10.1371/journal.pntd.0014451 (PMC13327511; doi:10.1371/journal.pntd.0014451)
Supplement: Attachment — Submitted filename: Reviewers_Response_PNTD_14050213.docx [file pntd.0014451.s009.docx]

**Dear Dr. Eduardo José Lopes-Torres**

**Academic Editor, PLOS Neglected Tropical Diseases**

We sincerely appreciate your time and effort in re-evaluating our revised manuscript entitled “Reinfection dynamics of owned dogs with Echinococcus granulosus in endemic rural areas of central Iran: Implications for cystic echinococcosis control”. We are grateful to the reviewers for their thorough reassessment and constructive comments in this second round of review.

We have carefully considered all points raised and have revised the manuscript accordingly (highlighted).

Please find below our detailed, point-by-point responses to the reviewers’ comments.

Thank you very much for your consideration.

Best regards,

**Majid Fasihi Harandi and Mohammad Fallah,**

**the Corresponding Authors**

Reviewer #1

**1. Originality and contribution to existing literature**

**1. Originality and contribution to existing literature**

While the study is well conducted, its added value relative to recent publications—particularly studies conducted in Iran and published in the same journal—is not sufficiently articulated.

Several key findings (e.g., reinfection rates within 12–15 months, association with home slaughter and feeding of raw offal) largely confirm previously reported results. The manuscript would benefit from a clearer statement of what novel insight this study provides, whether in terms of study design, analytical approach, dog subpopulation stratification, or local transmission dynamics.

**Authors should:**

- Clearly define the specific knowledge gap addressed by this study.
- Avoid overstating novelty where findings are largely confirmatory.
- Explicitly differentiate this work from closely related recent studies.

**Response.** We would like to thank the reviewer for their insightful comments regarding the originality and contribution of our study.

Studying dog re‑infection with *Echinococcus granulosus* in endemic regions has important implications for the control and prevention of cystic echinococcosis, as re‑infected dogs serve as continuous sources of environmental contamination and sustain transmission to livestock and humans. Uderstanding the local re‑infection dynamics is essential for designing effective and region‑specific control measures. Re‑infection rates can vary widely depending on cultural practices, livestock management systems, slaughterhouse hygiene, the availability of infected offal, and dog feeding behaviors, meaning that findings from one area cannot safely be generalized to another. Conducting multiple studies within each endemic region allows researchers and public health authorities to determine how quickly dogs become re‑infected after deworming, identify high‑risk practices responsible for ongoing transmission, and evaluate whether current intervention programs such as routine praziquantel dosing or public education, are sufficient.

In Iran, despite being a country with diverse ecological zones and varying animal husbandry systems, only one study has investigated dog re‑infection, and it was performed in the southeastern region. While valuable, this single study cannot represent the complexities of transmission across the entire country, leaving substantial gaps in our understanding of how the parasite persists in other provinces. Addressing these gaps through region‑specific re‑infection studies is necessary to build a comprehensive national strategy, optimize surveillance, and tailor interventions to local risk factors, ultimately improving efforts to reduce both animal and human echinococcosis.

Our study explores new facets and points comparing to previous research:

1. In contrast to previous studies that primarily focused on farm dogs, our research expands the scope by including sheepdogs and guard dogs. These distinct groups, owing to their different behavioral patterns and access to contaminated resources, can play a more significant role in the dynamics of parasite transmission. By addressing this aspect, our study provides a deeper understanding of the transmission mechanisms within these canine populations.”
2. Larger sample size in the present study (more than twice that of comparable studie nationwide in Iran) and its geographical coverage of the northeastern region of Iran allow for a more comprehensive and regional perspective on disease epidemiology, potentially differing from other areas.
3. 15-month duration of re-infection study has enabled a more precise examination of seasonal dynamics and long-term changes compared to the 12-month periods of previous studies, thereby contributing to a better understanding of the transmission cycle.

Statements regarding the specific knowledge gap addressed by this study is added to the manuscript Introduction section (lines 95-110).

**2. Definition and potential misclassification of reinfection**

Reinfection is defined based on coproparasitological and PCR detection following praziquantel treatment. However, the manuscript does not sufficiently address the possibility of incomplete parasite elimination or delayed egg shedding, which could lead to misclassification.

The choice of a 7-day post-treatment interval to confirm clearance requires stronger justification, as residual infection or delayed clearance cannot be entirely excluded.

**Authors should:**

- Explicitly acknowledge this limitation in the Discussion.
- Discuss the potential for misclassification bias between treatment failure and true reinfection.
- Provide stronger literature-based justification for the clearance assessment timeline.

**Response.** We appreciate the reviewer’s important observation regarding the potential misclassification between treatment failure and true re‑infection in longitudinal *E. granulosus* studies. We fully agree that distinguishing these two events can be challenging due to the parasite’s prepatent period. We acknowledge that some degree of misclassification cannot be entirely excluded in field studies of canine echinococcosis due to the limitations of available diagnostic tools. We have now added statements in the limitations section explicitly noting this issue.

Re-infection misclassification bias cannot be entirely excluded in field studies of canine echinococcosis due to the limitations of available diagnostic tools. The definitive diagnosis of canine echinococcosis is complicated by the absence of a perfect gold standard diagnostic tool, necessitating careful consideration of each tool’s limitations. This underscores the importance of employing a combination of available methods, each with its own specific sensitivities and limitations, to achieve the most accurate assessment. However in our study, measures were implemented to minimize misclassification bias. The diagnostic method we used has high sensitivity and specificity, and we interpreted results cautiously to avoid overestimating re‑infection. Also, praziquantel is reported to achieve >99% efficacy against Echinococcus spp. within 24–48 hours (WHO/OIE, 2001) and all praziquantel treatments were administered directly by trained personnel under observation to ensure correct dosing and reduce the risk of incomplete treatment, thereby lowering the probability of treatment failure.

In response, we have added a dedicated clarification (limitation) in the Discussion section (lines 409–424).

**3. Statistical analysis and reporting**

The use of Cox proportional hazards models is appropriate; however, essential methodological details are missing.

**Specifically:**

- There is no indication that the proportional hazards assumption was tested.
- The process for variable selection in multivariable models is unclear.
- Potential collinearity between behavioral variables (e.g., home slaughter and offal feeding) is not addressed.

**Authors should:**

- Clearly state whether and how model assumptions were evaluated.
- Describe criteria for variable inclusion.
- Consider sensitivity analyses or alternative modeling approaches if assumptions are violated.

**Response.**

Thanks for the technical recommendation. The proportional hazard assumption was tested using Schoenfeld residual plots and no departure from PH assumption was seen. In total, data on eight independent variables were collected. It has been shown that regression models provide stable estimates if the sample size per number of coefficients is at least 10. Given the adequate sample size, we included all variables in the model to make a full adjustment. The variance inflation factor (VIF) was calculated, and no serious multicollinearity between independent variables was observed. We have added statements regarding these issues in the Methods section (lines 196–199 and 203-206).

**4. Interpretation and programmatic implications**

The discussion extends to broad control strategies (e.g., integrated One Health interventions, livestock vaccination), which were not evaluated in this study.

While such strategies are relevant, the manuscript should distinguish more clearly between evidence generated by the current study and broader recommendations derived from the literature.

**Authors should:**

- Temper conclusions to align more closely with the observational data.
- Clearly separate direct findings from inferred policy implications.
- Prioritize recommendations based on feasibility and relevance to the study setting.

**Response.** Thank you for this valuable comment. The Discussion section has been carefully revised and re-organized in line with your suggestions. We have clearly distinguished between findings derived directly from our data (**Evidence generated by the current study,** line 272-355) and broader interpretations or recommendations drawn from the literature (**Policy implications and recommendations,** line 357-408). The conclusions have been moderated to better reflect the observational nature of the study, and speculative or overly general statements have been reduced. In addition, policy-related recommendations have been refined to emphasize those that are most feasible and relevant to the specific study setting.

**5. Limited integration of genotyping results**

Although parasite genotyping is reported, the sample size is small, and the results are not analytically integrated into the main findings.

**Authors should:**

- Clarify the exploratory nature of the genotyping data.
- Avoid overinterpretation.
- Either better contextualize these results epidemiologically or reduce their emphasis.

**Response.** We thank the reviewer for this careful observation. We agree that the parasite genotyping results are based on a small sample size and acknowledge that this limits the analytical integration of these data into our main findings. Considering this, we have more clearly highlighted the exploratory nature of these genotyping findings in the Discussion section of the manuscript. Also, we have shortened the genotyping-related content and removed speculative claims regarding genotype-specific transmission dynamics. The text now presents the genotyping findings as descriptive observations that require validation in larger, adequately powered studies (line 346-355).

**Response to Reviewer #3**

Thanks for the constructive comments made by the reviewer in the manuscript text file. All the comments are considered and the main text is amended according to the reviewer’s comments and recommendations:

**ABSTRACT**

**Response.** Thank you, Amended. “In line 3, ‘in many regions of the world’ has been replaced by ‘**worldwide**’.

**AUTHOR SUMMARY**

**Response.** “The word ‘**Because**’ was removed from line 34, and consequently, the sentence was revised.”

**INTRODUCTION**

**Response.** “In line 51, the term ‘**Cystic echinococcosis**’ was revised according to the reviewer’s feedback.”

**Response.** “In line 53, the word ‘**affecting**’ was removed.”

**Response.** “In line 54, the relevant reference was added.”

**Response.** “In line 66, the word ‘**alone**’ was removed.”

**Response.** “In line 73, the sentence ‘**Studies indicate that the global dog population has surpassed 700 million, of which 75% are owned and unowned free-roaming dogs**.’ was removed, following the reviewer’s feedback.”

**Response.** “In line 76, the word ‘**Necessary**’ was removed.”

**Response.** Thank you, amended.

**Study design and Sample collection**

**Response.** “In line 145: The figure of **50%** was replaced with the phrase ‘**Half of the collected sample’**.”

**Comments.** In the Methodology you described that you use as molecular marker COX1. However, here you described that you compare the genotypes using SSU molecular marked. And the COX1???

**Response.** We thank you for your careful assessment. As described in the Methods section (line 171-175) “We used the cox primer to confirm the microscopic study of taeniid eggs and two other primers to investigate and confirm *Echinococcus granulosus*. Additionally, the SSU primer was utilized for genotyping.”

**Thank you.**
