# Supplementary material for: Reinfection dynamics of owned dogs with Echinococcus granulosus sensu lato in endemic rural areas of central Iran: Implications for cystic echinococcosis control
Source: PLoS Negl Trop Dis. 2026 Jun 29;20(6):e0014451. doi: 10.1371/journal.pntd.0014451 (PMC13327511; doi:10.1371/journal.pntd.0014451)
Supplement: Peer review history

# Author comment: Author response to Decision Letter 0

**DOI:** 10.1371/journal.pntd.0014451.r001

31 Jul 2025

Attachment Submitted filename: PNTD_SRM_Response_14040507.docx

# Peer review: Decision Letter 0

**DOI:** 10.1371/journal.pntd.0014451.r002

**Authors:** Peter U Fischer, Eduardo José Lopes-Torres

12 Oct 2025

PNTD-D-25-01258

Reinfection dynamics of owned dogs with Echinococcus granulosus in endemic rural areas of central Iran: Implications for cystic echinococcosis control

PLOS Neglected Tropical Diseases

Dear Dr. Fasihi Harandi,

Thank you for submitting your manuscript to PLOS Neglected Tropical Diseases. After careful consideration, we feel that it has merit but does not fully meet PLOS Neglected Tropical Diseases's publication criteria as it currently stands. Therefore, we invite you to submit a revised version of the manuscript that addresses the points raised during the review process.

Please submit your revised manuscript within 60 days Dec 11 2025 11:59PM. If you will need more time than this to complete your revisions, please reply to this message or contact the journal office at plosntds@plos.org. When you're ready to submit your revision, log on to https://www.editorialmanager.com/pntd/ and select the 'Submissions Needing Revision' folder to locate your manuscript file.

Please include the following items when submitting your revised manuscript:

* A rebuttal letter that responds to each point raised by the editor and reviewer(s). You should upload this letter as a separate file labeled 'Response to Reviewers'. This file does not need to include responses to any formatting updates and technical items listed in the 'Journal Requirements' section below.

* A marked-up copy of your manuscript that highlights changes made to the original version. You should upload this as a separate file labeled 'Revised Manuscript with Track Changes'.

* An unmarked version of your revised paper without tracked changes. You should upload this as a separate file labeled 'Manuscript'.

If you would like to make changes to your financial disclosure, competing interests statement, or data availability statement, please make these updates within the submission form at the time of resubmission. Guidelines for resubmitting your figure files are available below the reviewer comments at the end of this letter.

We look forward to receiving your revised manuscript.

Kind regards,

Eduardo José Lopes-Torres, Ph.D.

Academic Editor

PLOS Neglected Tropical Diseases

Peter Fischer

Section Editor

PLOS Neglected Tropical Diseases

Shaden Kamhawi

co-Editor-in-Chief

PLOS Neglected Tropical Diseases

orcid.org/0000-0003-4304-636XX

Paul Brindley

co-Editor-in-Chief

PLOS Neglected Tropical Diseases

orcid.org/0000-0003-1765-0002

**Additional Editor Comments:**

Reviewer #1:

Reviewer #2:

**Journal Requirements:**

If the reviewer comments include a recommendation to cite specific previously published works, please review and evaluate these publications to determine whether they are relevant and should be cited. There is no requirement to cite these works unless the editor has indicated otherwise.

1) Please ensure that the CRediT author contributions listed for every co-author are completed accurately and in full.

At this stage, the following Authors/Authors require contributions: Seyed Reza Mirbadie, Saeedeh Shamsaddini, Mohammad Taghi Rahimi, Mohammad Matini, Mohammad Ali Mohaghegh, Mohammad Reza Baneshi, Majid Fasihi Harandi, and Mohammad Fallah. Please ensure that the full contributions of each author are acknowledged in the "Add/Edit/Remove Authors" section of our submission form.

The list of CRediT author contributions may be found here: https://journals.plos.org/plosntds/s/authorship#loc-author-contributions

2) Please provide an Author Summary. This should appear in your manuscript between the Abstract (if applicable) and the Introduction, and should be 150-200 words long. The aim should be to make your findings accessible to a wide audience that includes both scientists and non-scientists. Sample summaries can be found on our website under Submission Guidelines:

https://journals.plos.org/plosntds/s/submission-guidelines#loc-parts-of-a-submission

3) Please upload all main figures as separate Figure files in .tif or .eps format. For more information about how to convert and format your figure files please see our guidelines:

https://journals.plos.org/plosntds/s/figures

4) We have noticed that you have uploaded Supporting Information files, but you have not included a list of legends. Please add a full list of legends for your Supporting Information files after the references list.

5) Some material included in your submission may be copyrighted. According to PLOSu2019s copyright policy, authors who use figures or other material (e.g., graphics, clipart, maps) from another author or copyright holder must demonstrate or obtain permission to publish this material under the Creative Commons Attribution 4.0 International (CC BY 4.0) License used by PLOS journals. Please closely review the details of PLOSu2019s copyright requirements here: PLOS Licenses and Copyright. If you need to request permissions from a copyright holder, you may use PLOS's Copyright Content Permission form.

Please respond directly to this email and provide any known details concerning your material's license terms and permissions required for reuse, even if you have not yet obtained copyright permissions or are unsure of your material's copyright compatibility. Once you have responded and addressed all other outstanding technical requirements, you may resubmit your manuscript within Editorial Manager.

Potential Copyright Issues:

i) Figure 1. Please (a) provide a direct link to the base layer of the map (i.e., the country or region border shape) and ensure this is also included in the figure legend; and (b) provide a link to the terms of use / license information for the base layer image or shapefile. We cannot publish proprietary or copyrighted maps (e.g. Google Maps, Mapquest) and the terms of use for your map base layer must be compatible with our CC BY 4.0 license.

Note: if you created the map in a software program like R or ArcGIS, please locate and indicate the source of the basemap shapefile onto which data has been plotted.

If your map was obtained from a copyrighted source please amend the figure so that the base map used is from an openly available source. Alternatively, please provide explicit written permission from the copyright holder granting you the right to publish the material under our CC BY 4.0 license.

If you are unsure whether you can use a map or not, please do reach out and we will be able to help you. The following websites are good examples of where you can source open access or public domain maps:

* U.S. Geological Survey (USGS) - All maps are in the public domain. (http://www.usgs.gov)

* PlaniGlobe - All maps are published under a Creative Commons license so please cite u201cPlaniGlobe, http://www.planiglobe.com, CC BY 2.0u201d in the image credit after the caption. (http://www.planiglobe.com/?lang=enl)

* Natural Earth - All maps are public domain. (http://www.naturalearthdata.com/about/terms-of-use/).

6) We note that your Data Availability Statement is currently as follows: "The data that support the findings of this study are available as Supporting Information file.". Please confirm at this time whether or not your submission contains all raw data required to replicate the results of your study. Authors must share the “minimal data set” for their submission. PLOS defines the minimal data set to consist of the data required to replicate all study findings reported in the article, as well as related metadata and methods (https://journals.plos.org/plosone/s/data-availability#loc-minimal-data-set-definition).

For example, authors should submit the following data:

1) The values behind the means, standard deviations and other measures reported;

2) The values used to build graphs;

3) The points extracted from images for analysis..

Authors do not need to submit their entire data set if only a portion of the data was used in the reported study.

If your submission does not contain these data, please either upload them as Supporting Information files or deposit them to a stable, public repository and provide us with the relevant URLs, DOIs, or accession numbers. For a list of recommended repositories, please see https://journals.plos.org/plosone/s/recommended-repositories.

If there are ethical or legal restrictions on sharing a de-identified data set, please explain them in detail (e.g., data contain potentially sensitive information, data are owned by a third-party organization, etc.) and who has imposed them (e.g., an ethics committee). Please also provide contact information for a data access committee, ethics committee, or other institutional body to which data requests may be sent. If data are owned by a third party, please indicate how others may request data access.

7) Please amend your detailed Financial Disclosure statement. This is published with the article. It must therefore be completed in full sentences and contain the exact wording you wish to be published.

1) State the initials, alongside each funding source, of each author to receive each grant. For example: "This work was supported by the National Institutes of Health (####### to AM; ###### to CJ) and the National Science Foundation (###### to AM)."

2) State what role the funders took in the study. If the funders had no role in your study, please state: "The funders had no role in study design, data collection and analysis, decision to publish, or preparation of the manuscript."

3) If any authors received a salary from any of your funders, please state which authors and which funders..

If you did not receive any funding for this study, please simply state: u201cThe authors received no specific funding for this work.u201d

8)Please send a completed 'Competing Interests' statement, including any COIs declared by your co-authors. If you have no competing interests to declare, please state "The authors have declared that no competing interests exist". Otherwise please declare all competing interests beginning with the statement "I have read the journal's policy and the authors of this manuscript have the following competing interests"

**Reviewers' Comments:**

Reviewer's Responses to Questions

**Key Review Criteria Required for Acceptance?**

As you describe the new analyses required for acceptance, please consider the following:

**Methods**

-Are the objectives of the study clearly articulated with a clear testable hypothesis stated?

-Is the study design appropriate to address the stated objectives?

-Is the population clearly described and appropriate for the hypothesis being tested?

-Is the sample size sufficient to ensure adequate power to address the hypothesis being tested?

-Were correct statistical analysis used to support conclusions?

-Are there concerns about ethical or regulatory requirements being met?

Reviewer #1: Review – Key Criteria for Acceptance

Overall, the study demonstrates methodological rigor and satisfactorily meets the main criteria for acceptance:

Objectives and Hypothesis

The study objectives are clearly articulated, with a well-defined and testable hypothesis. The rationale is presented in a way that highlights the relevance and significance of the research.

Study Design

The chosen design is appropriate to address the stated objectives. The methodological framework is well-structured, ensuring consistent and relevant data collection.

Study Population

The study population is clearly described and appropriate for testing the proposed hypothesis.

Sample Size

The sample size is sufficient to provide adequate statistical power, strengthening the robustness and reliability of the findings.

Statistical Analyses

The statistical methods applied are appropriate and correctly used, consistently supporting the study’s conclusions. Data handling is transparent and well-grounded.

Ethical and Regulatory Considerations

No concerns arise regarding ethical or regulatory compliance. The study adheres to good research practices and ethical standards.

In summary, the study presents solid methodological quality, appropriate analyses, and full ethical compliance, placing it in a favorable position for acceptance.

Reviewer #2: Although morphological analyses of eggs found in feces require a specialist to identify the eggs or even have the limitation do not identify in the species level, and genetic analysis can be used as a tool to complement integrative taxonomy, integrating morphological and molecular data to provide complete information. I suggest that the authors include images from parasitological examination of feces, with data from eggs identified by morphology, as well as morphometry. In addition, it is possible to compare how efficient the PCR methodology is for evaluating helminth infection.

The hypothesis of the study was not clear in the text especially in introduction.

The dising are appropriate.

The size of sample was adequate to test the objetctive of the study, as weel as the statisc analysis.

**********

**Results**

-Does the analysis presented match the analysis plan?

-Are the results clearly and completely presented?

-Are the figures (Tables, Images) of sufficient quality for clarity?

Reviewer #1: Results

The analysis presented is consistent with the original analysis plan, demonstrating methodological coherence throughout the study. The results are reported in a clear and comprehensive manner, ensuring that readers can easily understand the findings and their significance. Furthermore, the figures, including tables and images, are of good quality, effectively enhancing the clarity of the data presentation and supporting the interpretation of the results.

Reviewer #2: yes, the analysis match the plan.

The results are ok however needs major resivion

the fugures are not with sufficient quality

**********

**Conclusions**

-Are the conclusions supported by the data presented?

-Are the limitations of analysis clearly described?

-Do the authors discuss how these data can be helpful to advance our understanding of the topic under study?

-Is public health relevance addressed?

Reviewer #1: Conclusions

The conclusions are well supported by the data presented, reflecting the strength and consistency of the findings. The limitations of the analysis are clearly acknowledged, demonstrating transparency and strengthening the overall credibility of the study. The authors thoughtfully discuss how the data contribute to advancing knowledge on the topic, highlighting the study’s relevance within the broader scientific context. Moreover, the public health implications are explicitly addressed, underscoring the significance of the research in guiding practice and informing future initiatives.

Reviewer #2: yes

**********

**Editorial and Data Presentation Modifications?**

Use this section for editorial suggestions as well as relatively minor modifications of existing data that would enhance clarity. If the only modifications needed are minor and/or editorial, you may wish to recommend “Minor Revision” or “Accept”.

Reviewer #1: A minor revision is recommended, as the manuscript shows potential to enrich the scientific debate and contribute to a better understanding of Echinococcus granulosus sensu lato reinfection in dogs from rural areas.

Comments and Questions:

1. One of the most important recommendations I would like to highlight is the standardization of the scientific name currently used for Echinococcus granulosus. When referring to all genetic variants of the species, the correct terminology is Echinococcus granulosus sensu lato (Vuitton et al., 2020). Throughout the manuscript, including the title, the use of this term is inconsistent. Sometimes the correct terminology is used, while at other times, only Echinococcus granulosus appears. I recommend a careful review of the nomenclature to ensure uniformity.

2. Regarding the methodology for collecting fecal samples from herding dogs: how can one be certain that the feces collected at the resting places truly belong to the intended dog, considering these animals are free to roam the property? A more detailed explanation of this procedure would be helpful.

3. Another relevant question relates to the samples analyzed by molecular biology. Out of the 31 samples that tested positive for Echinococcus granulosus sensu lato, why were only 9 sequenced? Wouldn’t it be more informative to sequence all samples to better identify the genotypes involved in the study? This is especially important considering that different genotypes may behave differently during the course of infection.

4. On page 4 (Introduction), third line: replace the word “cardinal” with “main.”

5. On page 4 (Introduction), line 20: please add a reference for the statement: “Dogs can become…CE transmission.”

6. On page 4 (Introduction), line 25: please add a reference for the statement: “The frequency…endemic region.”

7. On page 6 (Materials and Methods), line 7: the citation of Wikipedia as a reference is not appropriate in a scientific manuscript. Is there a more reliable bibliographic source that could be used instead? I suggest replacing this reference.

8. On page 7 (Materials and Methods), line 21: where it reads “for identification of the dogs,” I suggest rephrasing to: “for identification of dogs during…”

9. In the title of Table 1, I recommend rephrasing to: “Study for the diagnosis of cystic canine echinococcosis...”

Reviewer #2: (No Response)

**********

**Summary and General Comments**

Use this section to provide overall comments, discuss strengths/weaknesses of the study, novelty, significance, general execution and scholarship. You may also include additional comments for the author, including concerns about dual publication, research ethics, or publication ethics. If requesting major revision, please articulate the new experiments that are needed.

Reviewer #1: The study investigates reinfection by Echinococcus granulosus sensu lato in herding and guard dogs following treatment with praziquantel. Fecal samples from 381 dogs in rural areas of Iran were analyzed, with 31 samples testing positive for Echinococcus eggs. After chemotherapy, fecal samples from the same animals were collected at different time points to assess reinfection. The results indicate that the likelihood of reinfection increased with time after treatment. Furthermore, herding dogs exhibited the highest reinfection rates, along with various risk factors contributing to reinfection.

Through molecular biology analysis, the presence of Echinococcus sensu stricto and Echinococcus canadensis was confirmed in some samples. Therefore, this study is original and provides updated data and evidence that support efforts to control cystic echinococcosis in countries facing this zoonotic disease.

Reviewer #2: (No Response)

**********

PLOS authors have the option to publish the peer review history of their article (what does this mean?). If published, this will include your full peer review and any attached files.

If you choose “no”, your identity will remain anonymous but your review may still be made public.

**Do you want your identity to be public for this peer review?**  For information about this choice, including consent withdrawal, please see our Privacy Policy.

Reviewer #1: No

Reviewer #2: No

[NOTE: If reviewer comments were submitted as an attachment file, they will be attached to this email and accessible via the submission site. Please log into your account, locate the manuscript record, and check for the action link "View Attachments". If this link does not appear, there are no attachment files.]

**Figure resubmission:**

While revising your submission, we strongly recommend that you use PLOS’s NAAS tool (https://ngplosjournals.pagemajik.ai/artanalysis) to test your figure files. NAAS can convert your figure files to the TIFF file type and meet basic requirements (such as print size, resolution), or provide you with a report on issues that do not meet our requirements and that NAAS cannot fix.

After uploading your figures to PLOS’s NAAS tool - https://ngplosjournals.pagemajik.ai/artanalysis, NAAS will process the files provided and display the results in the "Uploaded Files" section of the page as the processing is complete. If the uploaded figures meet our requirements (or NAAS is able to fix the files to meet our requirements), the figure will be marked as "fixed" above. If NAAS is unable to fix the files, a red "failed" label will appear above. When NAAS has confirmed that the figure files meet our requirements, please download the file via the download option, and include these NAAS processed figure files when submitting your revised manuscript.

**Reproducibility:**

To enhance the reproducibility of your results, we recommend that authors of applicable studies deposit laboratory protocols in protocols.io, where a protocol can be assigned its own identifier (DOI) such that it can be cited independently in the future. Additionally, PLOS ONE offers an option to publish peer-reviewed clinical study protocols. Read more information on sharing protocols at https://plos.org/protocols?utm_medium=editorial-email&utm_source=authorletters&utm_campaign=protocols

Attachment Submitted filename: Revision and recommendation final version.pdf

Attachment Submitted filename: Plos revision manuscript.docx

Attachment Submitted filename: PNTD-D-25-01258_reviewer.pdf

# Author comment: Author response to Decision Letter 1

**DOI:** 10.1371/journal.pntd.0014451.r003

28 Dec 2025

Attachment Submitted filename: Reviewers_Response_PNTD_14040920.docx

# Peer review: Decision Letter 1

**DOI:** 10.1371/journal.pntd.0014451.r004

**Authors:** Peter U Fischer, Eduardo José Lopes-Torres

23 Mar 2026

PNTD-D-25-01258R1

Reinfection dynamics of owned dogs with Echinococcus granulosus sensu lato in endemic rural areas of central Iran: Implications for cystic echinococcosis control

PLOS Neglected Tropical Diseases

Dear Dr. Fasihi Harandi,

Thank you for submitting your manuscript to PLOS Neglected Tropical Diseases. After careful consideration, we feel that it has merit but does not fully meet PLOS Neglected Tropical Diseases's publication criteria as it currently stands. Therefore, we invite you to submit a revised version of the manuscript that addresses the points raised during the review process.

Please submit your revised manuscript within by Apr 22 2026 11:59PM. If you will need more time than this to complete your revisions, please reply to this message or contact the journal office at plosntds@plos.org. When you're ready to submit your revision, log on to https://www.editorialmanager.com/pntd/ and select the 'Submissions Needing Revision' folder to locate your manuscript file.

Please include the following items when submitting your revised manuscript:

* A letter that responds to each point raised by the editor and reviewer(s). You should upload this letter as a separate file labeled 'Response to Reviewers'. This file does not need to include responses to any formatting updates and technical items listed in the 'Journal Requirements' section below.

* A marked-up copy of your manuscript that highlights changes made to the original version. You should upload this as a separate file labeled 'Revised Manuscript with Track Changes'.

* An unmarked version of your revised paper without tracked changes. You should upload this as a separate file labeled 'Manuscript'.

If you would like to make changes to your financial disclosure, competing interests statement, or data availability statement, please make these updates within the submission form at the time of resubmission. Guidelines for resubmitting your figure files are available below the reviewer comments at the end of this letter.

We look forward to receiving your revised manuscript.

Kind regards,

Eduardo José Lopes-Torres, Ph.D.

Academic Editor

PLOS Neglected Tropical Diseases

Peter Fischer

Section Editor

PLOS Neglected Tropical Diseases

Shaden Kamhawi

co-Editor-in-Chief

PLOS Neglected Tropical Diseases

orcid.org/0000-0003-4304-636XX

Paul Brindley

co-Editor-in-Chief

PLOS Neglected Tropical Diseases

orcid.org/0000-0003-1765-0002

**Journal Requirements:**

1) Please ensure that the CRediT author contributions listed for every co-author are completed accurately and in full.

At this stage, the following Authors/Authors require contributions: Seyed Reza Mirbadie, Saeedeh Shamsaddini, Mohammad Taghi Rahimi, Mohammad Matini, Mohammad Ali Mohaghegh, Mohammad Reza Baneshi, Majid Fasihi Harandi, and Mohammad Fallah. Please ensure that the full contributions of each author are acknowledged in the "Add/Edit/Remove Authors" section of our submission form.

The list of CRediT author contributions may be found here: https://journals.plos.org/plosntds/s/authorship#loc-author-contributions

2) Please upload all main figures as separate Figure files in .tif or .eps format. For more information about how to convert and format your figure files please see our guidelines:

https://journals.plos.org/plosntds/s/figures

3) Please amend your detailed Financial Disclosure statement. This is published with the article. It must therefore be completed in full sentences and contain the exact wording you wish to be published.

- State the initials, alongside each funding source, of each author to receive each grant. For example: "This work was supported by the National Institutes of Health (####### to AM; ###### to CJ) and the National Science Foundation (###### to AM)."

- State what role the funders took in the study. If the funders had no role in your study, please state: "The funders had no role in study design, data collection and analysis, decision to publish, or preparation of the manuscript.".

If you did not receive any funding for this study, please simply state: u201cThe authors received no specific funding for this work.u201d

**Reviewers' Comments:**

[NOTE: If reviewer comments were submitted as an attachment file, they will be attached to this email and accessible via the submission site. Please log into your account, locate the manuscript record, and check for the action link "View Attachments". If this link does not appear, there are no attachment files.]

**Figure resubmission:**

While revising your submission, we strongly recommend that you use PLOS’s NAAS tool (https://ngplosjournals.pagemajik.ai/artanalysis) to test your figure files. NAAS can convert your figure files to the TIFF file type and meet basic requirements (such as print size, resolution), or provide you with a report on issues that do not meet our requirements and that NAAS cannot fix.

After uploading your figures to PLOS’s NAAS tool - https://ngplosjournals.pagemajik.ai/artanalysis, NAAS will process the files provided and display the results in the "Uploaded Files" section of the page as the processing is complete. If the uploaded figures meet our requirements (or NAAS is able to fix the files to meet our requirements), the figure will be marked as "fixed" above. If NAAS is unable to fix the files, a red "failed" label will appear above. When NAAS has confirmed that the figure files meet our requirements, please download the file via the download option, and include these NAAS processed figure files when submitting your revised manuscript.

**Reproducibility:**

To enhance the reproducibility of your results, we recommend that authors of applicable studies deposit laboratory protocols in protocols.io, where a protocol can be assigned its own identifier (DOI) such that it can be cited independently in the future. Additionally, PLOS ONE offers an option to publish peer-reviewed clinical study protocols. Read more information on sharing protocols at https://plos.org/protocols?utm_medium=editorial-email&utm_source=authorletters&utm_campaign=protocols

Attachment Submitted filename: Revision and recommendation final version.docx

Attachment Submitted filename: PNTD-D-25-01258_R1.pdf

# Author comment: Author response to Decision Letter 2

**DOI:** 10.1371/journal.pntd.0014451.r005

5 May 2026

Attachment Submitted filename: Reviewers_Response_PNTD_14050213.docx

# Peer review: Decision Letter 2

**DOI:** 10.1371/journal.pntd.0014451.r006

**Authors:** Peter U Fischer, Eduardo José Lopes-Torres

9 Jun 2026

Dear Professor Fasihi Harandi,

We are pleased to inform you that your manuscript 'Reinfection dynamics of owned dogs with Echinococcus granulosus sensu lato in endemic rural areas of central Iran: Implications for cystic echinococcosis control' has been provisionally accepted for publication in PLOS Neglected Tropical Diseases.

Before your manuscript can be formally accepted you will need to complete some formatting changes, which you will receive in a follow up email. A member of our team will be in touch with a set of requests.

Please note that your manuscript will not be scheduled for publication until you have made the required changes, so a swift response is appreciated.

IMPORTANT: The editorial review process is now complete. PLOS will only permit corrections to spelling, formatting or significant scientific errors from this point onwards. Requests for major changes, or any which affect the scientific understanding of your work, will cause delays to the publication date of your manuscript.

Should you, your institution's press office or the journal office choose to press release your paper, you will automatically be opted out of early publication. We ask that you notify us now if you or your institution is planning to press release the article. All press must be co-ordinated with PLOS.

Thank you again for supporting Open Access publishing; we are looking forward to publishing your work in PLOS Neglected Tropical Diseases.

Best regards,

Eduardo José Lopes-Torres, Ph.D.

Academic Editor

PLOS Neglected Tropical Diseases

Peter Fischer

Section Editor

PLOS Neglected Tropical Diseases

Shaden Kamhawi

co-Editor-in-Chief

PLOS Neglected Tropical Diseases

orcid.org/0000-0003-4304-636XX

Paul Brindley

co-Editor-in-Chief

PLOS Neglected Tropical Diseases

orcid.org/0000-0003-1765-0002

***********************************************************

Dear Authors,

We are pleased to inform you that your manuscript has been accepted for publication.

However, it is important to note that both reviewers suggested several minor revisions and improvements. As these comments can be adequately addressed through adjustments to the final version of the manuscript, we kindly ask that you carefully consider and incorporate all reviewer recommendations before submitting the final version for publication.

Best regards,

Eduardo Torres

Reviewer's Responses to Questions

**Key Review Criteria Required for Acceptance?**

As you describe the new analyses required for acceptance, please consider the following:

**Methods**

-Are the objectives of the study clearly articulated with a clear testable hypothesis stated?

-Is the study design appropriate to address the stated objectives?

-Is the population clearly described and appropriate for the hypothesis being tested?

-Is the sample size sufficient to ensure adequate power to address the hypothesis being tested?

-Were correct statistical analysis used to support conclusions?

-Are there concerns about ethical or regulatory requirements being met?

Reviewer #1: The study objectives are clearly stated and scientifically testable, although a formal hypothesis is not explicitly presented. The longitudinal cohort design is appropriate for investigating reinfection dynamics and associated risk factors following praziquantel treatment. The study population is clearly described and includes epidemiologically relevant groups of owned sheepdogs and guard dogs from endemic rural areas. The sample size appears adequate for the primary analyses and survival models performed, although genotype-related findings remain exploratory due to the limited number of sequenced samples. Appropriate statistical analyses were applied, including Kaplan–Meier survival analysis and Cox proportional hazards regression, and the conclusions are adequately supported by the data presented. No major ethical or regulatory concerns were identified, as ethical approval and owner consent procedures were appropriately reported.

Reviewer #2: yes

yes

yes

yes

yes

yes

I suggest changing the map and highlight the study localities, as well as distinguish in the map the dogs.

**********

**Results**

-Does the analysis presented match the analysis plan?

-Are the results clearly and completely presented?

-Are the figures (Tables, Images) of sufficient quality for clarity?

Reviewer #1: The analyses presented are consistent with the study objectives and methodological approach described in the manuscript. The use of Kaplan–Meier survival analysis and Cox proportional hazards regression appropriately matches the proposed analytical framework for evaluating reinfection dynamics and associated risk factors. Overall, the results are clearly presented and logically organized, with findings adequately described in both the text and tables. The progression of reinfection over time is easy to follow, and the statistical outcomes are appropriately reported. The tables and figures are generally of sufficient quality and contribute positively to the clarity of the manuscript. In particular, the survival curves and summary tables effectively support the interpretation of the findings. Minor improvements in figure formatting and reduction of repetitive information in some sections could further enhance readability, but no major concerns regarding data presentation were identified.

Reviewer #2: yes

yes

yes

**********

**Conclusions**

-Are the conclusions supported by the data presented?

-Are the limitations of analysis clearly described?

-Do the authors discuss how these data can be helpful to advance our understanding of the topic under study?

-Is public health relevance addressed?

Reviewer #1: The conclusions are generally well supported by the data presented and are consistent with the findings of the longitudinal analyses. The authors appropriately conclude that reinfection of dogs with *Echinococcus granulosus sensu lato* remains a major challenge for cystic echinococcosis control in endemic regions and that dog deworming alone is insufficient when environmental and behavioral risk factors persist. The limitations of the study are adequately acknowledged, particularly regarding the potential for reinfection misclassification, the absence of a perfect diagnostic gold standard, and the limited number of sequenced samples for genotype analysis. Furthermore, the manuscript clearly discusses how the findings contribute to advancing the understanding of reinfection dynamics, risk factors, and transmission persistence in endemic rural settings. The public health relevance is also appropriately addressed, especially through the discussion of One Health strategies, environmental contamination, home slaughter practices, and the implications for sustainable control programs targeting both animal and human cystic echinococcosis.

Reviewer #2: yes

yes

yes

yes

**********

**Editorial and Data Presentation Modifications?**

Use this section for editorial suggestions as well as relatively minor modifications of existing data that would enhance clarity. If the only modifications needed are minor and/or editorial, you may wish to recommend “Minor Revision” or “Accept”.

Reviewer #1: The manuscript has substantially improved after revision and only minor editorial modifications are still recommended. Some sections of the Discussion could be slightly condensed to reduce repetition and improve readability. In addition, minor grammatical inconsistencies and language polishing remain necessary throughout the text. The replacement of direct website links in the “Study Area” section with properly formatted institutional or governmental references would further strengthen the manuscript presentation. The tables and figures are clear and appropriate, and no major concerns regarding data presentation remain. Overall, the remaining issues are editorial in nature and do not affect the scientific quality of the study. Therefore, the manuscript is suitable for publication after minor revision.

Reviewer #2: The manuscript is interesting, especially as it addresses E. granulosus infection in dogs, a zoonotic helminth.

The authors have made the revisions as suggested in the previous corrections. Therefore, I consider the manuscript "accept".

**********

**Summary and General Comments**

Use this section to provide overall comments, discuss strengths/weaknesses of the study, novelty, significance, general execution and scholarship. You may also include additional comments for the author, including concerns about dual publication, research ethics, or publication ethics. If requesting major revision, please articulate the new experiments that are needed.

Reviewer #1: The revised manuscript presents a scientifically relevant and methodologically sound longitudinal investigation on the reinfection dynamics of *Echinococcus granulosus sensu lato* in owned dogs from endemic rural areas of Iran. The study addresses an important and still insufficiently explored aspect of cystic echinococcosis epidemiology, particularly regarding the persistence of transmission after praziquantel treatment and the challenges this poses for sustainable control programs.

The manuscript has improved substantially following revision. The authors adequately addressed most of the concerns raised in the previous review round, including improvement of taxonomic nomenclature consistency, clarification of sample collection procedures, expansion of methodological details, inclusion of additional references, and a more balanced discussion of the limitations of the study. The use of a longitudinal design, combined parasitological and molecular diagnostic approaches, and survival analyses represents an important strength of the study. The identification of risk factors associated with reinfection, particularly home slaughter practices and feeding behavior, adds important epidemiological and public health value to the work.

The study contributes meaningful data to the understanding of canine echinococcosis transmission dynamics and reinforces the importance of integrated One Health control strategies in endemic regions. The discussion appropriately contextualizes the findings within the existing literature and highlights the implications for public health policies and control programs.

Some minor editorial issues remain, including occasional grammatical inconsistencies, repetitive passages in the Discussion section, and minor formatting improvements for institutional references. However, these issues do not compromise the scientific quality or overall contribution of the manuscript.

Overall, the study is original, relevant, and suitable for publication after minor editorial revision.

Reviewer #2: (No Response)

**********

PLOS authors have the option to publish the peer review history of their article (what does this mean?). If published, this will include your full peer review and any attached files.

If you choose “no”, your identity will remain anonymous but your review may still be made public.

**Do you want your identity to be public for this peer review?**  For information about this choice, including consent withdrawal, please see our Privacy Policy.

Reviewer #1: No

Reviewer #2: No

Attachment Submitted filename: Secon revision.pdf

# Editor report: Acceptance letter

**DOI:** 10.1371/journal.pntd.0014451.r007

**Authors:** Peter U Fischer, Eduardo José Lopes-Torres

Dear Professor Fasihi Harandi,

We are delighted to inform you that your manuscript, "Reinfection dynamics of owned dogs with Echinococcus granulosus sensu lato in endemic rural areas of central Iran: Implications for cystic echinococcosis control," has been formally accepted for publication in PLOS Neglected Tropical Diseases.

We have now passed your article onto the PLOS Production Department who will complete the rest of the publication process. All authors will receive a confirmation email upon publication.

The corresponding author will soon be receiving a typeset proof for review, to ensure errors have not been introduced during production. Please review the PDF proof of your manuscript carefully, as this is the last chance to correct any scientific or type-setting errors. Please note that major changes, or those which affect the scientific understanding of the work, will likely cause delays to the publication date of your manuscript. Note: Proofs for Front Matter articles (Editorial, Viewpoint, Symposium, Review, etc...) are generated on a different schedule and may not be made available as quickly.

Soon after your final files are uploaded, the early version of your manuscript will be published online unless you opted out of this process. The date of the early version will be your article's publication date. The final article will be published to the same URL, and all versions of the paper will be accessible to readers.

For Research Articles, you will receive an invoice from PLOS for your publication fee after your manuscript has reached the completed accept phase. If you receive an email requesting payment before acceptance or for any other service, this may be a phishing scheme. Learn how to identify phishing emails and protect your accounts at https://explore.plos.org/phishing.

Thank you again for supporting open-access publishing; we are looking forward to publishing your work in PLOS Neglected Tropical Diseases.

Best regards,

Shaden Kamhawi

co-Editor-in-Chief

PLOS Neglected Tropical Diseases

Paul Brindley

co-Editor-in-Chief

PLOS Neglected Tropical Diseases
